# Modeling genotype-by-environment interactions across climatic conditions reveals environment-specific genomic regions and candidate genes underlying feed efficiency traits in tropical beef cattle

**DOI:** 10.1038/s41598-025-33952-1

**Published:** 2026-01-05

**Authors:** João B. Silva Neto, Luiz F. Brito, Lucio F. M. Mota, Gustavo R. D. Rodrigues, Fernando Baldi

**Affiliations:** 1 Department of Animal Science, School of Agricultural and Veterinarian Sciences (FCAV), São Paulo State University (UNESP), Jaboticabal, SP 14884-900 Brazil; 2 Department of Animal Sciences, Purdue University, West Lafayette, IN 47907 USA; 3 Department of Animal Science, Faculty of Animal Science and Food Engineering, University of São Paulo, Pirassununga, SP 13635-900 Brazil

**Keywords:** Climate resilience, Dry matter intake, Functional enrichment, Nellore cattle, Residual feed intake, Temperature-humidity index, Tropical environments, Computational biology and bioinformatics, Ecology, Ecology, Genetics

## Abstract

Heat stress represents a major limitation for livestock production systems, negatively affecting feed efficiency, animal health and welfare, and overall performance. In this context, the objective of this study was to identify genomic regions, candidate genes, biological pathways, and functional networks associated with dry matter intake (DMI) and residual feed intake (RFI) in Nellore cattle exposed to varying levels of thermal stress. The dataset comprised records from 22,838 animals, with genotypes available for 18,567 individuals. The data were collected during 296 feed efficiency trials between 2011 and 2023 across 21 Brazilian farms. Genome-wide association studies (GWAS) were performed using the single-step GBLUP (ssGBLUP) approach to account for genotype-by-environment (G×E) interactions in Nellore cattle. Environmental variation was modeled using the temperature-humidity index (THI) as the environmental gradient, with analyses stratified across three environmental gradients (EG): low (THI = 66), medium (THI = 74), and high (THI = 81). Fifty-one SNPs were significantly associated with RFI, including 27 shared across all three EGs, 10 exclusive to the low EG, one to the high EG, and 13 shared between the moderate and high EGs. These associations were mapped to 44 candidate genes, with 19 genes commonly identified across all EGs, including key candidates such as *PIPOX*, *GTF2F2*, *KCTD4*, *MYO18A*, and *NFIA*. For DMI, 136 significant SNPs were identified: 12 and 39 exclusive to the low and moderate EGs, respectively; 28 shared across all EGs; and 57 shared between the moderate and high EGs. These variants were linked to 58 candidate genes, of which 19 were common to all EGs, including *NCAPG*, *LCORL*, *FAM13A*, *HERC3*, *CCND1*, and *FGF19*. Gene network analyses revealed a clear reconfiguration of interaction structures across thermal gradients, particularly for RFI, where gene connectivity declined with increasing THI levels. For DMI, gene networks remained highly integrated, especially in the lowest THI level. Functional annotation highlighted both conserved and environment-specific regulatory architectures, involving key biological processes such as growth regulation, lipid and protein metabolism, intracellular signaling, stress response, and neuroendocrine control. These findings uncover the environmental sensitivity of RFI and DMI, highlight the complex and dynamic genomic basis of these traits under varying climatic conditions, and support the identification of candidate genes for genomic selection programs aiming to enhance climatic resilience in tropical beef cattle.

**Supplementary Information:**

The online version contains supplementary material available at 10.1038/s41598-025-33952-1.

## Background

Environmental stressors, particularly heat stress, impose significant challenges on livestock by triggering complex physiological and molecular responses that compromise animal health, welfare, and productivity^1,2^. Among the various indicators to quantify heat stress, the temperature-humidity index (THI) remains the most widely adopted, as it integrates temperature and relative humidity into a single descriptor of environmental stress^3–5^. Exposure to elevated THI levels has been associated with altered gene expression patterns^5–7^, dysregulation of metabolic pathways^8^, and impairment of key physiological functions, including immune response^2,9^, reproductive performance^10–12^, and nutrient metabolism^1,13^. These stress-induced modifications occur at multiple biological levels, ranging from transcriptional and post-transcriptional regulation to endocrine signaling, contributing to phenotypic variability among animals^14–17^. Furthermore, there is growing evidence that thermal stress may modulate the expression of genetic merit, directly affecting the response to selection under varying environmental conditions^18,19^. This highlights the importance of genotype-by-environment (G×E) interactions, in which the magnitude and direction of genetic effects vary depending on environmental conditions.

The intensification of climate change poses a major challenge to the sustainability of beef production systems, particularly in tropical environments where animals are continuously exposed to heat stress conditions^13,20^. Among the traits most sensitive to heat stress is feed efficiency^11,21^, which directly influences profitability, environmental sustainability, and resource allocation. Heat stress can compromise feed intake, alter energy partitioning, and reduce metabolic efficiency, thereby amplifying phenotypic variability^1,21,22^ and consequently variation in genetic merit. Consequently, identifying animals that maintain superior performance under thermal stress conditions becomes a strategic objective for breeding programs in tropical regions.

Genome-wide association studies (GWAS), when integrated with environmental descriptors such as THI, can enable the detection of genomic regions and candidate genes associated with resilience and adaptation to heat stress^23^. These approaches not only advance our understanding of the genetic background of feed efficiency under heat stress but also support the development of more precise genomic selection strategies, targeting animals that are both efficient and robust across diverse climatic scenarios. Furthermore, integrating environmental sensitivity into GWAS allows the identification of SNP-by-environment (SNP x E) interactions, unraveling the environment-dependent genetic effects that are often masked in conventional analyses^23–25^. Understanding how genomic regions influence response to environmental variability is therefore essential for advancing more precise and climate-resilient breeding programs.

In a previous study, Silva Neto et al.^19^ investigated G×E interactions for feed efficiency traits in Nellore cattle using a bi-trait genomic reaction norm model, considering THI as an environmental descriptor. Their results demonstrated that the genetic expression of dry matter intake (DMI) and residual feed intake (RFI) is sensitive to heat stress, with both heritability estimates and additive genetic variance declining under high THI conditions. These findings highlight the importance of incorporating environmental sensitivity into genetic evaluations to improve the selection of animals that remain feed efficient under thermally stressful conditions. However, no previous GWAS have evaluated the genetic background of DMI and RFI in Nellore cattle while explicitly accounting for environmental variation through distinct THI levels^26^. Therefore, the main objectives of this study were to: (i) perform a GWAS accounting for GxE interactions for RFI and DMI in Nellore cattle under varying levels of thermal conditions (low, medium, and high) according to the THI; and (ii) to annotate candidate genes and conduct functional enrichment analyses to elucidate the biological processes and molecular mechanisms associated with thermal resilience in feed efficiency traits.

## Materials and methods

### Field data and phenotypic information

Individual feed intake records were measured on 22,838 Nellore animals (16,233 males and 6,605 females) from 2011 to 2023. The datasets were provided by the National Association of Breeders and Researchers (ANCP, Ribeirão Preto, SP, Brazil; www.ancp.org.br)*.* Data originated from 296 feeding trials performed in 21 farms distributed in different Brazilian regions. Phenotypic information was available for DMI and RFI, following the standardized protocols for measuring individual feed intake in beef cattle described by Mendes et al.^27^. The feeding trials were performed in group pens with animals grouped by sex and age, with feed intake automatically recorded using the GrowSafe (www.vytelle.com) and Intergado (www.intergado.com) feeding systems. Each performance trial was conducted using a single feeding system brand and the same data collection protocol, ensuring that all animals within the same group were evaluated under the same recording conditions. Detailed descriptions of diet composition, management, and the evaluated traits are provided in Silva Neto et al.^28^. Descriptive statistics for the traits studied and environmental descriptor (THI) are reported in Table 1.

**Table 1:** Descriptive statistics for residual feed intake (RFI), dry matter intake (DMI), and temperature and humidity index (THI) during feed efficiency trials in Nellore cattle.

Variable	RFI (kg/day)	DMI (kg/day)	THI
Number of records	22,838	22,838	239
Average	0.000	8.530	74.37
Standard deviation	0.842	2.151	3.52
Minimum	− 7.109	2.519	66.86
Maximum	6.940	20.658	81.66
Feeding trials information			
Number of trials with only males	209		
Number of trials with only females	87		
Animals in the pedigree	46,383		
Sires	2,816		
Dams	21,749		
Sires with progeny records	817		
Dams with progeny records	10,339		
Number of contemporary groups	742		

The herds are genetically connected through the extensive use of common sires via artificial insemination, with at least five genetic links across feeding trials, as confirmed using the AMC package^29^. The animals were raised on pasture-based systems, with a predominance of the *Urochloa brizantha cv* forage. The commercial herds adopted different nutritional practices with some farms providing protein and mineral supplementation, especially during the dry season, while others provided only urea supplementation^28^.

### Genomic data

A total of 18,567 animals born between 2014 and 2022 were genotyped with a SNP panel containing 65,414 markers (Clarifide^®^ Nelore 3.0, Zoetis, Kalamazoo, MI). The genotypes were imputed to a high-density (HD) SNP panel (Illumina BovineHD; San Diego, CA, USA) containing 735,964 autosomal markers using the Fimpute 3.0 software^30^. Before genotype imputation, we removed non-autosomal markers and autosomal SNPs with GenCall < 0.60 to remove genotyping problems^31^.

The reference population for genotype imputation consisted of 963 representative sires from the main Nellore lineages in Brazil (i.e., Karvadi, Golias, Godhavari, Taj Mahal, Akasamu, and Nagpur), born between 1995 and 2015 and genotyped with the Illumina BovineHD BeadChip (Illumina Inc., San Diego, CA, USA). The quality control in imputed genotypes was performed using the qcf90 software^32^, removing samples and SNPs with call rate < 0.90, markers with Mendelian conflicts > 1%, extreme deviations from Hardy-Weinberg equilibrium (*p*-value ≤ 10^− 8^), and minor allele frequency (MAF) < 0.05. After filtering, 18,567 genotyped animals and 452,283 SNPs remained for further analyses.

### Weather data

Meteorological data corresponding to the days when the evaluated traits were recorded (2011–2023) were retrieved from NASA POWER (https://power.larc.nasa.gov/) based on each herd’s geographical coordinates. The addresses for each herd were converted to latitude and longitude coordinates using Google Maps Geocoding (https://developers.google.com/maps/documentation/geocoding*).*

The Temperature–Humidity Index (THI) was calculated according to NRC^33^:

$$\:THI=\left[\left(1.8\:x\:{T}_{db}+32\right)\right]-(0.55-\left(0.0055\:x\:RH\right)\:x\:\left(1.8\:x\:{T}_{db}-26\right))]$$

where $\:{T}_{db}$ is the dry bulb temperature (in Celsius degrees) and $\:RH$ is the relative humidity. This equation has been frequently applied in similar studies to evaluate the GxE across heat stress conditions^19,34–36^. As THI is a composite index that weights both temperature and relative humidity, different T–RH pairs can lead to the same THI value. For example, combinations such as 33 °C with ~ 20% RH, or 27 °C with ~ 65% RH, yield THI values very close to 76. The annual mean variation of the THI during the years in which feed efficiency trials were conducted, the seasonal distribution of THI values, and the relative frequency of instances in which THI was equal to or exceeded 76 (threshold indicating the onset of thermal stress for the Nellore breed) are detailed in Silva Neto et al.^19^. These data provide important environmental context, emphasizing the intensity and frequency of heat stress exposure experienced by the animals throughout the feed efficiency trials.

Given the THI range observed during the feed efficiency trials (~ 66–81), and with the aim of facilitating the biological interpretation of the results, we selected three representative points along the environmental gradient to present and contrast the GWAS results: THI 66 (the mildest/thermoneutral condition available), THI 74 (close to the center of the gradient, where the first Legendre coefficient was ≈ 0 and very close to the reported onset of heat stress in Nellore cattle), and THI 81 (the condition of greatest thermal challenge within the dataset).

### Genome-wide association analyses (GWAS)

GWAS were conducted independently for each EG (Low = THI 66, Medium = THI 74, and High = THI 81). The same population of 22,838 Nellore cattle, of which 18,567 were genotyped, was used in all analyses. No phenotypic stratification by EG was applied, instead, the environment-specific variance components previously estimated using a single-step genomic reaction norm model for the same population and described in detail by Silva Neto et al.^19^, were used as inputs for the respective GWAS models. Integrating these variance components ensured methodological consistency between the genetic parameter estimates and the environmental conditions under which the phenotypes were expressed, so that association tests were carried out under the same environmental structure in which genetic parameters were obtained, making SNP detection consistent with the previously modeled G×E structure along the THI gradient and preserving coherence among phenotypic adjustments, environmental characterization, and marker detection. Additional descriptive statistics by THI classes and the distribution of records along the THI gradient, which were used to fit the reaction norm model in the previous study, are presented in Silva Neto et al.^19^.

The single-step genome-wide association study (ssGWAS) method proposed by Wang et al.^37^ was used for the analyses. The general linear mixed model used for the traits studied was:

$$\:\varvec{y}=\varvec{X}\varvec{\beta\:}+\varvec{Z}\varvec{a}+\varvec{e}$$

where ***y*** is the vector of phenotypic information for DMI and RFI; ***X*** is an incidence matrix relating the phenotypes to the fixed effects; $\:\varvec{\beta\:}$ is the vector of fixed effect of CG, which was defined by concatenating the effects of farm, year and season of the feeding trial, and sex (males and females were evaluated in separate groups), and the age of the animal at the beginning of the feed efficiency trials as a linear covariate; ***Z*** is the incidence matrix relating the records to the additive genetic effects; ***a*** is the vector of random animal additive genetic effects with ***a*** ∼N(0,**H**$\:{{\upsigma\:}}_{\text{u}}^{2}$​), and ***e*** is the vector of residual effects with ***e*** **∼**N**(**0, **I**$\:{{\upsigma\:}}_{\text{e}}^{2}$**​)**.

The inverse of the hybrid relationship matrix $\:{\mathbf{H}}^{-1}$ was constructed as^38^:

$$\:{\mathbf{H}}^{-1}={\mathbf{A}}^{-1}+\left[\begin{array}{cc}0&\:0\\\:0&\:{\mathbf{G}}^{-1}-{\mathbf{A}}_{22}^{-1}\end{array}\right]$$

where $\:{\mathbf{A}}^{-1}$ is the inverse of the pedigree-based relationship matrix; $\:{\mathbf{A}}_{22}^{-1}\:$represents the inverse of the relationship matrix based on pedigree for the genotyped animals; and $\:{\mathbf{G}}^{-1}$ is the inverse of the genomic relationship matrix obtained according to the first method proposed by VanRaden^39^.

SNP effects were estimated by back-solving from the genomic estimated breeding values (GEBVs) of genotyped animals, following the procedure described by Wang et al.^37^ and implemented in the postGSf90^40^ of the BLUPf90 suite. All SNPs were considered to contribute equally to the total additive genetic variance, and no weighting scheme was applied. The SNP effects were derived as:

$$\hat{\varvec{u}} = {\mathbf{Z}}^{\prime } \left( {{\mathbf{ZZ}}^{\prime } } \right)^{{ - 1}} \hat{\varvec{a}}$$

where ***û*** is the vector of estimated additive genetic effects for the SNP markers; $\:\widehat{a}$ is the vector of GEBVs for the genotyped animals; ***Z*** is the centered genotype matrix (each genotype coded as 0, 1, or 2, centered by subtracting 2p, where p is the allele frequency of the reference allele). All computations were performed using postGSf90, which executes this back solving algorithm internally.

The *p*-value of the SNP effect was calculated based on the prediction error variance as^40^:

$$P_{i} = 2\left( {1 - \Phi \left( {\left| {\frac{{a_{i} }}{{sd\left( {a_{i} } \right)}}} \right|} \right)} \right)$$

where *α*_*i*_ is the SNP effect estimate; *sd* is the standard deviation; and *Φ* is the standard normal cumulative distribution function. The *p*-values were generated by back-solving the SNP effects from the GEBVs.

After performing the GWAS, the genomic inflation factor (λ_GC_) was calculated to assess potential biases in the statistics, such as those arising from population stratification. The λ_GC_ value was computed as the ratio between the median of the observed test statistic distribution and its expected median, with a 95% confidence interval subsequently derived^41^. Multiple testing correction was applied using the Bonferroni method (α = 0.05)^42^, resulting in a genome-wide significance threshold of at *P* = 0.05 / 452,283 (*P* < 1.11 × 10⁻⁷), equivalent to − log₁₀(P) ≈ 6.96. To avoid type I and II errors, a chromosome-wide significance threshold was considered based on the number of independent chromosomal segments (M_e_)^43^ as: M_e_ = 2N_e_Lk / log (N_e_L), where M_e_ is a function of effective population size; L is the length of each chromosome in Morgans; and k is the number of chromosomes. N_e_ was set to 100 based on linkage disequilibrium patterns observed in the population^25^. Quantile-quantile (Q-Q) plots were created using the CMplot R package^44^.

### Gene enrichment analyses

The annotation of candidate genes was performed using the GALLO package^45^ available in R (R Core Team). For that, a window of 100Kb up and downstream from the significant SNP marker was used considering the assembly *Bos taurus* ARS-UCD1.2 as the reference genome^46^. After annotation, the positional candidate genes were subjected to functional enrichment analysis using the “clusterProfiler” R package^47^. Gene Ontology (GO) terms including biological processes (BP), metabolic functions (MF), and cellular components (CC), as well as the Kyoto Encyclopedia of Genes and Genomes (KEGG) pathways (*p* < 0.05) were used to explore the biological relevance of the associated genomic regions. Interactions between protein-coding genes were predicted using the STRING database with default settings^48^.

## Results

### Significant markers

**Fig. 1:**
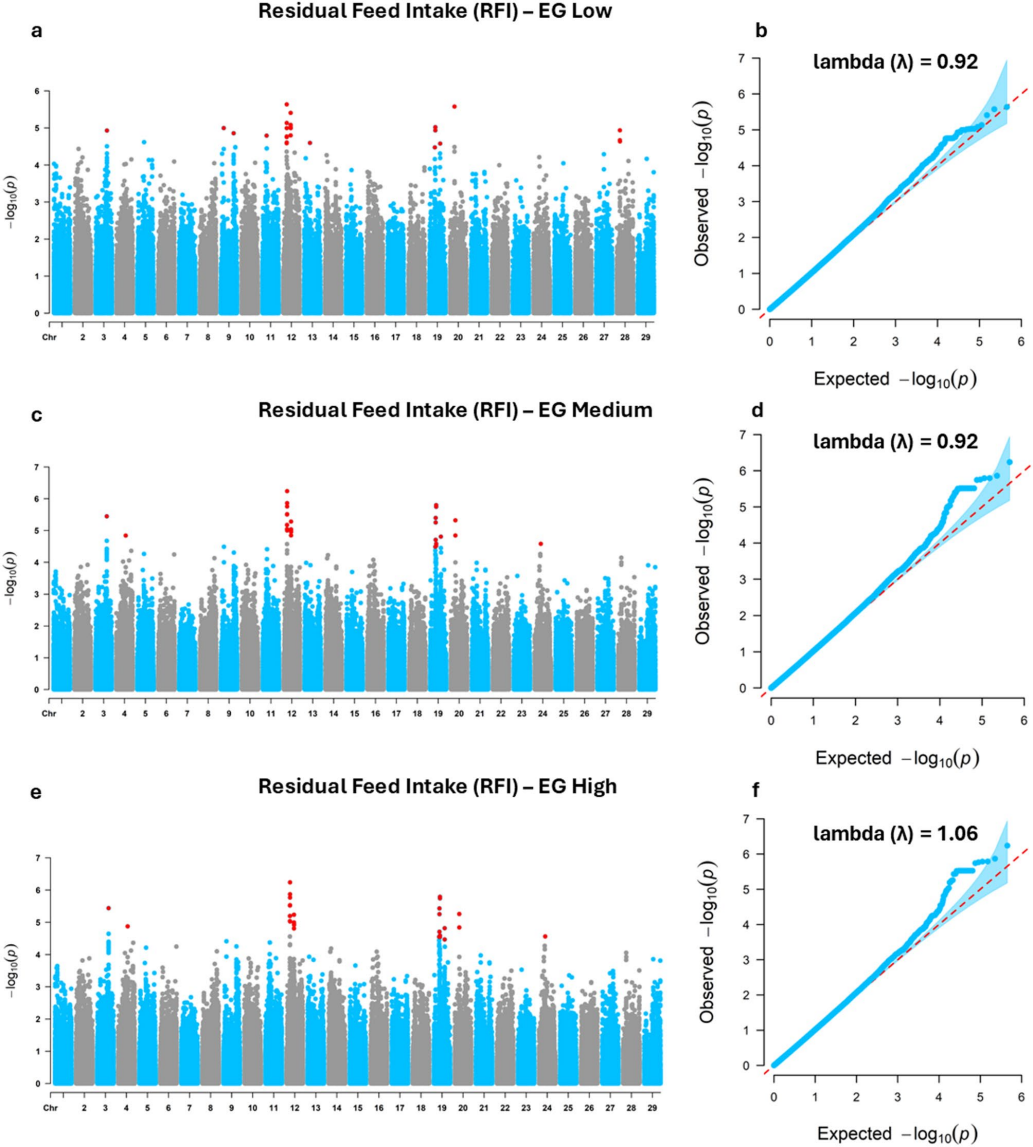
Manhattan and quantile-quantile (QQ) plots of genome-wide association study results for residual feed intake (RFI) in Nellore cattle. (**a**) Manhattan plot and (**b**) QQ plot for the Low environmental gradient (EG); (**c**) Manhattan plot and (**d**) QQ plot for the Medium EG; (**e**) Manhattan plot and (**f**) QQ plot for the High EG; Low (THI 66), Medium (THI 74), and High (THI 81).

The significant SNPs associated with feed efficiency traits were evaluated across three EG levels: low (THI 66), medium (THI 74), and high (THI 81). For RFI (Fig. 1; Table 2), 51 genome-wide significant SNPs were identified across chromosomes BTA3, BTA4, BTA9, BTA11, BTA12, BTA13, BTA19, BTA20, BTA24, and BTA28 under the three EG levels, with 37 SNPs in the low EG, 40 in the medium EG, and 41 in the high EG (Table 2). BTA12 stood out by presenting a substantial number of significant SNPs in all environments, particularly in the medium (*n* = 24) and high (*n* = 24) EGs, followed by the low EG (*n* = 22). BTA19 also showed an increased number of SNPs under more challenging environmental conditions: 5 under low EG, 11 under medium EG, and 12 under high EG.

**Table 2:** Distribution of significant single nucleotide polymorphisms (SNPs) by chromosome (BTA) across low, medium, and high environmental gradients for residual feed intake (RFI) in Nellore cattle.

Environment gradient			
Significant SNP	Low	Medium	High
BTA 3	1	1	1
BTA 4	–	1	1
BTA 9	2	–	–
BTA 11	1	–	–
BTA 12	22	24	24
BTA 13	2	–	–
BTA 19	5	11	12
BTA 20	1	2	2
BTA 24	–	1	1
BTA 28	3	–	–
Total number of significant SNPs	37	40	41

Low (THI 66), Medium (THI 74), and High (THI 81).

**Fig. 2:**
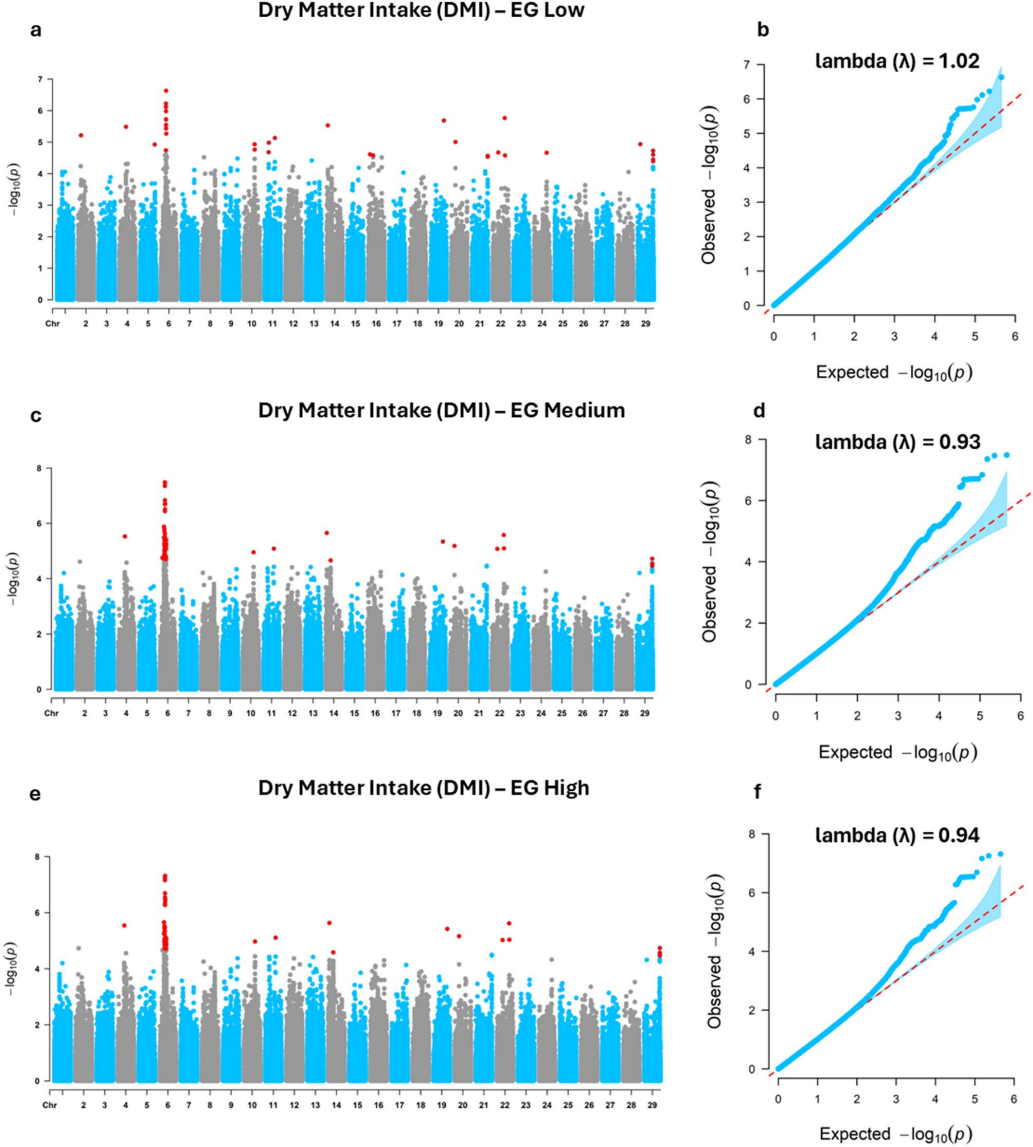
Manhattan and quantile-quantile (QQ) plots of genome-wide association study results for dry matter intake (DMI) in Nellore cattle. (**a**) Manhattan plot and (**b**) QQ plot for the Low environmental gradient (EG); (**c**) Manhattan plot and (**d**) QQ plot for the Medium EG; (**e**) Manhattan plot and (**f**) QQ plot for the High EG; Low (THI 66), Medium (THI 74), and High (THI 81).

For DMI (Fig. 2; Table 3), 136 significant SNPs were detected, distributed across chromosomes BTA2, BTA4, BTA5, BTA6, BTA10, BTA11, BTA14, BTA16, BTA19, BTA20, BTA21, BTA22, BTA24, and BTA29, with 40 SNPs identified under the low EG, 124 under the medium EG, and 85 under the high EG (Table 3). BTA6 exhibited the highest number of significant SNPs (*n* = 110) under the medium EG, followed by the high EG (*n* = 71) and the low EG (*n* = 15). Additionally, BTA10, BTA11, BTA14, and BTA29 also stood out due to changes in significant SNPs across different gradients.

**Table 3:** Distribution of significant single nucleotide polymorphisms by chromosome (BTA) across low, medium, and high environmental gradients for dry matter intake (DMI) in Nellore cattle. Low (THI 66), medium (THI 74), and high (THI 81).

Environment gradient			
Significant SNP	Low	Medium	High
BTA 2	1	–	–
BTA 4	1	1	1
BTA 5	1	–	–
BTA 6	15	110	71
BTA 10	2	1	1
BTA 11	3	1	1
BTA 14	1	2	2
BTA 16	2	–	–
BTA 19	1	1	1
BTA 20	1	1	1
BTA 21	2	–	–
BTA 22	3	3	3
BTA 24	2	–	–
BTA 29	5	4	4
Total number of significant SNPs	40	124	85

## Specific and shared distribution of significant SNPs across the EGs

**Fig. 3:**
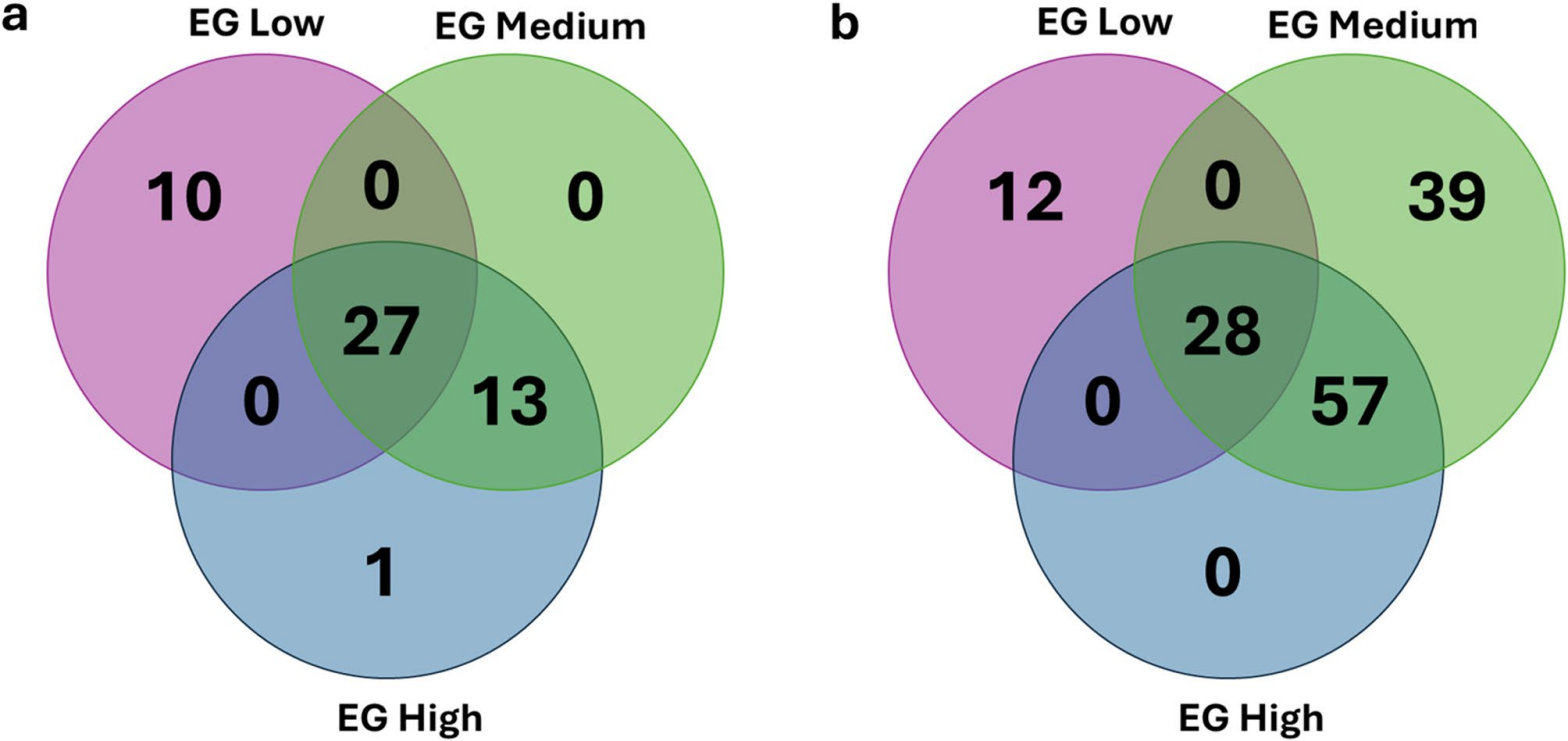
Venn diagram of significant single nucleotide polymorphisms, highlighting those that are specific and shared among the environmental gradients (EG): Low (THI 66), Medium (THI 74), and High (THI 81) for residual feed intake (RFI) (**a**) and dry matter intake (DMI) (**b**) in Nellore cattle.

The overlap of significant SNPs across the different EG for feed efficiency traits was analyzed using Venn diagrams (Fig. 3). For RFI (Fig. 3a, Supplementary Table S1), 27 SNPs were found to be shared among all three environments (low, medium, and high EG), indicating genomic regions associated with RFI regardless of environmental variation. However, a considerable number of significant SNPs were unique to specific EGs, such as 10 SNPs (BTA9: 2, BTA11: 1, BTA12: 1, BTA13: 2, BTA19: 1, and BTA28: 3) exclusive to the low EG, and 1 SNP (BTA19) exclusive to the high EG (Supplementary Table S2). Detailed information on the significant SNPs for each EG, including chromosome, position, allele frequency, proportion of additive genetic variance explained, and effects are provided in Additional File 1: Tables S3 (low EG), S4 (medium EG), and S5 (high EG).

For DMI (Fig. 3b, Supplementary Table S6), 28 SNPs were shared across all three EG levels, while 12 SNPs (BTA2: 1, BTA5: 1, BTA10: 1, BTA11: 2, BTA16: 2, BTA21: 2, BTA24: 2, and BTA29: 1) were exclusive to the lowest THI group (Supplementary Table S7). In the medium EG, 39 exclusive SNPs were identified, all located on BTA6 (Supplementary Table S7). No SNPs were exclusive to the high EG, but 57 markers identified under this condition were shared with the medium EG. Detailed information on the significant SNPs per EG, including chromosome, position, allele frequency, proportion of additive genetic variance explained, and effect are provided in Additional File 1: Supplementary Tables S8 (low EG), S9 (medium EG), and S10 (high EG).

### SNP effects across environmental gradients

**Fig. 4:**
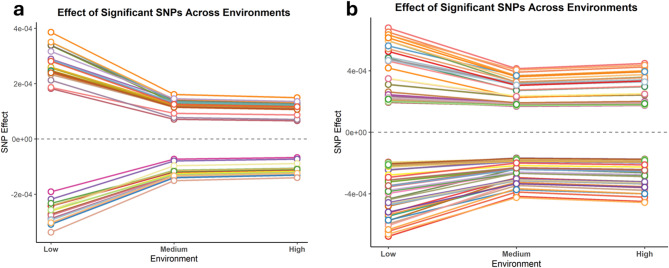
Effect of significant single nucleotide polymorphisms among the environmental gradients (EG): Low (THI 66), Medium (THI 74), and High (THI 81) for residual feed intake (RFI) (**a**) and dry matter intake (DMI) (**b**) in Nellore cattle.

Figure 4 illustrates the variation in the effects of significant SNPs across low (THI 66), medium (THI 74), and high (THI 81) EG for RFI (panel a) and DMI (panel b) in Nellore cattle. For both traits, a similar pattern is observed, a more pronounced change in SNP effects between the low and medium EGs, followed by a slight fluctuation between the medium and high EGs.

### Candidate genes identified under different thermal conditions for RFI and DMI

**Fig. 5:**
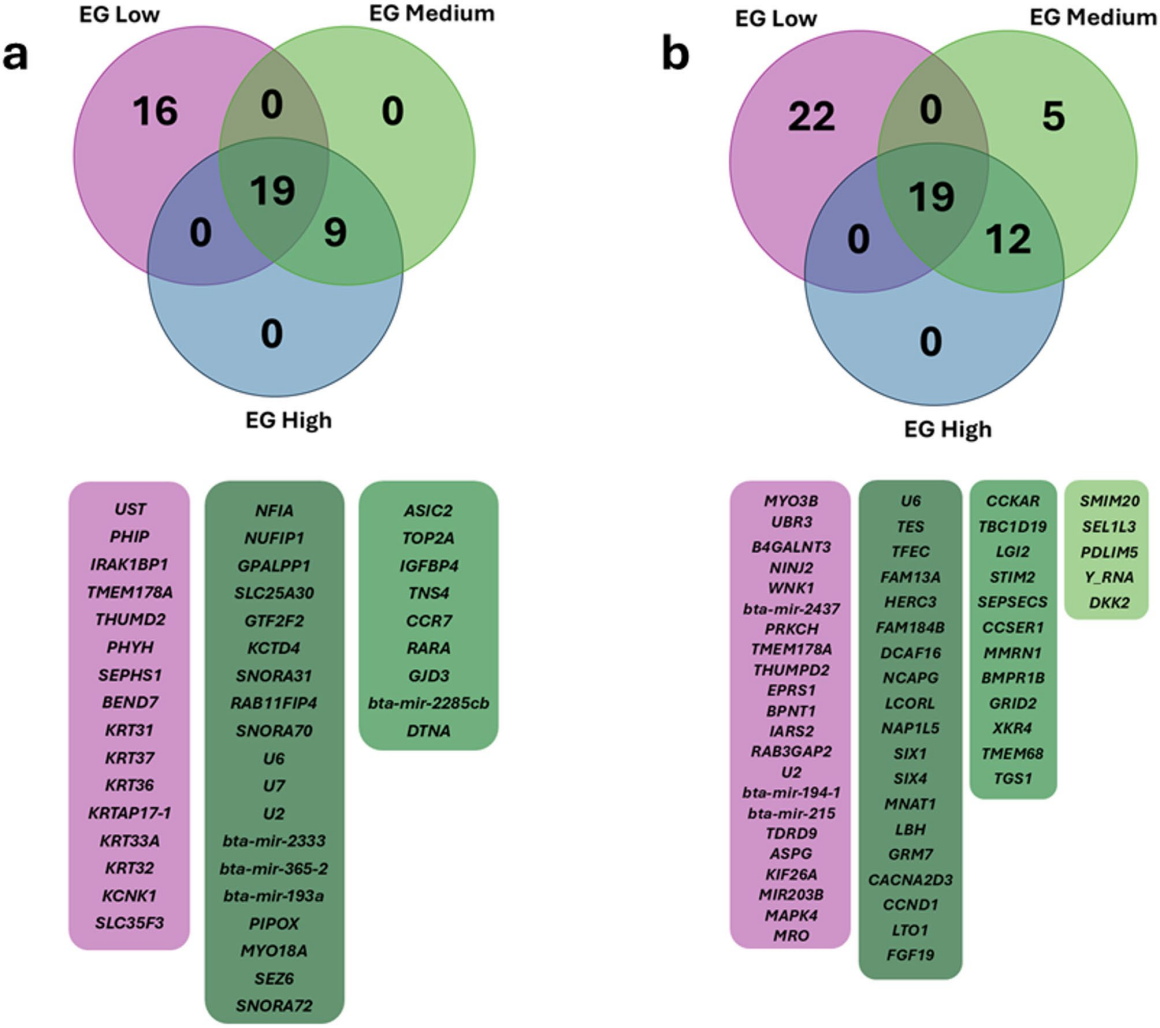
Venn diagram of genes associated with significant single nucleotide polymorphisms, highlighting those that are specific and shared among the environmental gradients (EG): Low (THI 66), Medium (THI 74), and High (THI 81) for residual feed intake (RFI) (**a**) and dry matter intake (DMI) (**b**) in Nellore cattle.

Candidate gene annotation was carried out for RFI and DMI under three distinct thermal conditions: low (THI 66), medium (THI 74), and high (THI 81). The results are illustrated in the Venn diagram presented in Fig. 5, which summarizes the exclusive and shared genes across the different EG. Additional information, including chromosomal position, associated significant SNPs, gene boundaries (start and end positions), and functional classification (gene biotype), is available in Supplementary Tables S11 (EG Low), S12 (EG Medium), and S13 (EG High) for RFI, and S14 (EG Low), S15 (EG Medium), and S16 (EG High) for DMI.

Nineteen genes were found to be commonly associated with RFI across the three EG levels, suggesting robust genetic effects independent of environmental variation (Fig. 5a). Under low heat conditions (THI < 66), 16 candidate genes were identified as specifically associated with this environment (Fig. 5a). No genes were uniquely associated with RFI in either medium (THI = 74) or high (THI = 81) heat stress environments (Fig. 5a). Nine genes were commonly identified under both moderate (THI = 74) and high (THI = 81) heat stress conditions. For DMI, a total of 58 candidate genes were annotated across the three thermal conditions (Fig. 5b), with 22 genes exclusively associated with the EG Low and 5 with the EG Medium. 12 genes were both identified in the EG Medium and EG High, while 19 genes were commonly detected across all three EG. No genes were exclusive to the High EG.

### Functional network analysis for RFI across EG

**Fig. 6:**
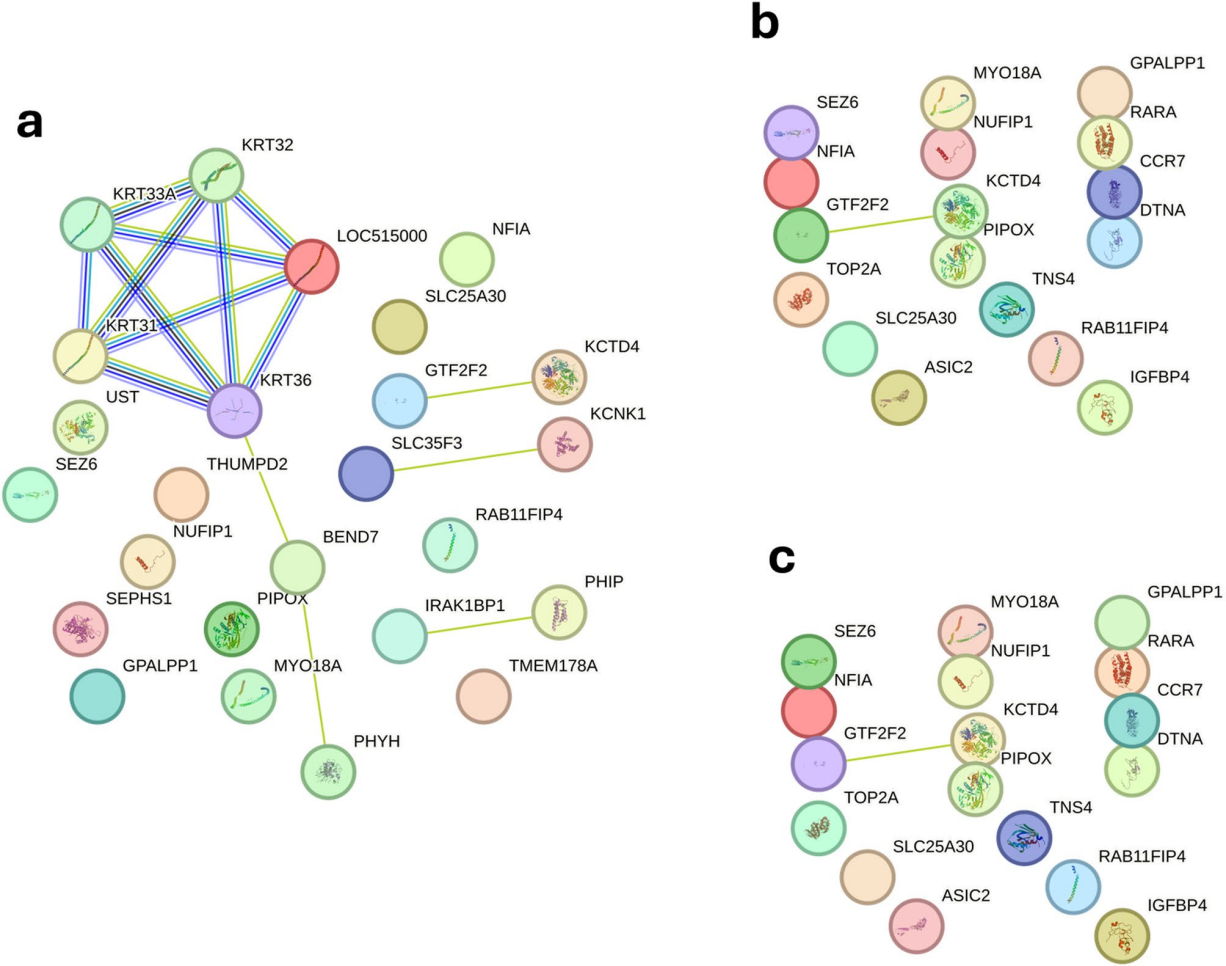
Functional network of genes mapping significant single-nucleotide polymorphisms for residual feed intake (RFI) at the low (EG = THI 66; **a**), medium (EG = THI 74; **b**) and high (EG = THI 81; **c**) environmental gradient in Nellore cattle. Each node represents a gene, while the lines connecting the nodes indicate known functional interactions or associations between these genes. The different colors of the nodes and lines indicate distinct types of interactions or classifications of biological functions, based on the network analysis.

The analysis of interaction networks of candidate genes associated with RFI in Nellore cattle in the low EG (THI = 66) displayed a high density of functional connections and the formation of well-defined clusters (Fig. 6a). A particularly prominent cluster involved members of the keratin gene family (*KRT31*, *KRT32*, *KRT33A*, *KRT36*), which exhibited strong interconnectivity. Another relevant cluster includes *BEN Domain Containing 7* (*BEND7*) and *PHYH*, which appear centrally connected in the network and are functionally associated with the keratin group. Additional co-expression relationships were observed between *SLC35F3* and *KCNK1*, and between *IRAK1BP1* and *PHIP.*

The functional network observed under moderate heat stress (THI 74) was relatively sparse and decreased density of functional modules (Fig. 6b). A central interaction involves *General Transcription Factor IIF Subunit 2* (*GTF2F2*) and *Potassium Channel Tetramerization Domain Containing 4* (*KCTD4*). The *PIPOX* gene was also located near the network center. Several new genes emerged in the network compared to low EG (THI 66), including *CCR7*, *RARA*, *Dystrobrevin Alpha* (*DTNA)*, *IGFBP4*,* Acid Sensing Ion Channel Subunit 2* (*ASIC2*), *DNA Topoisomerase II Alpha* (*TOP2A*) and *Tensin 4* (*TNS4*), most of which aggregated into small, lowly connected groups (Fig. 6b), yet suggesting functional relevance.

The functional network under high heat stress conditions (THI 81) revealed a pattern similar to that observed under moderate heat stress (Fig. 6c). Both networks exhibited sparse links, with a predominance of isolated genes or genes with few direct interactions, as well as the presence of a recurrent functional core.

### Functional network analysis for DMI across EG

**Fig. 7:**
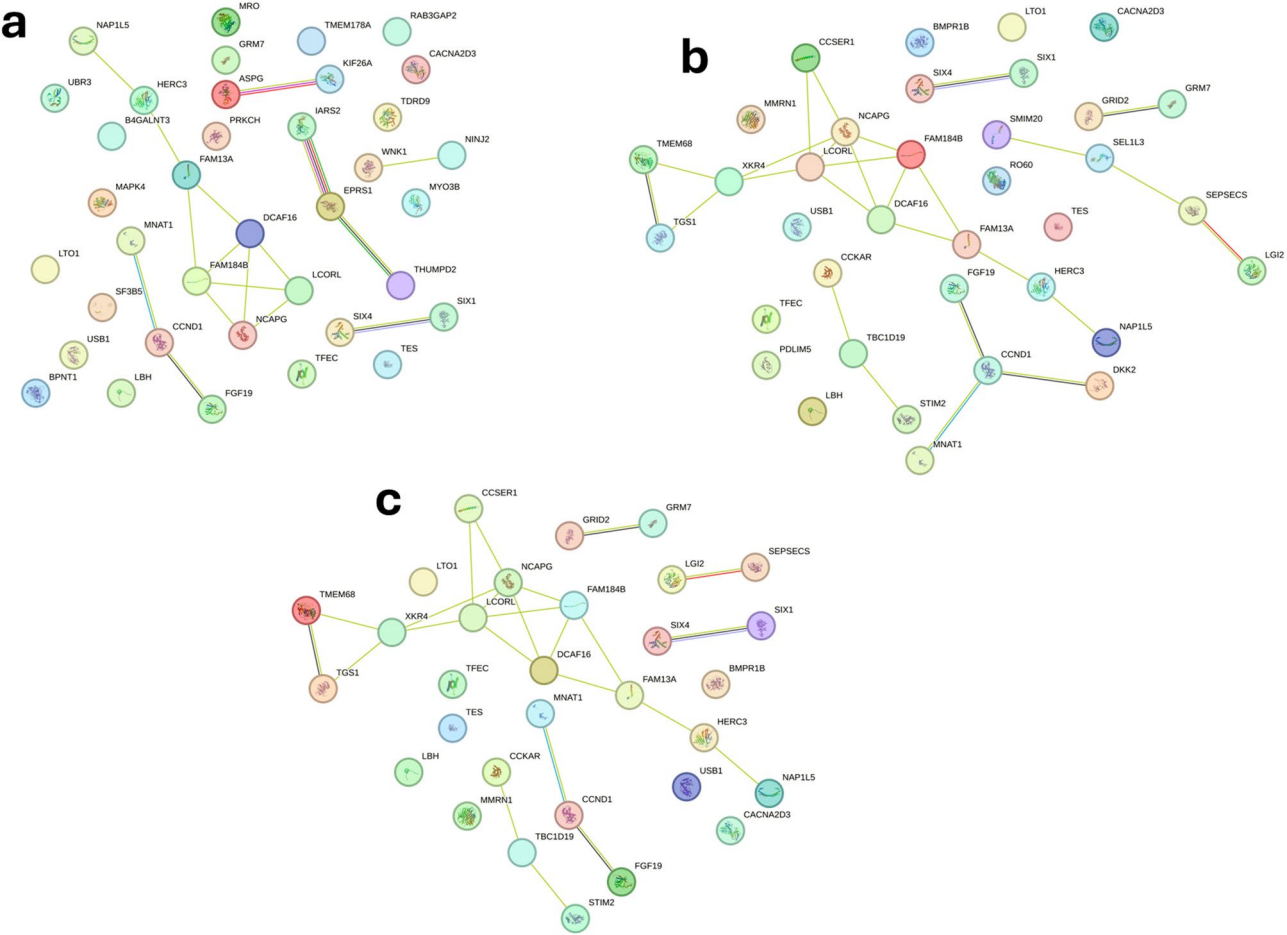
Functional network of mapping significant single nucleotide polymorphisms for dry matter intake (DMI) at the low (EG = THI 66; **a**), medium (EG = THI 74; **b**) and high (EG = THI 81; **c**) environmental gradient in Nellore cattle. Each node represents a gene, while the lines connecting the nodes indicate known functional interactions or associations between these genes. The different colors of the nodes and lines indicate distinct types of interactions or classifications of biological functions, based on the network analysis.

The analysis of interaction networks of candidate genes associated with DMI under low heat load conditions (THI = 66), exhibited high connectivity, dense formation of functional modules, and the presence of genes with central regulatory roles (Fig. 7a). Among the main interaction groups, the module composed of *Non-SMC Condensin I Complex Subunit G* (*NCAPG*), *Ligand Dependent Nuclear Receptor Corepressor Like* (*LCORL*), *Family With Sequence Similarity 184 Member B* (*FAM184B*), *DDB1 And CUL4 Associated Factor 16* (*DCAF16*), *Family With Sequence Similarity 13 Member A* (*FAM13A*), *HECT And RLD Domain Containing E3 Ubiquitin Protein Ligase 3* (*HERC3*) and *Nucleosome Assembly Protein 1 Like 5* (*NAP1L5*), stood out as the largest cluster in the network. Others relevant functional cluster is composed by the genes *Fibroblast Growth Factor 19* (*FGF19*), *Cyclin D1* (*CCND1*) and *MNAT1 Component of CDK Activating Kinase* (*MNAT1*), as well, the genes *SIX Homeobox 1* (*SIX1*) and *4* (*SIX4*), members of the SIX homeobox gene family, and the pair *ASPG*–*KIF26A*.

Under moderate heat stress (THI = 74), the network exhibits both conserved elements and notable changes in the composition and organization of the associated genes within the STRING network (Fig. 7b). The major central cluster identified under low EG, involving the genes *NCAPG*, *LCORL*, *FAM184B*, *DCAF16*, *FAM13A*, *HERC3*, and *NAP1L5*, remains present. However, the genes *XKR4*, *Coiled-Coil Serine Rich Protein 1* (*CCSER1*), *Trimethylguanosine Synthase 1* (*TGS1*) and *Transmembrane Protein 68* (*TMEM68*) emerged as interacting partners within this cluster, forming a more complex network of gene interactions. Additional gene sets formed distinct clusters, including *CCKAR*, *TBC1 Domain Family Member 19* (*TBC1D19*) and *Stromal Interaction Molecule 2* (*STIM2*) as well, the *SMIM20*, *SEL1L3*, *Sep (O-Phosphoserine) TRNA: Sec (Selenocysteine) TRNA Synthase* (*SEPSECS*), and *Leucine Rich Repeat LGI Family Member 2* (*LGI2*), which represents a connected cluster (Fig. 7b).

The comparison of functional networks associated with DMI under moderate (THI = 74) and high (THI = 81) heat stress revealed a highly similar structural organization between the two environments (Fig. 7c). Both networks exhibit strong connectivity among core genes, maintaining a functional nucleus that forms a robust and recurrent interactive axis.

### Functional genomic enrichment for RFI across EG

The functional genomic enrichment of candidate genes associated with RFI under low heat load conditions (THI 66) revealed overrepresentation of metabolic processes, particularly those related to amino acid and organic acid metabolism (Table 4). Significant GO terms included, *proteinogenic amino acid metabolic process* (GO:0170039), *L-amino acid metabolic process* (GO:0170033), *organic acid catabolic process* (GO:0016054), and *carboxylic acid catabolic process* (GO:0046395). These terms were mainly driven by *SEPHS1*, *PIPOX*, and *PHYH*.

**Table 4:** Significant gene ontology (GO) terms and Kyoto encyclopedia of genes and genomes (KEGG) pathways associated with residual feed intake (RFI) in the low environmental gradient (EG) in Nellore cattle.

GO/KEGG ID	Description	*p*-value	Gene ID
GO:0170039	Proteinogenic amino acid metabolic process	0.003	*SEPHS1*,* PIPOX*
GO:0170033	L-amino acid metabolic process	0.004	*SEPHS1*,* PIPOX*
GO:0050793	Regulation of developmental process	0.006	*PHIP*,* TMEM178A*,* SEZ6*
GO:0016054	Organic acid catabolic process	0.007	*PHYH*,* PIPOX*
GO:0046395	Carboxylic acid catabolic process	0.007	*PHYH*,* PIPOX*
GO:1901605	Alpha-amino acid metabolic process	0.008	*SEPHS1*,* PIPOX*
GO:0019752	Carboxylic acid metabolic process	0.009	*PHYH*,* SEPHS1*,* PIPOX*
GO:0043436	Oxoacid metabolic process	0.009	*PHYH*,* SEPHS1*,* PIPOX*
GO:0006082	Organic acid metabolic process	0.010	*PHYH*,* SEPHS1*,* PIPOX*
GO:0044282	Small molecule catabolic process	0.011	*PHYH*,* PIPOX*
bta04915	Estrogen signaling pathway	< 0.001	*KRT37*,* KRT36*,* KRT33A*,* KRT31*,* KRT32*
bta04146	Peroxisome	0.005	*PHYH*,* PIPOX*

At the pathway level, two KEGG pathways were significantly associated: the *estrogen signaling pathway* (bta04915) and *peroxisome pathway* (bta04146) (Table 4). The estrogen signaling pathway, involving keratin genes (*KRT31*,* KRT32*,* KRT33A*,* KRT36*,* KRT37*), while the peroxisome pathway, driven by *PHYH* and *PIPOX*. No significantly enriched biological processes and metabolic pathways were identified under medium and high heat stress conditions.

### Functional genomic enrichment for DMI in the low EG

The functional analysis of candidate genes associated with DMI in Nellore cattle under low heat load conditions (THI 66) revealed the involvement of highly organized biological processes (Table 5). Among the significantly enriched terms, *regulation of cyclin-dependent protein kinase activity* (GO:0000079; GO:1904029) and *positive regulation of cell cycle* (GO:0045787), stood out, involving the genes *CCND1* and *MNAT1*. The *mitotic cell cycle process* (GO:1903047) also showed enrichment, with the involvement of *NCAPG*.

**Table 5:** Significant gene ontology (GO) terms and Kyoto encyclopedia of genes and genomes (KEGG) pathways associated with dry matter intake (DMI) in the low environmental gradient (EG) in Nellore cattle.

GO/KEGG ID	Description	*p*-value	Gene ID
GO:0000079	Regulation of cyclin-dependent protein serine/threonine kinase activity	0.002	*CCND1*,* MNAT1*
GO:1904029	Regulation of cyclin-dependent protein kinase activity	0.002	*CCND1*,* MNAT1*
GO:0006399	tRNA metabolic process	0.002	*THUMPD2*,* IARS2*,* EPRS1*
GO:0006418	tRNA aminoacylation for protein translation	0.002	*IARS2*,* EPRS1*
GO:0043039	tRNA aminoacylation	0.002	*IARS2*,* EPRS1*
GO:0043038	Amino acid activation	0.002	*IARS2*,* EPRS1*
GO:0045787	Positive regulation of cell cycle	0.002	*CCND1*,* MNAT1*
GO:0071900	Regulation of protein serine/threonine kinase activity	0.005	*CCND1*,* MNAT1*
GO:0001932	Regulation of protein phosphorylation	0.006	*FGF19*,* CCND1*,* MNAT1*
GO:0031399	Regulation of protein modification process	0.010	*FGF19*,* CCND1*,* MNAT1*
GO:0042325	Regulation of phosphorylation	0.010	*FGF19*,* CCND1*,* MNAT1*
GO:0045859	Regulation of protein kinase activity	0.014	*CCND1*,* MNAT1*
GO:0019220	Regulation of phosphate metabolic process	0.014	*FGF19*,* CCND1*,* MNAT1*
GO:0051174	Regulation of phosphorus metabolic process	0.014	*FGF19*,* CCND1*,* MNAT1*
GO:0034660	ncRNA metabolic process	0.018	*THUMPD2*,* IARS2*,* EPRS1*
GO:0043549	Regulation of kinase activity	0.025	*CCND1*,* MNAT1*
GO:0001934	Positive regulation of protein phosphorylation	0.027	*FGF19*,* MNAT1*
GO:0051338	Regulation of transferase activity	0.029	*CCND1*,* MNAT1*
GO:0031401	Positive regulation of protein modification process	0.034	*FGF19*,* MNAT1*
GO:0006520	Amino acid metabolic process	0.035	*IARS2*,* EPRS1*
GO:0050801	Monoatomic ion homeostasis	0.035	*WNK1*,* TMEM178A*
GO:0042327	Positive regulation of phosphorylation	0.041	*FGF19*,* MNAT1*
GO:0010562	Positive regulation of phosphorus metabolic process	0.043	*FGF19*,* MNAT1*
GO:0045937	Positive regulation of phosphate metabolic process	0.043	*FGF19*,* MNAT1*
GO:1903047	Mitotic cell cycle process	0.049	*CCND1*,* NCAPG*
bta00970	Aminoacyl-tRNA biosynthesis	0.006	*IARS2*,* EPRS1*
bta04921	Oxytocin signaling pathway	0.031	*CACNA2D3*,* CCND1*
bta05202	Transcriptional misregulation in cancer	0.048	*SIX1*,* SIX4*

In parallel, biosynthetic processes were activated, including *tRNA metabolic process* (GO:0006399), *tRNA aminoacylation for protein translation* (GO:0006418), and *amino acid activation* (GO:0043038), associated with *IARS2*, *EPRS1*, and *THUMPD2* (Table 5). Protein phosphorylation regulation constituted another important functional axis, as evidenced by terms such as *regulation of phosphorylation* (GO:0042325), *regulation of protein kinase activity* (GO:0045859), *regulation of protein phosphorylation* (GO:0001932) and *positive regulation of protein phosphorylation* (GO:0001934). Genes such as *FGF19*, *CCND1*, and *MNAT1* emerged as central components within this group of processes. Other significantly enriched processes included *amino acid metabolic process* (GO:0006520), *regulation of transferase activity* (GO:0051338) and *monoatomic ion homeostasis* (GO:0050801), where *WNK1* stood out.

The KEGG pathway analysis also revealed the involvement of the *Aminoacyl-tRNA biosynthesis* (bta00970) and *Oxytocin signaling pathway* (bta04921), with genes *IARS2*, *EPRS1*,* CACNA2D3*, and *CCND1*.

### Functional genomic enrichment for DMI in the medium EG

The functional analysis of genes associated with DMI in Nellore cattle under moderate heat stress conditions (THI 74) revealed the activation of complex biological processes (Table 6). The processes *regulation of cyclin-dependent protein kinase activity* (GO:0000079; GO:1904029), *positive regulation of cell cycle* (GO:0045787), and *mitotic cell cycle process* (GO:1903047) were strongly associated with the genes *CCND1* and *MNAT1*. Notably, an enrichment of terms associated with glutamatergic synaptic transmission and trans-synaptic signaling was observed, including *synaptic transmission*,* glutamatergic* (GO:0035249), *modulation of chemical synaptic transmission* (GO:0050804), and *regulation of trans-synaptic signaling* (GO:0099177), mediated by the genes *GRM7* and *GRID2*. In addition, activation of the pathways *response to growth factor* (GO:0070848) and *cellular response to growth factor stimulus* (GO:0071363), involving the genes *BMPR1B* and *FGF19*, remain relevant in the modulation of feed intake.

**Table 6:** Significant gene ontology (GO) terms and Kyoto encyclopedia of genes and genomes (KEGG) pathways associated with dry matter intake (DMI) in the medium environmental gradient (EG) in Nellore cattle.

GO/KEGG ID	Description	*p*-value	Gene ID
GO:0000079	Regulation of cyclin-dependent protein serine/threonine kinase activity	0.002	*CCND1*,* MNAT1*
GO:1904029	Regulation of cyclin-dependent protein kinase activity	0.002	*CCND1*,* MNAT1*
GO:0035249	Synaptic transmission, glutamatergic	0.002	*GRM7*,* GRID2*
GO:0045787	Positive regulation of cell cycle	0.002	*CCND1*,* MNAT1*
GO:0071900	Regulation of protein serine/threonine kinase activity	0.005	*CCND1*,* MNAT1*
GO:0001932	Regulation of protein phosphorylation	0.006	*FGF19*,* CCND1*,* MNAT1*
GO:0031399	Regulation of protein modification process	0.009	*FGF19*,* CCND1*,* MNAT1*
GO:0050804	Modulation of chemical synaptic transmission	0.009	*GRM7*,* GRID2*
GO:0099177	Regulation of trans-synaptic signaling	0.009	*GRM7*,* GRID2*
GO:0042325	Regulation of phosphorylation	0.009	*FGF19*,* CCND1*,* MNAT1*
GO:0045859	Regulation of protein kinase activity	0.012	*CCND1*,* MNAT1*
GO:0019220	Regulation of phosphate metabolic process	0.013	*FGF19*,* CCND1*,* MNAT1*
GO:0051174	Regulation of phosphorus metabolic process	0.013	*FGF19*,* CCND1*,* MNAT1*
GO:0009451	RNA modification	0.015	*TGS1*,* SEPSECS*
GO:0043549	Regulation of kinase activity	0.023	*CCND1*,* MNAT1*
GO:0070848	Response to growth factor	0.023	*BMPR1B*,* FGF19*
GO:0071363	Cellular response to growth factor stimulus	0.023	*BMPR1B*,* FGF19*
GO:0001934	Positive regulation of protein phosphorylation	0.025	*FGF19*,* MNAT1*
GO:0051338	Regulation of transferase activity	0.027	*CCND1*,* MNAT1*
GO:0007267	Cell-cell signaling	0.030	*GRM7*,* GRID2*,* DKK2*
GO:0031401	Positive regulation of protein modification process	0.032	*FGF19*,* MNAT1*
GO:0042327	Positive regulation of phosphorylation	0.038	*FGF19*,* MNAT1*
GO:0010562	Positive regulation of phosphorus metabolic process	0.040	*FGF19*,* MNAT1*
GO:0045937	Positive regulation of phosphate metabolic process	0.040	*FGF19*,* MNAT1*
GO:1903047	Mitotic cell cycle process	0.045	*CCND1*,* NCAPG*
bta04020	Calcium signaling pathway	0.007	*CCKAR*,* FGF19*,* STIM2*
bta04080	Neuroactive ligand-receptor interaction	0.022	*CCKAR*,* GRM7*,* GRID2*
bta04921	Oxytocin signaling pathway	0.024	*CACNA2D3*,* CCND1*
bta04390	Hippo signaling pathway	0.025	*BMPR1B*,* CCND1*
bta04310	Wnt signaling pathway	0.031	*CCND1*,* DKK2*
bta04081	Hormone signaling	0.049	*BMPR1B*,* CCKAR*

Several terms related to protein phosphorylation and modification were significant, including *positive regulation of phosphorylation* (GO:0042327), *positive regulation of protein modification process* (GO:0031401), and *positive regulation of kinase activity* (GO:0045859) mainly driven by *FGF19*, *CCND1*, and *MNAT1*. Finally, *RNA modification* (GO:0009451) and *regulation of transferase activity* (GO:0051338), were linked to genes such as *TGS1*, *SEPSECS*, and *MNAT1*. The enriched KEGG pathways include critical signaling cascades such as the *Wnt signaling pathway* (bta04310), *Hippo signaling pathway* (bta04390), *Oxytocin signaling pathway* (bta04921), and *Calcium signaling pathway* (bta04020), with key contributions from *CCKAR*, *FGF19*, and *STIM2* (Table 6).

### Functional genomic enrichment for DMI in the high EG

The functional analysis of genes associated with DMI in Nellore cattle under high heat stress (THI 81) revealed a strong overrepresentation of processes related to cell cycle regulation, protein phosphorylation, and synaptic signaling, similar to the patterns observed under moderate stress, but with higher levels of statistical significance (Table 7). A distinct set of functional processes was identified that were absent under moderate stress, most notably the enrichment of terms related to synaptic signaling, such as *chemical synaptic transmission* (GO:0007268), *anterograde trans-synaptic signaling* (GO:0098916), *trans-synaptic signaling* (GO:0099537), and *synaptic signaling* (GO:0099536), mediated by the genes *GRM7* and *GRID2*. In addition, the term *regulation of cell cycle* (GO:0051726) was uniquely detected under high THI. In contrast, the *Wnt signaling pathway* (bta04310), which was present under moderate THI conditions and associated with the genes *CCND1* and *DKK2*, was no longer detected under high heat stress (Table 7).

**Table 7:** Significant gene ontology (GO) terms and Kyoto encyclopedia of genes and genomes (KEGG) pathways associated with dry matter intake (DMI) in the high environmental gradient (EG) in Nellore cattle.

GO/KEGG ID	Description	*p*-value	Gene ID
GO:0000079	Regulation of cyclin-dependent protein serine/threonine kinase activity	0.001	*CCND1*,* MNAT1*
GO:1904029	Regulation of cyclin-dependent protein kinase activity	0.001	*CCND1*,* MNAT1*
GO:0035249	Synaptic transmission, glutamatergic	0.001	*GRM7*,* GRID2*
GO:0045787	Positive regulation of cell cycle	0.002	*CCND1*,* MNAT1*
GO:0071900	Regulation of protein serine/threonine kinase activity	0.003	*CCND1*,* MNAT1*
GO:0001932	Regulation of protein phosphorylation	0.004	*FGF19*,* CCND1*,* MNAT1*
GO:0031399	Regulation of protein modification process	0.006	*FGF19*,* CCND1*,* MNAT1*
GO:0042325	Regulation of phosphorylation	0.006	*FGF19*,* CCND1*,* MNAT1*
GO:0050804	Modulation of chemical synaptic transmission	0.007	*GRM7*,* GRID2*
GO:0099177	Regulation of trans-synaptic signaling	0.007	*GRM7*,* GRID2*
GO:0019220	Regulation of phosphate metabolic process	0.009	*FGF19*,* CCND1*,* MNAT1*
GO:0051174	Regulation of phosphorus metabolic process	0.009	*FGF19*,* CCND1*,* MNAT1*
GO:0045859	Regulation of protein kinase activity	0.009	*CCND1*,* MNAT1*
GO:0009451	RNA modification	0.011	*TGS1*,* SEPSECS*
GO:0043549	Regulation of kinase activity	0.017	*CCND1*,* MNAT1*
GO:0070848	Response to growth factor	0.017	*BMPR1B*,* FGF19*
GO:0071363	Cellular response to growth factor stimulus	0.017	*BMPR1B*,* FGF19*
GO:0001934	Positive regulation of protein phosphorylation	0.019	*FGF19*,* MNAT1*
GO:0051338	Regulation of transferase activity	0.021	*CCND1*,* MNAT1*
GO:0031401	Positive regulation of protein modification process	0.024	*FGF19*,* MNAT1*
GO:0042327	Positive regulation of phosphorylation	0.029	*FGF19*,* MNAT1*
GO:0010562	Positive regulation of phosphorus metabolic process	0.031	*FGF19*,* MNAT1*
GO:0045937	Positive regulation of phosphate metabolic process	0.031	*FGF19*,* MNAT1*
GO:1903047	Mitotic cell cycle process	0.035	*CCND1*,* NCAPG*
GO:0007268	Chemical synaptic transmission	0.041	*GRM7*,* GRID2*
GO:0098916	Anterograde trans-synaptic signaling	0.041	*GRM7*,* GRID2*
GO:0099537	Trans-synaptic signaling	0.041	*GRM7*,* GRID2*
GO:0099536	Synaptic signaling	0.044	*GRM7*,* GRID2*
GO:0051726	Regulation of cell cycle	0.047	*CCND1*,* MNAT1*
bta04020	Calcium signaling pathway	0.006	*CCKAR*,* FGF19*,* STIM2*
bta04080	Neuroactive ligand-receptor interaction	0.018	*CCKAR*,* GRM7*,* GRID2*
bta04921	Oxytocin signaling pathway	0.021	*CACNA2D3*,* CCND1*
bta04390	Hippo signaling pathway	0.022	*BMPR1B*,* CCND1*
bta04081	Hormone signaling	0.043	*BMPR1B*,* CCKAR*

## Discussion

### Genomic implications of significant markers detected

The distribution of significant SNPs across different THI environments highlights the dynamic genetic regulation of feed efficiency traits in response to thermal load. BTA12 was consistently associated with RFI across all environmental conditions (Table 2), suggesting it may contain core regulatory regions influencing residual feed intake irrespective of thermal conditions. In contrast, BTA19 showed increased association under more severe heat stress, indicating potential environment-specific gene activation. This latter pattern is in line with the findings of Brunes et al.^49^, who identified a genomic window on BTA19 (42.98 to 43.76 Mb) associated with RFI in Nellore cattle, located near the significant SNP detected in the present study (41.59 Mb), thereby reinforcing the relevance of this chromosomal region for the genetic regulation of feed efficiency in beef cattle. For DMI, the prominent role of BTA6, particularly under medium and high EGs (Table 3), suggests this chromosome may harbor key regulators involved in the physiological response to increased thermal load. The detection of multiple significant SNPs on BTA6, as well as additional associations on BTA10, BTA11, BTA14, and BTA29 under high THI levels, underscores the involvement of diverse genomic regions in feed intake regulation under challenging conditions. Notably, the significant SNP identified on BTA14 in this study (22.99 Mb) overlaps with the genomic windows reported by Brunes et al.^49^ (22.29 to 22.98 Mb) and Mota et al.^50^ (22.62 to 24.71 Mb), meaning that both previous studies converge on the same BTA14 segment highlighted here, thereby strengthening the evidence that this locus plays a central role in the genetic background of DMI in Nellore cattle. The identification of genomic regions with either stable or environment-specific effects provides valuable insights for designing targeted selection strategies. Such strategies could be tailored to improve feed efficiency while simultaneously enhancing resilience to climate variability, a crucial goal for sustainable beef production in tropical regions.

### Insights into specific and shared SNPs across thermal environments

The overlap and exclusivity patterns of SNPs across the THI gradients provide insight into how thermal stress modulates the genetic architecture of feed efficiency traits (Fig. 3). For RFI, the 27 SNPs shared across all three environments likely represent core genomic regions influencing this trait regardless of heat stress level. Conversely, the SNPs exclusive to the low EG and the single SNP detected only under high EG suggest that certain loci exert environment-specific effects, which aligns with the presence of G×E interactions. For DMI, the clustering of 39 exclusive SNPs on BTA6 under medium EG reinforces the importance of this chromosome in regulating feed intake when animals are exposed to moderate thermal stress. The fact that no SNPs were exclusive to the high EG, yet many were shared between medium and high EG, suggests that as heat stress intensifies, the genetic regulation becomes more reliant on loci already active at intermediate levels of stress, rather than recruiting entirely new genomic regions. These findings highlight a nuanced genetic response to environmental challenges, where some loci are consistently important across environments, while others are “activated” only under specific thermal conditions. This dynamic profile is critical for developing genomic selection programs that aim to improve feed efficiency and resilience to climate stress, as it allows breeders to differentiate between stable and environment-sensitive genomic targets.

### Environmental modulation of SNP effects

The pattern of SNP effect variation across THI gradients (Fig. 4) suggests the presence of phenotypic plasticity, i.e., the ability of a genotype to alter its expression or effect in response to environmental changes^26,51^. The greater variation in SNP effects between the low and medium EGs may reflect a transitional environmental phase (onset of moderate heat stress) in which environmentally sensitive loci begin to modulate their activity. In contrast, the relative similarity in SNP effects between the medium and high EGs suggests the involvement of more stable loci that maintain their effects even under extreme environmental conditions. In other words, phenotypic plasticity appears to be more evident in transitional environments (THI ≈ 74) than under extreme heat stress conditions (THI ≥ 81).

The reduction in additive genetic variance under higher heat stress conditions^19^ may also be associated with the stabilization observed in genetic effects, suggesting that beyond a certain environmental threshold (THI ≈ 74), additive genetic effects become more consistent, even as environmental conditions worsen up to THI 81. This pattern may indicate a lower sensitivity to environmental fluctuations under more severe heat stress, as genetic mechanisms related to adaptation may have already been activated. This finding has important implications for genomic selection in tropical production systems, as it indicates greater genetic variability for heat adaptation under intermediate stress conditions. In such environments, the stress level is sufficient to trigger detectable adaptive responses without masking the genetic variability among animals, making it particularly useful for identifying individuals that are genetically more resilient to heat stress.

### Candidate genes identified under different thermal conditions for RFI

Among the nineteen genes found associated with RFI across the three EG levels suggest robust genetic effects independent of environmental variation (Fig. 5a). The *Nuclear Factor IA* (*NFIA*, BTA3), a transcription factor implicated in lipid metabolism and adipocyte differentiation, may influence basal energy expenditure^52,53^. *Pipecolic Acid And Sarcosine Oxidase* (*PIPOX*, BTA19) is involved in amino acid catabolism and nitrogen balance, reinforcing its relevance in maintaining cellular energetics^54^. *Myosin XVIIIA* (*MYO18A*, BTA19) is an unconventional myosin involved in maintaining myofiber integrity through cytoskeletal organization and Golgi function^55^. Disruption of *MYO18A* affects muscle morphology and intracellular trafficking, indicating its potential to influence basal energy expenditure and overall metabolic efficiency.

Under low heat load conditions (THI < 66), the genes were associated with roles in key biological processes related to feed efficiency (Fig. 5a). The detection of these genes suggests that milder thermal conditions may offer a more stable physiological baseline, reducing the environmental effects of stress-induced responses. Among these, *Pleckstrin Homology Domain Interacting Protein* (*PHIP*, BTA9), a key component of the insulin signaling through its interaction with IRS-1, may influence energy homeostasis and nutrient partitioning^56^. *Interleukin 1 Receptor Associated Kinase 1 Binding Protein 1* (*IRAK1BP1*, BTA9) modulates NF-κB signaling, and its association suggests a possible role of subclinical immune activation in energy expenditure^57^. *Transmembrane Protein 178 A* (*TMEM178A*, BTA11), related to calcium signaling^58^, and *Selenophosphate Synthetase 1* (*SEPHS1*, BTA13), involved in antioxidant defense via selenoprotein biosynthesis^59^, may contribute to cellular homeostasis and oxidative balance, both important processes for metabolic efficiency under thermoneutral conditions.

In addition to the four genes described above, other candidates such as *Phytanoyl-CoA 2-Hidroxilase* (*PHYH*, BTA13), *Potassium Two Pore Domain Channel Subfamily K Member 1* (*KCNK1*, BTA28), and *Solute Carrier Family 35 Member F3* (*SLC35F3*, BTA28) may also contribute to residual feed intake regulation under thermoneutral conditions (THI 66). *PHYH* is involved in lipid metabolism via peroxisomal oxidation^60^. Alterations in its function can affect lipid catabolism, thereby influencing basal energy expenditure and overall feed efficiency. *KCNK1* encodes a potassium channel potentially linked to energy homeostasis and cellular excitability^61,62^. Changes in its activity may indirectly impact tissue-level energy efficiency, particularly in metabolically active tissues such as skeletal muscle and liver. Finally, *SLC35F3* participates in thiamine transport, essential for mitochondrial energy production. Genetic variation in this transporter may influence feed efficiency by modulating thiamine availability and, consequently, the efficiency of carbohydrate and energy metabolism^63,64^.

The absence of genes uniquely associated with RFI in either medium (THI = 74) or high (THI = 81) heat stress environments (Fig. 5a), likely reflects the activation of shared regulatory mechanisms across stress levels. The genes commonly identified under both moderate (THI = 74) and high (THI = 81) heat stress conditions, confirming the presence of regulatory mechanisms that are consistently activated in response to thermal challenges (Fig. 5a). The recurrence of these genes across environments may indicate the involvement of biological processes associated with adaptive response to heat stress, potentially contributing to the maintenance of metabolic stability under adverse conditions. Among these genes, *Insulin Like Growth Factor Binding Protein 4* (*IGFBP4*, BTA19) regulates IGF signaling and may influence growth and metabolic adaptation^65,66^. *C-C Motif Chemokine Receptor 7* (*CCR7*, BTA19) is involved in immune cell trafficking^67^, and this may reflect the energetic cost of immune system activation during heat stress. *Retinoic Acid Receptor Alpha* (*RARA*, BTA19) a nuclear receptor associated with lipid metabolism and adipogenesis^68^, further supports the importance of metabolic regulation in feed efficiency under climatic stressful conditions.

Overall, the identification of both shared and environment-specific candidate genes associated with RFI highlights the complex interplay between genetic regulation and thermal stress. The presence of shared associations across all THI classes suggests a conserved genetic basis for feed efficiency, whereas the environment-specific signals particularly under low heat load indicate that milder conditions may enhance the detection of functionally relevant loci. These findings provide valuable insights into the genetic architecture of RFI under variable climatic scenarios and support the development of genomic selection strategies targeting both metabolic efficiency and environmental resilience. For a better understanding of the genetic mechanisms underlying RFI variation within each EG, the functions of most identified genes, as well as their interactions and potential functional implications, are presented in detail on the network patterns section.

### Candidate genes identified under different thermal environments for DMI

The findings suggest a dynamic genomic response to thermal stress, with both specific and conserved biological mechanisms regulating feed intake under varying degrees of heat stress. Nineteen genes were identified as commonly associated with DMI across all three EG, indicating the presence of constant mechanisms involved in the regulation of intake, independent of heat stress intensity (Fig. 5b). The recurrence of these genes across diverse climatic conditions suggests a stable genomic influence on feed intake that may reflect core physiological mechanisms. Among these, *NCAPG* (BTA6) has been previously associated with growth traits and feed intake in cattle^69,70^. This gene is involved in cell cycle regulation and it has been associated with growth rate and body size in several cattle breeds^71,72^. *NCAPG* influences feed intake by modulating growth demands, where larger or faster-growing animals require more feed to meet their energy needs^73^. Thus, the association of *NCAPG* with DMI occurs indirectly, mediated by physiological processes related to growth and energy homeostasis. *LCORL* (BTA6) is a transcription factor associated with skeletal growth and body size in humans, horses, and cattle^74,75^. *LCORL* has been linked to growth traits and feed efficiency in cattle, often acting in concert with *NCAPG*^76,77^. Polymorphisms in the *LCORL* gene have been associated with variability in feed intake and gain, particularly in beef cattle (Angus, Hereford, Simmental, Limousin, Charolais, Gelbvieh and Red Angus)^70^. Its role in skeletal growth may be crucial for determining body size and the corresponding feed requirements, thereby influencing DMI. The *Calcium Voltage-Gated Channel Auxiliary Subunit Alpha2delta 3* (*CACNA2D3*, BTA22) may contribute to neuroregulatory control of feeding behavior, given the importance of calcium signaling in appetite regulation and neuronal excitability^78,79^. Furthermore, *Glutamate Metabotropic Receptor 7* (*GRM7*, BTA22) may also participate in the neural regulation of feed intake through its role in synaptic signaling and behavioral responses to environmental stimuli^80^.

Under low thermal conditions (THI 66), 22 genes were uniquely associated with DMI (Fig. 5b). These genes likely reflect genetic mechanisms that are more detectable in thermoneutral conditions. Among these, *WNK Lysine Deficient Protein Kinase 1* (*WNK1*, BTA5) and *Ubiquitin Protein Ligase E3 Component N-recognin 3* (*UBR3*, BTA2), are of particular interest. *WNK1*, a kinase involved in ion transport and osmoregulation^81^, could contribute to water and electrolyte balance, a factor closely related to feed intake. *UBR3*, a component of the E3 ubiquitin ligase complex, plays a role in protein turnover and cellular quality control^82,83^. These pathways may influence metabolic efficiency and systemic adaptation under more physiologically stable conditions.

In contrast, five genes were exclusively identified under moderate heat stress conditions (THI 74), including *Small Integral Membrane Protein 20* (*SMIM20*, BTA6), *SEL1L Family Member 3* (*SEL1L3*, BTA6), *PDZ And LIM Domain 5* (*PDLIM5*, BTA6), *Ro60-Associated Y3* (*Y_RNA*, BTA6), and *Dickkopf WNT Signaling Pathway Inhibitor 2* (*DKK2*, BTA6). The limited number of unique associations observed in this EG may reflect a transitional physiological state, in which the onset of systemic stress responses begins to interfere with the genetic regulation of feed intake. However, despite their statistical association with DMI, the biological roles of these genes particularly in the context of heat stress adaptation and intake regulation in *Bos taurus indicus* cattle are not yet well characterized. Some of these genes lack direct functional links to thermal stress or metabolic processes in ruminants, underscoring the need for further functional annotation and gene expression studies to clarify their potential contributions under intermediate heat stress conditions.

Notably, no genes were exclusively associated with DMI under high heat stress conditions (Fig. 5b). This lack of specific associations may be attributed to the systemic physiological disruptions caused by high thermal stress, which can reduce the expression of genetic effects associated with DMI regulation. In such environments, the organism response is likely dominated by heat stress response pathways aimed at preserving basic cellular and metabolic stability, rather than by mechanisms adjusted to feed intake modulation^84^. Additionally, the increased phenotypic variability and reduced genetic variability under more extreme conditions may further limit the detection of environment-specific genetic signals^19^.

A subset of 12 genes was associated with DMI in medium (THI 74) and high (THI 81) EGs, suggesting the presence of biological mechanisms that are gradually involved in response to increasing thermal challenge. Among these, *Cholecystokinin A Receptor* (*CCKAR*, BTA6) is particularly noteworthy due to its established role in satiety signaling and feed intake regulation through the cholecystokinin (*CCK*) pathway^85^. The *XK Related 4* (*XKR4*, BTA14) gene encodes a protein involved in apoptosis and membrane remodeling^86,87^. *XKR4* is expressed in a wide range of tissues, including the nervous system and muscles^87^. Given that DMI influences muscle growth and energy balance, and that *XKR4* has been associated with feed intake and average daily gain^88,89^, its role in muscle-related processes may indicate functional relevance for energy metabolism under thermal stress conditions.

Overall, the identification of shared genes highlights the presence of genomic regions influencing DMI regardless of the heat stress level which the animals are being exposed to. These candidates may serve as valuable targets for future functional validation and for the development of breeding strategies aimed at improving feed efficiency across diverse environments. For a better understanding of the genetic mechanisms underlying DMI variation within each EG, the functions of most identified genes, as well as their interactions and potential functional implications, are presented in detail on the network patterns section.

### RFI network patterns in the low EG

Before describing the environment-specific networks, it is important to note that our interpretation of the STRING graphs is qualitative and depends on the subset of genes that map to significant SNP windows at each environmental gradient. The underlying protein–protein interaction evidence in STRING is fixed and does not change with THI; what changes across EGs are the significant SNPs and, consequently, the genes included in each network. Thus, when only part of a functional module remains associated in a given environment, the genes that are no longer supported by significant SNPs are simply not displayed, and the interactions that relied on them disappear from the graph. Apparent gains, losses, or fragmentation of modules across environments should therefore be interpreted as reflecting differences in the set of associated genes captured at each EG, rather than true changes in protein–protein connectivity.

Under low heat load conditions, the RFI network exhibited a clearly structured architecture dominated by a keratin cluster (Fig. 6a). These type I keratin isoforms are essential for epithelial integrity^90^, and mutations in *KRT32* disrupt immune homeostasis^91^. Although classically associated with skin, their coordinated activity may also influence epithelial renewal in metabolically relevant tissues, including the gastrointestinal epithelium. The interaction between *BEND7*, an epigenetic regulator associated with insulin metabolism^92^, and *PHYH*, involved in peroxisomal α-oxidation^93^, suggests an epigenetic-metabolic link within this cluster. Additional connections among *SLC35F3* (mitochondrial thiamine transport^63,64^, *KCNK1* (membrane excitability^62,94^, *IRAK1BP1* (Toll-like receptor-mediated inflammation^57,95^, and *PHIP* (insulin signaling and energy balance^56,96^ indicate coordinated regulation of mitochondrial metabolism, immune reactivity, and endocrine function.

In summary, the gene network associated with RFI under low thermal conditions (THI = 66) suggests that feed efficiency in this context is supported by the coordinated action of multiple biological processes. These include pathways related to epithelial integrity and tissue maintenance, metabolic activity, and epigenetic regulation. In addition, genes involved in mitochondrial function, immune signaling, and hormonal pathways reinforce the idea that more efficient animals are better able to balance energy production, inflammatory responses, and growth. Altogether, the observed network highlights the complex and integrated nature of the biological mechanisms contributing to feed efficiency, particularly under favorable environmental conditions.

### RFI network patterns in the medium EG

The functional network associated with RFI under moderate heat stress (THI = 74) became markedly less integrated (Fig. 6b). Central genes such as *GTF2F2*, involved in transcription initiation and stress-responsive inflammatory, hormonal, neurobehavioral, and epigenetic pathways^97–99^, and *KCTD4*, associated with ionic homeostasis, mitochondrial dysfunction, inflammatory signaling, and hypothalamic–pituitary axis activity^100–102^, indicate that transcriptional and intracellular signaling mechanisms gain prominence under these conditions. *PIPOX*, involved in lysine degradation and redox homeostasis^54^, remained a consistent metabolic node.

Several new genes emerged, though with limited connectivity. These included *CCR7* (T-cell migration and inflammation resolution^103–105^, *RARA* (retinoic-acid signaling and metabolic regulation^68,106–108^, *DTNA* (sarcolemmal structural integrity^109–111^, *IGFBP4* (IGF-mediated growth and adipogenesis^65,112–114^, and *ASIC2*, which links pH sensing to metabolic and autonomic control^115–117^. Their dispersed configuration suggests that, under moderate heat stress, feed efficiency depends on multiple partially coordinated biological systems rather than on the cohesive metabolic–epithelial core observed at THI 66. This pattern indicates a more heterogeneous and potentially less efficient adaptive response, requiring greater physiological plasticity to sustain energy balance.

The moderate heat stress network suggests a possible decrease in functional integration among active genes in this context. This configuration may reflect more complex adaptive challenges imposed by intermediate thermal stress. The identified interactions indicate that feed efficiency under these conditions may rely on the coordinated activity of multiple biological systems, including transcriptional regulation, energy metabolism, inflammatory signaling, and neuroendocrine control. The emergence of new genes with limited connectivity but potential functional relevance points to a more heterogeneous and possibly less efficient adaptive response. These findings suggest that, under moderate thermal stress, maintaining homeostasis and bioenergetic efficiency may require greater physiological plasticity and the activation of compensatory pathways.

### RFI network patterns in the high EG

The functional network under high heat stress conditions (THI 81, Fig. 6c) revealed to be like that observed under moderate heat stress (THI 74). The observed structural convergence suggests the existence of conserved genetic mechanisms regulating feed efficiency, regardless of the severity of thermal challenge. The persistence of pathways associated with energy metabolism, transcriptional regulation, and cellular signaling supports the hypothesis that thermal stress adaptation occurs primarily through the modulation of pre-established essential functional routes, rather than through the activation of novel gene modules. This organization underscores the polygenic complexity and multifactorial nature of the mechanisms regulating feed efficiency in thermally challenging environments, potentially reflecting a state of physiological overload, with the activation of multiple pathways in response to environmentally induced cellular damage or dysfunction, which remain consistently active even under increased heat stress.

### DMI network patterns in the low EG

Under low heat load conditions (THI = 66, Fig. 7a), the main interaction module included *NCAPG* and *LCORL*, well-established GWAS candidates for body weight and DMI^69,70,118^, together with *FAM184B* and *FAM13A*, genes involved in growth regulation, lipid metabolism, and energy homeostasis across species^119–124^. *HERC3*, an E3 ubiquitin ligase implicated in intracellular protein regulation and homeostasis^125,126^, was also part of this core. Collectively, these genes suggest coordinated control of somatic growth, metabolic efficiency, and protein turnover under favorable environmental conditions.

A second module included *FGF19*, *CCND1*, and *MNAT1*. *FGF19* plays central roles in hepatic metabolism, bile acid homeostasis, lipid regulation, and insulin-like signaling^127–129^. *CCND1* regulates the G1/S transition and cell proliferation^130–132^, while *MNAT1* is part of the CDK-activating kinase complex and is responsive to oxidative and metabolic stress^133–136^. This axis indicates tight coupling between nutrient availability, proliferative activity, and metabolic state.

Additional genes included *SIX Homeobox 1* (*SIX1*) and *4* (*SIX4*), which regulate muscle differentiation and fiber-type programming^137–139^, potentially influencing basal metabolic demand. The *Asparaginase* (*ASPG*) and *Kinesin Family Member 26 A* (*KIF26A*) pair links nitrogen metabolism^140,141^ to gastrointestinal signaling and development^142^, suggesting integration between amino acid utilization and digestive function. Overall, the network in favorable environmental conditions (THI 66) highlights strong functional connectivity among genes involved in growth, lipid metabolism, protein turnover, and muscular development, supporting a highly coordinated regulation of DMI in favorable thermal environments.

### DMI network patterns in the medium EG

Under moderate heat stress (THI = 74, Fig. 7b), the central cluster composed of *NCAPG*, *LCORL*, *FAM184B*, *DCAF16*, *FAM13A*, *HERC3*, and *NAP1L5* remained active, indicating a conserved regulatory core for growth and metabolism. However, new interacting partners emerged, including *XKR4*, *CCSER1*, *TGS1*, and *TMEM68*, suggesting remodeling of metabolic and stress-response pathways. *XKR4* has been associated with feed intake, growth, and endocrine modulation under environmental stress^89,143,144^. *TGS1* participates in RNA maturation, gluconeogenesis, and inflammatory signaling^145–147^, whereas *TMEM68* contributes to triacylglycerol synthesis and lipid homeostasis^148–150^.

Genes linked to neuroendocrine and metabolic regulation also appeared. *CCKAR*, a key receptor in satiety, gastrointestinal motility, and glucose regulation, directly affects feed intake^151–156^. *STIM2* functions as a calcium sensor and stabilizer of intracellular Ca²⁺ signaling^157–159^, connecting stress perception to metabolic adjustments. *SMIM20* encodes the precursor of the orexigenic neuropeptide Phoenixin, which stimulates feed intake and regulates appetite-related pathways^160–162^. *SEL1L3* and *SEPSECS* contribute to metabolic adaptation and oxidative stress protection through roles in energy balance and selenoprotein biosynthesis^163–168^.

In summary, the functional network for DMI under moderate heat stress (THI 74) reveals a combination of conserved regulatory cores and newly emerging components, indicating both stability and adaptation in response to thermal challenges. While central clusters involved in growth and metabolism remain active, the incorporation of new genes related to satiety signaling, energy metabolism, and cellular homeostasis suggests a dynamic remodeling of regulatory pathways. These interactions reflect the activation of neuroendocrine and metabolic mechanisms aimed at maintaining feed intake and physiological balance under stress. The presence of genes involved in appetite regulation, lipid metabolism, oxidative stress response, and calcium signaling points to a multifaceted adaptive strategy. Overall, the network suggests that under moderate thermal stress, feed intake efficiency is supported by the integration of central and peripheral signals, helping to sustain metabolic function and promote resilience in challenging environmental conditions.

### DMI network patterns in the high EG

The convergence between moderate (THI = 74) and high (THI = 81, Fig. 7c) heat stress suggests the existence of shared genetic mechanisms that consistently regulate feed intake regardless of the severity of thermal stress. The persistence of connections among genes involved in transcriptional regulation, hormonal signaling, cell proliferation, and energy metabolism supports the hypothesis that the adaptive response to extreme heat stress occurs through functional adjustments in already established pathways. Moreover, the stability of the network indicates that the genetic control of DMI in Nellore cattle is highly resilient, potentially reflecting a conserved regulatory system aimed at maintaining feed intake even under adverse environmental conditions. Collectively, these results support the hypothesis that functional coordination among DMI associated genes represents a key component of metabolic adaptation to heat stress.

### RFI enriched pathways across EG

Under low thermal conditions (THI 66), the enrichment profile for RFI indicates that feed efficiency is strongly linked to amino acid and organic acid metabolism (Table 4). The overrepresentation of pathways involved in proteinogenic and L-amino acid turnover, mainly driven by *SEPHS1*, *PIPOX* and *PHYH*, suggests that efficient nitrogen recycling and oxidative catabolism of small molecules are key mechanisms through which animals minimize residual feed intake under thermally mild conditions. This pattern is consistent with a metabolic configuration that favors precise matching between nutrient supply and energy demands.

The KEGG enrichment for the estrogen signaling pathway, involving several keratin genes, points to potential interactions between hormonal regulation, tissue turnover and metabolic efficiency. In parallel, the peroxisome pathway, driven by *PHYH* and *PIPOX*, highlights the importance of peroxisomal oxidative metabolism and lipid catabolism in shaping variation in RFI. Together, these findings suggest that, when heat load is low or absent, animals with superior feed efficiency tend to rely on coordinated control of amino acid degradation, organic acid catabolism and hormone-mediated regulation of energy metabolism.

Although the number of significant SNPs was similar across the different environmental gradients (37 in the Low EG, 40 in the Medium EG, and 41 in the High EG), significantly enriched biological processes and metabolic pathways were identified only under the low heat load conditions. This result indicates that the number of associated SNPs was not a limiting factor for the detection of functional enrichment. A possible explanation is that, under moderate and severe heat stress, the genetic background of feed efficiency becomes more diffuse, possibly due to a greater functional dispersion of the associated genes, as observed in the functional gene networks (THI 74 and THI 81). In these environments, the identified SNPs may be linked to biologically diverse functions, lacking convergence into specific pathways^169^. Furthermore, more intense heat stress may induce the activation of nonspecific or redundant genetic responses, involving multiple compensatory mechanisms that reduce the functional cohesion among the mapped genes^169^. It is also important to consider the reduction in trait heritability and the increase in residual variance^19^, factors that compromise the statistical consistency of the detected loci. Additionally, the increased environmental variability under moderate to severe heat stress may further reduce the statistical power required to identify genomic regions associated with the genetic variation in RFI.

### DMI enriched pathways in the low EG

Under low heat load (THI 66, Table 5), the functional profile associated with DMI revealed a predominantly anabolic molecular signature, suggesting that cattle express feed intake variation largely through pathways that support cellular growth and metabolic stability. The enrichment of cell-cycle regulators such as *CCND1*, *MNAT1*, and *NCAPG* indicates active tissue turnover in metabolically relevant organs, consistent with greater digestive and absorptive capacity when thermal constraints are minimal. The strong signal for tRNA metabolism and aminoacylation, driven by *IARS2*, *EPRS1*, and *THUMPD2*, points to increased translational demand and enhanced protein synthesis machinery. This pattern is compatible with animals sustaining higher rates of structural and enzymatic protein production, which may contribute to differences in feed utilization efficiency under favorable conditions.

Finally, enrichment for phosphorylation-dependent signaling, involving *FGF19*, *CCND1*, and *MNAT1*, highlights the role of intracellular signaling networks in coordinating nutrient sensing, hormonal responses, and metabolic homeostasis. Together, these pathways depict a coherent anabolic framework through which genetic variation in DMI is most effectively expressed when environmental stress is minimal. Such mechanisms may help explain why superior feed efficiency phenotypes tend to manifest more strongly under thermally mild conditions.

### DMI enriched pathways in the medium EG

Under moderate heat stress (THI 74), the functional profile associated with DMI revealed a combination of anabolic signaling and neuroendocrine regulation, indicating that feed intake at this intermediate environmental level depends on both cellular growth processes and central modulation of appetite (Table 6). As observed under low heat load, enrichment for cell-cycle regulators such as *CCND1* and *MNAT1* suggests that tissue renewal and basal anabolic activity remain important components of DMI variation even when animals experience moderate thermal challenge.

A key difference at this EG was the strong signal for glutamatergic synaptic transmission, driven by *GRM7* and *GRID2*, which points to a more prominent involvement of central neuroendocrine pathways in the control of feed intake. This enrichment is consistent with increased reliance on neural and metabolic integration when animals begin to experience thermal strain. In parallel, pathways associated with growth-factor signaling, particularly those involving *FGF19* and *BMPR1B*, indicate continued coordination between hormonal regulation, energy balance and digestive function.

The additional enrichment of phosphorylation-dependent signaling and calcium-mediated pathways (e.g., involving *CCKAR*, *FGF19* and *STIM2*) further supports the idea that feed intake under moderate heat stress is governed by a tightly regulated intracellular communication network. Together, these results suggest that, at THI 74, DMI reflects a shift from a predominantly anabolic configuration (as observed under THI 66) toward a more complex, multifactorial regulatory system, integrating cellular signaling, neuroendocrine communication and adaptive responses to environmental stress.

### DMI enriched pathways in the high EG

Under high heat stress (THI 81), the enrichment profile for DMI showed the recurrence and amplification of similar biological themes with moderate stress levels, suggesting a progressive recruitment of adaptive mechanisms in response to increasing environmental challenges (Table 7). Although cell-cycle and phosphorylation processes remained present, as previously observed under low and medium heat load, the most distinctive feature at this EG was the intensified enrichment for synaptic signaling, particularly glutamatergic and trans-synaptic communication mediated by *GRM7* and *GRID2*. This pattern indicates that, under severe thermal stress, feed intake becomes increasingly governed by central neural circuits involved in appetite regulation, behavioral modulation, and the integration of metabolic stress cues.

Another notable feature was the unique enrichment of pathways associated with cell-cycle checkpoint regulation, suggesting heightened control of mitotic progression, potentially reflecting cellular responses to oxidative stress and heat-induced damage. At the same time, the disappearance of pathways linked to Wnt signaling, which were detected under moderate heat stress, may indicate suppression of growth and cell renewal programs in favor of short-term adaptive responses that prioritize survival and systemic homeostasis. This shift, from anabolic maintenance under mild conditions to neuroendocrine compensation under severe heat load, highlights a progressive reorganization of physiological mechanisms as environmental stress intensifies.

Collectively, the results suggest that DMI regulation in Nellore cattle at high THI relies on a multilayered adaptive network, where neural signaling, hormonal coordination, and stress-response pathways become increasingly dominant as growth-related signaling is down-regulated. These findings underscore the relevance of GxE interactions for feed efficiency traits and reinforce the need to consider thermal variability when interpreting genomic mechanisms and designing breeding strategies for tropical production systems.

### Challenges and future directions

Although stratifying thermal conditions into discrete THI levels (66, 74, and 81) allowed for a structured assessment of G×E interactions, this approach presents inherent limitations. Heat stress is a dynamic and temporally variable phenomenon, often characterized by marked diurnal fluctuations in ambient temperature, relative humidity, and solar radiation. In the present study, we used average THI values and phenotypic means per feeding trial to classify environmental conditions, which, while facilitating G×E modeling, may mask short-term thermal effects on feed efficiency traits. Studies have shown that THI at specific hours of the day, particularly during peak heat, can significantly alter metabolic responses and feeding behavior^170^, with cattle typically reducing intake during hotter periods and compensating later in cooler hours. This temporal plasticity, however, is not captured when using averaged environmental and phenotypic data.

As highlighted by Silva Neto et al.^19^, future studies should prioritize the collection of longitudinal data to identify critical time windows during which heat stress exerts the greatest impact on DMI and RFI, to characterize individual adaptation patterns and feeding strategies in response to thermal stress, and to enable the modeling of phenotypic variation based on real-time environmental fluctuations rather than static period-based averages. The integration of continuous phenotypic and environmental data into G×E GWAS frameworks holds promise for improving the detection of environment-sensitive genomic regions, refining the estimation of SNP effects under variable thermal conditions, and ultimately enhancing the accuracy of genomic evaluations for selecting animals with greater resilience and metabolic stability in tropical production systems facing intensifying climate challenges.

## Conclusions

The genetic control of feed intake and feed efficiency in Nellore cattle is not only complex and polygenic but also sensitive to thermal stress conditions. The variation in genomic associations and gene network organization across different levels of heat stress reinforces the multifactorial nature of adaptation to tropical environments. In summary, the genetic networks appeared more integrated under low THI conditions, reflecting a more stable architecture when animals were not exposed to heat stress, whereas at higher THI levels additional loci became associated with the traits, suggesting that heat stress may reshape the genetic architecture by activating stress-related regions and reducing overall network integration. The identification of both environment-specific and recurrent candidate genes, together with the distinct functional patterns observed between environments, provides useful insights for refining genetic improvement strategies aimed at sustaining animal performance under increasing thermal stress. .

## Supplementary Information

Below is the link to the electronic supplementary material.

Supplementary Material 1

## Data Availability

The data analyzed in this study were obtained from the National Association of Breeders and Researchers (ANCP). The phenotypic and genotypic information was provided to the authors for academic research purposes only. The following restrictions apply: the dataset is not publicly available and its use requires formal authorization. Requests to access these datasets should be directed to Dr. João Carlos G. Giffoni Filho, President of ANCP (email: presidencia@ancp.org.br).
