# Supplementary material for: Modeling genotype-by-environment interactions across climatic conditions reveals environment-specific genomic regions and candidate genes underlying feed efficiency traits in tropical beef cattle
Source: Sci Rep. 2026 Jan 5;16:643. doi: 10.1038/s41598-025-33952-1 (PMC12779994; doi:10.1038/s41598-025-33952-1)
Supplement: Supplementary file 1 — Supplementary Material 1 [file 41598_2025_33952_MOESM1_ESM.docx]

**Table S1.** Significant single nucleotide polymorphisms shared by low, medium, and high environmental gradients (EG) for residual feed intake (RFI) in Nellore cattle.

|  |  |  | EG Low | | | EG Medium | | | EG High | | |
| --- | --- | --- | --- | --- | --- | --- | --- | --- | --- | --- | --- |
| SNP | BTA | Position (bp) | p-value | Effect | Var (%) | p-value | Effect | Var (%) | p-value | Effect | Var (%) |
| BOVINEHD0300035625 | 3 | 84,185,909 | 1.18x10^-5^ | 0.0003 | 0.0036 | 3.57x10^-6^ | 0.0001 | 0.0040 | 3.65x10^-6^ | 0.0001 | 0.0040 |
| BOVINEHD1200004514 | 12 | 15,127,015 | 2.58x10^-5^ | 0.0003 | 0.0026 | 9.09x10^-6^ | 0.0001 | 0.0030 | 9.20x10^-6^ | 0.0001 | 0.0030 |
| BOVINEHD1200004543 | 12 | 15,214,340 | 1.75x10^-5^ | -0.0003 | 0.0026 | 3.13x10^-6^ | -0.0001 | 0.0032 | 3.02x10^-6^ | -0.0001 | 0.0033 |
| BOVINEHD1200004573 | 12 | 15,301,827 | 1.71x10^-5^ | 0.0003 | 0.0026 | 3.05x10^-6^ | 0.0001 | 0.0033 | 2.95x10^-6^ | 0.0001 | 0.0033 |
| BOVINEHD1200004580 | 12 | 15,323,705 | 1.72x10^-5^ | 0.0003 | 0.0026 | 3.06x10^-6^ | 0.0001 | 0.0033 | 2.96x10^-6^ | 0.0001 | 0.0033 |
| BOVINEHD1200004583 | 12 | 15,331,134 | 1.72x10^-5^ | 0.0003 | 0.0026 | 3.06x10^-6^ | 0.0001 | 0.0033 | 2.96x10^-6^ | 0.0001 | 0.0033 |
| BOVINEHD1200004584 | 12 | 15,332,734 | 1.70x10^-5^ | -0.0003 | 0.0026 | 3.05x10^-6^ | -0.0001 | 0.0033 | 2.96x10^-6^ | -0.0001 | 0.0033 |
| BOVINEHD1200004586 | 12 | 15,338,930 | 1.72x10^-5^ | -0.0003 | 0.0026 | 3.06x10^-6^ | -0.0001 | 0.0033 | 2.96x10^-6^ | -0.0001 | 0.0033 |
| BOVINEHD1200004590 | 12 | 15,346,549 | 1.71x10^-5^ | 0.0003 | 0.0026 | 3.05x10^-6^ | 0.0001 | 0.0033 | 2.95x10^-6^ | 0.0001 | 0.0033 |
| BOVINEHD1200004594 | 12 | 15,362,724 | 2.31x10^-6^ | -0.0003 | 0.0032 | 5.77x10^-7^ | -0.0001 | 0.0038 | 5.75x10^-7^ | -0.0001 | 0.0038 |
| BOVINEHD1200004601 | 12 | 15,381,637 | 1.77x10^-5^ | 0.0003 | 0.0026 | 3.13x10^-6^ | 0.0001 | 0.0032 | 3.02x10^-6^ | 0.0001 | 0.0033 |
| BOVINEHD1200004605 | 12 | 15,385,958 | 1.71x10^-5^ | 0.0003 | 0.0026 | 3.05x10^-6^ | 0.0001 | 0.0033 | 2.95x10^-6^ | 0.0001 | 0.0033 |
| BOVINEHD1200004606 | 12 | 15,388,751 | 1.72x10^-5^ | -0.0003 | 0.0026 | 3.05x10^-6^ | -0.0001 | 0.0033 | 2.96x10^-6^ | -0.0001 | 0.0033 |
| BOVINEHD1200004607 | 12 | 15,391,191 | 1.71x10^-5^ | 0.0003 | 0.0026 | 3.06x10^-6^ | 0.0001 | 0.0033 | 2.96x10^-6^ | 0.0001 | 0.0033 |
| BOVINEHD1200004609 | 12 | 15,416,927 | 1.01x10^-5^ | -0.0003 | 0.0029 | 1.76x10^-6^ | -0.0001 | 0.0036 | 1.71x10^-6^ | -0.0001 | 0.0036 |
| BOVINEHD1200004614 | 12 | 15,421,511 | 7.35x10^-6^ | -0.0003 | 0.0030 | 1.38x10^-6^ | -0.0001 | 0.0037 | 1.35x10^-6^ | -0.0001 | 0.0037 |
| BOVINEHD1200027895 | 12 | 43,123,990 | 1.59x10^-5^ | 0.0003 | 0.0046 | 1.43x10^-5^ | 0.0001 | 0.0053 | 1.55x10^-5^ | 0.0001 | 0.0053 |
| BOVINEHD1200012140 | 12 | 43,194461 | 3.92x10^-6^ | -0.0003 | 0.0052 | 5.24x10^-6^ | -0.0001 | 0.0057 | 5.84x10^-6^ | -0.0001 | 0.0057 |
| BOVINEHD1200012145 | 12 | 43,221,777 | 9.29x10^-6^ | -0.0003 | 0.0048 | 9.83x10^-6^ | -0.0001 | 0.0054 | 1.08x10^-5^ | -0.0001 | 0.0054 |
| BOVINEHD1200012146 | 12 | 43,226,877 | 9.29x10^-6^ | -0.0003 | 0.0048 | 9.83x10^-6^ | -0.0001 | 0.0054 | 1.08x10^-5^ | -0.0001 | 0.0054 |
| BOVINEHD1200012148 | 12 | 43,256,239 | 8.27x10^-6^ | -0.0003 | 0.0048 | 9.19x10^-6^ | -0.0001 | 0.0054 | 1.01x10^-5^ | -0.0001 | 0.0054 |
| BOVINEHD1200027896 | 12 | 43,422,509 | 1.01x10^-5^ | -0.0003 | 0.0056 | 1.10x10^-5^ | -0.0001 | 0.0062 | 1.19x10^-5^ | -0.0001 | 0.0063 |
| BOVINEHD1900005359 | 19 | 18,416,730 | 3.35x10^-5^ | -0.0002 | 0.0018 | 5.61x10^-6^ | -0.0001 | 0.0022 | 5.59x10^-6^ | -0.0001 | 0.0022 |
| BOVINEHD4100014031 | 19 | 20,427,231 | 1.16x10^-5^ | -0.0002 | 0.0018 | 1.80x10^-6^ | -0.0001 | 0.0022 | 1.81x10^-6^ | -0.0001 | 0.0022 |
| BOVINEHD1900006041 | 19 | 20,443,801 | 9.49x10^-6^ | 0.0002 | 0.0018 | 1.59x10^-6^ | 0.0001 | 0.0022 | 1.62x10^-6^ | 0.0001 | 0.0022 |
| BOVINEHD4100014032 | 19 | 20,454,459 | 9.62x10^-6^ | 0.0002 | 0.0018 | 1.61x10^-6^ | 0.0001 | 0.0022 | 1.64x10^-6^ | 0.0001 | 0.0022 |
| BOVINEHD2000006348 | 20 | 21,223,114 | 2.65x10^-6^ | 0.0004 | 0.0095 | 4.79x10^-6^ | 0.0002 | 0.0090 | 5.53x10^-6^ | 0.0001 | 0.0089 |

Low (THI 66), Medium (THI 74), and High (THI 81), total additive genetic variance explained (Var %).

**Table S2.** Significant single nucleotide polymorphisms exclusive by environmental gradients (EG) for residual feed intake (RFI) in Nellore cattle.

| SNP | BTA | Position (bp) | p-value | effect | Var (%) | EG |
| --- | --- | --- | --- | --- | --- | --- |
| BOVINEHD0900005087 | 9 | 18,500,131 | 1.01x10^-5^ | -0.0002 | 0.0005 | Low |
| BOVINEHD0900024567 | 9 | 86,310,386 | 1.39x10^-5^ | 0.0002 | 0.0005 | Low |
| BOVINEHD1100006650 | 11 | 22,046,391 | 1.61x10^-5^ | 0.0003 | 0.0062 | Low |
| BOVINEHD1200004564 | 12 | 15,265,131 | 2.47x10^-5^ | 0.0003 | 0.0067 | Low |
| ARS_BFGL_NGS_109467 | 13 | 28,051,297 | 2.53x10^-5^ | 0.0004 | 0.0078 | Low |
| BOVINEHD1300008217 | 13 | 28,054,565 | 2.53x10^-5^ | 0.0004 | 0.0078 | Low |
| BOVINEHD1900012010 | 19 | 41,594,850 | 2.64x10^-5^ | 0.0004 | 0.0076 | Low |
| BOVINEHD2800001982 | 28 | 6,574,681 | 1.16x10^-5^ | -0.0002 | 0.0008 | Low |
| BOVINEHD2800001989 | 28 | 6,597,934 | 2.15x10^-5^ | 0.0002 | 0.0008 | Low |
| BOVINEHD2800002037 | 28 | 6,829,055 | 2.31x10^-5^ | -0.0003 | 0.0018 | Low |
| BOVINEHD1900011747 | 19 | 40,647,719 | 3.42x10^-5^ | 0.0001 | 0.0036 | High |

Low (THI 66) and High (THI 81), total additive genetic variance explained (Var %).

**Table S3.** Significant SNPs in the low environmental gradient (EG) associated with residual feed intake (RFI) in Nellore cattle: chromosome (BTA), position (bp), allele frequency (F), proportion of the total additive genetic variance explained (var %), and effect.

| SNP | BTA | Position (bp) | F | p-value | var (%) | effect |
| --- | --- | --- | --- | --- | --- | --- |
| BOVINEHD0300035625 | 3 | 84,185,909 | 0.7701 | 1.18x10^-5^ | 0.0036 | 0.0003 |
| BOVINEHD0900005087 | 9 | 18,500,131 | 0.0547 | 1.01x10^-5^ | 0.0005 | -0.0002 |
| BOVINEHD0900024567 | 9 | 86,310,386 | 0.9325 | 1.39x10^-5^ | 0.0005 | 0.0002 |
| BOVINEHD1100006650 | 11 | 22,046,391 | 0.6992 | 1.61x10^-5^ | 0.0062 | 0.0003 |
| BOVINEHD1200004514 | 12 | 15,127,015 | 0.8120 | 2.58x10^-5^ | 0.0026 | 0.0003 |
| BOVINEHD1200004543 | 12 | 15,214,340 | 0.1847 | 1.75x10^-5^ | 0.0026 | -0.0003 |
| BOVINEHD1200004564 | 12 | 15,265,131 | 0.6299 | 2.47x10^-5^ | 0.0067 | 0.0003 |
| BOVINEHD1200004573 | 12 | 15,301,827 | 0.8150 | 1.71x10^-5^ | 0.0026 | 0.0003 |
| BOVINEHD1200004580 | 12 | 15,323,705 | 0.8149 | 1.72x10^-5^ | 0.0026 | 0.0003 |
| BOVINEHD1200004583 | 12 | 15,331,134 | 0.8150 | 1.72x10^-5^ | 0.0026 | 0.0003 |
| BOVINEHD1200004584 | 12 | 15,332,734 | 0.1850 | 1.70x10^-5^ | 0.0026 | -0.0003 |
| BOVINEHD1200004586 | 12 | 15,338,930 | 0.1851 | 1.72x10^-5^ | 0.0026 | -0.0003 |
| BOVINEHD1200004590 | 12 | 15,346,549 | 0.8150 | 1.71x10^-5^ | 0.0026 | 0.0003 |
| BOVINEHD1200004594 | 12 | 15,362,724 | 0.1858 | 2.31x10^-6^ | 0.0032 | -0.0003 |
| BOVINEHD1200004601 | 12 | 15,381,637 | 0.8152 | 1.77x10^-5^ | 0.0026 | 0.0003 |
| BOVINEHD1200004605 | 12 | 15,385,958 | 0.8150 | 1.71x10^-5^ | 0.0026 | 0.0003 |
| BOVINEHD1200004606 | 12 | 15,388,751 | 0.1851 | 1.72x10^-5^ | 0.0026 | -0.0003 |
| BOVINEHD1200004607 | 12 | 15,391,191 | 0.8149 | 1.71x10^-5^ | 0.0026 | 0.0003 |
| BOVINEHD1200004609 | 12 | 15,416,927 | 0.1899 | 1.01x10^-5^ | 0.0029 | -0.0003 |
| BOVINEHD1200004614 | 12 | 15,421,511 | 0.1896 | 7.35x10^-6^ | 0.0030 | -0.0003 |
| BOVINEHD1200027895 | 12 | 43,123,990 | 0.3263 | 1.59x10^-5^ | 0.0046 | 0.0003 |
| BOVINEHD1200012140 | 12 | 43,194,461 | 0.6753 | 3.92x10^-6^ | 0.0052 | -0.0003 |
| BOVINEHD1200012145 | 12 | 43,221,777 | 0.6750 | 9.29x10^-6^ | 0.0048 | -0.0003 |
| BOVINEHD1200012146 | 12 | 43,226,877 | 0.6749 | 9.29x10^-6^ | 0.0048 | -0.0003 |
| BOVINEHD1200012148 | 12 | 43,256,239 | 0.6747 | 8.27x10^-6^ | 0.0048 | -0.0003 |
| BOVINEHD1200027896 | 12 | 43,422,509 | 0.6301 | 1.01x10^-5^ | 0.0056 | -0.0003 |
| ARS_BFGL_NGS_109467 | 13 | 28,051,297 | 0.4477 | 2.53x10^-5^ | 0.0078 | 0.0004 |
| BOVINEHD1300008217 | 13 | 28,054,565 | 0.4477 | 2.53x10^-5^ | 0.0078 | 0.0004 |
| BOVINEHD1900005359 | 19 | 18,416,730 | 0.1434 | 3.35x10^-5^ | 0.0018 | -0.0002 |
| BOVINEHD4100014031 | 19 | 20,427,231 | 0.1356 | 1.16x10^-5^ | 0.0018 | -0.0002 |
| BOVINEHD1900006041 | 19 | 20,443,801 | 0.8633 | 9.49x10^-6^ | 0.0018 | 0.0002 |
| BOVINEHD4100014032 | 19 | 20,454,459 | 0.8633 | 9.62x10^-6^ | 0.0018 | 0.0002 |
| BOVINEHD1900012010 | 19 | 41,594,850 | 0.5705 | 2.64x10^-5^ | 0.0076 | 0.0004 |
| BOVINEHD2000006348 | 20 | 21,223,114 | 0.5015 | 2.65x10^-6^ | 0.0095 | 0.0004 |
| BOVINEHD2800001982 | 28 | 6,574,681 | 0.9313 | 1.16x10^-5^ | 0.0008 | -0.0002 |
| BOVINEHD2800001989 | 28 | 6,597,934 | 0.0695 | 2.15x10^-5^ | 0.0008 | 0.0002 |
| BOVINEHD2800002037 | 28 | 6,829,055 | 0.1188 | 2.31x10^-5^ | 0.0018 | -0.0003 |

Low (THI 66).

**Table S4.** Significant SNPs in the medium environmental gradient (EG) associated with residual feed intake (RFI) in Nellore cattle: chromosome (BTA), position (bp), allele frequency (F), proportion of the total additive genetic variance explained (var %), and effect.

| SNP | BTA | Position (bp) | F | p-value | var (%) | effect |
| --- | --- | --- | --- | --- | --- | --- |
| BOVINEHD0300035625 | 3 | 84,185,909 | 0.7701 | 3.57x10^-6^ | 0.0040 | 0.0001 |
| BOVINEHD0400021141 | 4 | 75,826,816 | 0.3492 | 1.44x10^-5^ | 0.0063 | 0.0001 |
| BOVINEHD1200004514 | 12 | 15,127,015 | 0.8120 | 9.09x10^-6^ | 0.0030 | 0.0001 |
| BOVINEHD1200004538 | 12 | 15,203,798 | 0.8145 | 6.69x10^-6^ | 0.0030 | 0.0001 |
| BOVINEHD1200004539 | 12 | 15,206,233 | 0.8144 | 6.66x10^-6^ | 0.0030 | 0.0001 |
| BOVINEHD1200004543 | 12 | 15,214,340 | 0.1847 | 3.13x10^-6^ | 0.0032 | -0.0001 |
| BOVINEHD1200004573 | 12 | 15,301,827 | 0.8150 | 3.05x10^-6^ | 0.0033 | 0.0001 |
| BOVINEHD1200004574 | 12 | 15,303,970 | 0.1876 | 9.98x10^-6^ | 0.0030 | -0.0001 |
| BOVINEHD1200004580 | 12 | 15,323,705 | 0.8149 | 3.06x10^-6^ | 0.0033 | 0.0001 |
| BOVINEHD1200004583 | 12 | 15,331,134 | 0.8150 | 3.06x10^-6^ | 0.0033 | 0.0001 |
| BOVINEHD1200004584 | 12 | 15,332,734 | 0.1850 | 3.05x10^-6^ | 0.0033 | -0.0001 |
| BOVINEHD1200004586 | 12 | 15,338,930 | 0.1851 | 3.06x10^-6^ | 0.0033 | -0.0001 |
| BOVINEHD1200004590 | 12 | 15,346,549 | 0.8150 | 3.05x10^-6^ | 0.0033 | 0.0001 |
| BOVINEHD1200004594 | 12 | 15,362,724 | 0.1858 | 5.77x10^-7^ | 0.0038 | -0.0001 |
| BOVINEHD1200004601 | 12 | 15,381,637 | 0.8152 | 3.13x10^-6^ | 0.0032 | 0.0001 |
| BOVINEHD1200004605 | 12 | 15,385,958 | 0.8150 | 3.05x10^-6^ | 0.0033 | 0.0001 |
| BOVINEHD1200004606 | 12 | 15,388,751 | 0.1851 | 3.05x10^-6^ | 0.0033 | -0.0001 |
| BOVINEHD1200004607 | 12 | 15,391,191 | 0.8149 | 3.06x10^-6^ | 0.0033 | 0.0001 |
| BOVINEHD1200004609 | 12 | 15,416,927 | 0.1899 | 1.76x10^-6^ | 0.0036 | -0.0001 |
| BOVINEHD1200004614 | 12 | 15,421,511 | 0.1896 | 1.38x10^-6^ | 0.0037 | -0.0001 |
| BOVINEHD1200027895 | 12 | 43,123,990 | 0.3263 | 1.43x10^-5^ | 0.0053 | 0.0001 |
| BOVINEHD1200012140 | 12 | 43,194,461 | 0.6753 | 5.24x10^-6^ | 0.0057 | -0.0001 |
| BOVINEHD1200012145 | 12 | 43,221,777 | 0.6750 | 9.83x10^-6^ | 0.0054 | -0.0001 |
| BOVINEHD1200012146 | 12 | 43,226,877 | 0.6749 | 9.83x10^-6^ | 0.0054 | -0.0001 |
| BOVINEHD1200012148 | 12 | 43,256,239 | 0.6747 | 9.19x10^-6^ | 0.0054 | -0.0001 |
| BOVINEHD1200027896 | 12 | 43,422,509 | 0.6301 | 1.10x10^-5^ | 0.0062 | -0.0001 |
| BOVINEHD1900004927 | 19 | 17,119,628 | 0.2261 | 3.27x10^-5^ | 0.0041 | -0.0001 |
| BOVINEHD1900005359 | 19 | 18,416,730 | 0.1434 | 5.61x10^-6^ | 0.0022 | -0.0001 |
| BOVINEHD1900005372 | 19 | 18,457,196 | 0.1517 | 4.01x10^-6^ | 0.0025 | -0.0001 |
| BOVINEHD1900005373 | 19 | 18,458,541 | 0.8483 | 4.01x10^-6^ | 0.0025 | 0.0001 |
| BOVINEHD1900005384 | 19 | 18,495,164 | 0.8878 | 1.96x10^-5^ | 0.0012 | 0.0001 |
| BOVINEHD4100014031 | 19 | 20,427,231 | 0.1356 | 1.80x10^-6^ | 0.0022 | -0.0001 |
| BOVINEHD1900006041 | 19 | 20,443,801 | 0.8633 | 1.59x10^-6^ | 0.0022 | 0.0001 |
| ARS_BFGL_NGS_103531 | 19 | 20,448,136 | 0.8058 | 3.03x10^-5^ | 0.0031 | 0.0001 |
| BOVINEHD4100014032 | 19 | 20,454,459 | 0.8633 | 1.61x10^-6^ | 0.0022 | 0.0001 |
| ARS_BFGL_NGS_112012 | 19 | 20,472,697 | 0.8275 | 2.61x10^-5^ | 0.0026 | 0.0001 |
| BOVINEHD1900011755 | 19 | 40,686,643 | 0.7801 | 1.56x10^-5^ | 0.0044 | -0.0001 |
| BOVINEHD2000006348 | 20 | 21,223,114 | 0.5015 | 4.79x10^-6^ | 0.0090 | 0.0002 |
| BOVINEHD2000006349 | 20 | 21,223,790 | 0.5766 | 1.43x10^-5^ | 0.0077 | -0.0002 |
| BOVINEHD2400006188 | 24 | 22,422,567 | 0.4184 | 2.63x10^-5^ | 0.0071 | 0.0001 |

Medium (THI 74).

**Table S5.** Significant SNPs in the high environmental gradient (EG) associated with residual feed intake (RFI) in Nellore cattle: chromosome (BTA), position (bp), allele frequency (F), proportion of the total additive genetic variance explained (var %), and effect.

| SNP | BTA | Position (bp) | F | p-value | var (%) | effect |
| --- | --- | --- | --- | --- | --- | --- |
| BOVINEHD0300035625 | 3 | 84,185,909 | 0.7701 | 3.65x10^-6^ | 0.0040 | 0.0001 |
| BOVINEHD0400021141 | 4 | 75,826,816 | 0.3492 | 1.33x10^-5^ | 0.0064 | 0.0001 |
| BOVINEHD1200004514 | 12 | 15,127,015 | 0.8120 | 9.20x10^-6^ | 0.0030 | 0.0001 |
| BOVINEHD1200004538 | 12 | 15,203,798 | 0.8145 | 6.37x10^-6^ | 0.0031 | 0.0001 |
| BOVINEHD1200004539 | 12 | 15,206,233 | 0.8144 | 6.34x10^-6^ | 0.0031 | 0.0001 |
| BOVINEHD1200004543 | 12 | 15,214,340 | 0.1847 | 3.02x10^-6^ | 0.0033 | -0.0001 |
| BOVINEHD1200004573 | 12 | 15,301,827 | 0.8150 | 2.95x10^-6^ | 0.0033 | 0.0001 |
| BOVINEHD1200004574 | 12 | 15,303,970 | 0.1876 | 9.40x10^-6^ | 0.0030 | -0.0001 |
| BOVINEHD1200004580 | 12 | 15,323,705 | 0.8149 | 2.96x10^-6^ | 0.0033 | 0.0001 |
| BOVINEHD1200004583 | 12 | 15,331,134 | 0.8150 | 2.96x10^-6^ | 0.0033 | 0.0001 |
| BOVINEHD1200004584 | 12 | 15,332,734 | 0.1850 | 2.96x10^-6^ | 0.0033 | -0.0001 |
| BOVINEHD1200004586 | 12 | 15,338,930 | 0.1851 | 2.96x10^-6^ | 0.0033 | -0.0001 |
| BOVINEHD1200004590 | 12 | 15,346,549 | 0.8150 | 2.95x10^-6^ | 0.0033 | 0.0001 |
| BOVINEHD1200004594 | 12 | 15,362,724 | 0.1858 | 5.75x10^-7^ | 0.0038 | -0.0001 |
| BOVINEHD1200004601 | 12 | 15,381,637 | 0.8152 | 3.02x10^-6^ | 0.0033 | 0.0001 |
| BOVINEHD1200004605 | 12 | 15,385,958 | 0.8150 | 2.95x10^-6^ | 0.0033 | 0.0001 |
| BOVINEHD1200004606 | 12 | 15,388,751 | 0.1851 | 2.96x10^-6^ | 0.0033 | -0.0001 |
| BOVINEHD1200004607 | 12 | 15,391,191 | 0.8149 | 2.96x10^-6^ | 0.0033 | 0.0001 |
| BOVINEHD1200004609 | 12 | 15,416,927 | 0.1899 | 1.71x10^-6^ | 0.0036 | -0.0001 |
| BOVINEHD1200004614 | 12 | 15,421,511 | 0.1896 | 1.35x10^-6^ | 0.0037 | -0.0001 |
| BOVINEHD1200027895 | 12 | 43,123,990 | 0.3263 | 1.55x10^-5^ | 0.0053 | 0.0001 |
| BOVINEHD1200012140 | 12 | 43,194,461 | 0.6753 | 5.84x10^-6^ | 0.0057 | -0.0001 |
| BOVINEHD1200012145 | 12 | 43,221,777 | 0.6750 | 1.08x10^-5^ | 0.0054 | -0.0001 |
| BOVINEHD1200012146 | 12 | 43,226,877 | 0.6749 | 1.08x10^-5^ | 0.0054 | -0.0001 |
| BOVINEHD1200012148 | 12 | 43,256,239 | 0.6747 | 1.01x10^-5^ | 0.0054 | -0.0001 |
| BOVINEHD1200027896 | 12 | 43,422,509 | 0.6301 | 1.19x10^-5^ | 0.0063 | -0.0001 |
| BOVINEHD1900004927 | 19 | 17,119,628 | 0.2261 | 2.82x10^-5^ | 0.0041 | -0.0001 |
| BOVINEHD1900005359 | 19 | 18,416,730 | 0.1434 | 5.59x10^-6^ | 0.0022 | -0.0001 |
| BOVINEHD1900005372 | 19 | 18,457,196 | 0.1517 | 3.71x10^-6^ | 0.0025 | -0.0001 |
| BOVINEHD1900005373 | 19 | 18,458,541 | 0.8483 | 3.71x10^-6^ | 0.0025 | 0.0001 |
| BOVINEHD1900005384 | 19 | 18,495,164 | 0.8878 | 1.97x10^-5^ | 0.0012 | 0.0001 |
| BOVINEHD4100014031 | 19 | 20,427,231 | 0.1356 | 1.81x10^-6^ | 0.0022 | -0.0001 |
| BOVINEHD1900006041 | 19 | 20,443,801 | 0.8633 | 1.62x10^-6^ | 0.0022 | 0.0001 |
| ARS_BFGL_NGS_103531 | 19 | 20,448,136 | 0.8058 | 2.87x10^-5^ | 0.0031 | 0.0001 |
| BOVINEHD4100014032 | 19 | 20,454,459 | 0.8633 | 1.64x10^-6^ | 0.0022 | 0.0001 |
| ARS_BFGL_NGS_112012 | 19 | 20,472,697 | 0.8275 | 2.58x10^-5^ | 0.0026 | 0.0001 |
| BOVINEHD1900011747 | 19 | 40,647,719 | 0.2072 | 3.42x10^-5^ | 0.0036 | 0.0001 |
| BOVINEHD1900011755 | 19 | 40,686,643 | 0.7801 | 1.52x10^-5^ | 0.0044 | -0.0001 |
| BOVINEHD2000006348 | 20 | 21,223,114 | 0.5015 | 5.53x10^-6^ | 0.0089 | 0.0001 |
| BOVINEHD2000006349 | 20 | 21,223,790 | 0.5766 | 1.44x10^-5^ | 0.0077 | -0.0001 |
| BOVINEHD2400006188 | 24 | 22,422,567 | 0.4184 | 2.75x10^-5^ | 0.0071 | 0.0001 |

High (THI 81).

**Table S6.** Significant SNPs shared by low, medium, and high environmental gradients (EG) for dry matter intake (DMI) in Nellore cattle.

|  |  |  | EG Low | | | | EG Medium | | | | EG High | | |
| --- | --- | --- | --- | --- | --- | --- | --- | --- | --- | --- | --- | --- | --- |
| SNP | BTA | Position (bp) | p-value | Effect | Var (%) | p-value | | Effect | Var (%) | p-value | | Effect | Var (%) |
| BOVINEHD0400014486 | 4 | 52,213,076 | 3.27x10^-6^ | -0.0005 | 0.0024 | 2.98x10^-6^ | | -0.0003 | 0.0025 | 2.86x10^-6^ | | -0.0003 | 0.0025 |
| BOVINEHD0600010408 | 6 | 36,076,799 | 1.82x10^-5^ | -0.0004 | 0.0010 | 2.37x10^-6^ | | -0.0002 | 0.0012 | 3.42x10^-6^ | | -0.0003 | 0.0012 |
| BOVINEHD4100004494 | 6 | 36,108,228 | 7.68x10^-7^ | -0.0004 | 0.0010 | 3.27x10^-8^ | | -0.0003 | 0.0013 | 5.56x10^-8^ | | -0.0003 | 0.0013 |
| BOVINEHD0600010412 | 6 | 36,112,726 | 5.97x10^-7^ | -0.0004 | 0.0012 | 4.43x10^-8^ | | -0.0003 | 0.0014 | 6.90x10^-8^ | | -0.0003 | 0.0014 |
| BOVINEHD0600010726 | 6 | 37,301,959 | 2.83x10^-6^ | -0.0005 | 0.0021 | 3.08x10^-7^ | | -0.0003 | 0.0025 | 4.46x10^-7^ | | -0.0003 | 0.0024 |
| BOVINEHD0600010737 | 6 | 37,331,712 | 1.88x10^-6^ | 0.0005 | 0.0022 | 1.97x10^-7^ | | 0.0003 | 0.0026 | 2.87x10^-7^ | | 0.0003 | 0.0025 |
| BOVINEHD0600010739 | 6 | 37,340,937 | 1.88x10^-6^ | 0.0005 | 0.0022 | 1.97x10^-7^ | | 0.0003 | 0.0026 | 2.87x10^-7^ | | 0.0003 | 0.0025 |
| BOVINEHD0600010740 | 6 | 37,342,568 | 1.94x10^-6^ | -0.0005 | 0.0022 | 2.05x10^-7^ | | -0.0003 | 0.0026 | 2.98x10^-7^ | | -0.0003 | 0.0025 |
| BOVINEHD0600010741 | 6 | 37,345,508 | 1.91x10^-6^ | 0.0005 | 0.0022 | 1.99x10^-7^ | | 0.0003 | 0.0026 | 2.90x10^-7^ | | 0.0003 | 0.0025 |
| BOVINEHD0600010743 | 6 | 37,349,144 | 1.94x10^-6^ | -0.0005 | 0.0022 | 2.05x10^-7^ | | -0.0003 | 0.0026 | 2.98x10^-7^ | | -0.0003 | 0.0025 |
| BOVINEHD0600010746 | 6 | 37,369,380 | 1.94x10^-6^ | -0.0005 | 0.0022 | 2.05x10^-7^ | | -0.0003 | 0.0026 | 2.98x10^-7^ | | -0.0003 | 0.0025 |
| BOVINEHD4100004579 | 6 | 37,407,653 | 3.71x10^-6^ | -0.0005 | 0.0021 | 3.63x10^-7^ | | -0.0003 | 0.0025 | 5.35x10^-7^ | | -0.0003 | 0.0024 |
| HAPMAP23507_BTC_041133 | 6 | 37,412,062 | 3.67x10^-6^ | -0.0005 | 0.0021 | 3.59x10^-7^ | | -0.0003 | 0.0025 | 5.28x10^-7^ | | -0.0003 | 0.0024 |
| BOVINEHD0600010771 | 6 | 37,550,562 | 1.05x10^-6^ | 0.0005 | 0.0023 | 1.45x10^-7^ | | 0.0003 | 0.0026 | 2.04x10^-7^ | | 0.0003 | 0.0026 |
| BOVINEHD0600010840 | 6 | 37,991,751 | 2.34x10^-7^ | -0.0005 | 0.0019 | 3.42x10^-8^ | | -0.0003 | 0.0021 | 4.86x10^-8^ | | -0.0003 | 0.0021 |
| BOVINEHD0600011120 | 6 | 39,414,796 | 5.41x10^-6^ | -0.0003 | 0.0008 | 1.98x10^-7^ | | -0.0002 | 0.0011 | 3.53x10^-7^ | | -0.0003 | 0.0010 |
| BOVINEHD1000030754 | 10 | 72,865,996 | 1.18x10^-5^ | -0.0004 | 0.0014 | 1.11x10^-5^ | | -0.0003 | 0.0014 | 1.07x10^-5^ | | -0.0003 | 0.0014 |
| BOVINEHD1100019768 | 11 | 69,568,275 | 7.39x10^-6^ | 0.0005 | 0.0016 | 8.30x10^-6^ | | 0.0003 | 0.0015 | 7.78x10^-6^ | | 0.0003 | 0.0016 |
| BOVINEHD1400001642 | 14 | 5,137,561 | 2.96x10^-6^ | 0.0007 | 0.0067 | 2.23x10^-6^ | | 0.0004 | 0.0067 | 2.32x10^-6^ | | 0.0004 | 0.0067 |
| BOVINEHD1900015419 | 19 | 54,287,373 | 2.07x10^-6^ | 0.0005 | 0.0023 | 4.57x10^-6^ | | 0.0003 | 0.0020 | 3.79x10^-6^ | | 0.0003 | 0.0021 |
| BOVINEHD2000006349 | 20 | 21,223,790 | 9.89x10^-6^ | -0.0007 | 0.0083 | 6.56x10^-6^ | | -0.0004 | 0.0085 | 6.88x10^-6^ | | -0.0005 | 0.0085 |
| BOVINEHD2200005412 | 22 | 18,710,302 | 2.13x10^-5^ | 0.0006 | 0.0075 | 8.41x10^-6^ | | 0.0004 | 0.0083 | 9.42x10^-6^ | | 0.0004 | 0.0082 |
| BOVINEHD2200013355 | 22 | 45,863,244 | 1.72x10^-6^ | 0.0006 | 0.0038 | 2.66x10^-6^ | | 0.0003 | 0.0034 | 2.41x10^-6^ | | 0.0004 | 0.0035 |
| BOVINEHD2200013649 | 22 | 46,897,299 | 2.65x10^-5^ | -0.0007 | 0.0081 | 8.07x10^-6^ | | -0.0004 | 0.0090 | 9.18x10^-6^ | | -0.0005 | 0.0089 |
| ARS_BFGL_NGS_14050 | 29 | 46,844,718 | 1.88x10^-5^ | -0.0005 | 0.0032 | 1.91x10^-5^ | | -0.0003 | 0.0031 | 1.82x10^-5^ | | -0.0004 | 0.0032 |
| BOVINEHD2900014052 | 29 | 46,847,971 | 2.52x10^-5^ | 0.0006 | 0.0048 | 2.83x10^-5^ | | 0.0004 | 0.0045 | 2.67x10^-5^ | | 0.0004 | 0.0045 |
| BOVINEHD2900015606 | 29 | 46,852,455 | 4.05x10^-5^ | 0.0005 | 0.0033 | 3.54x10^-5^ | | 0.0003 | 0.0032 | 3.40x10^-5^ | | 0.0004 | 0.0033 |
| BOVINEHD2900014059 | 29 | 46,865,487 | 3.57x10^-5^ | -0.0005 | 0.0032 | 2.98x10^-5^ | | -0.0003 | 0.0031 | 2.91x10^-5^ | | -0.0004 | 0.0032 |

Low (THI 66), Medium (THI 74), and High (THI 81), total additive genetic variance explained (Var %).

**Table S7.** Significant SNPs exclusive by environmental gradients (EG) for dry matter intake (DMI) in Nellore cattle.

| SNP | BTA | Position (bp) | p-value | effect | Var (%) | EG |
| --- | --- | --- | --- | --- | --- | --- |
| BOVINEHD0200007602 | 2 | 26,215,473 | 6.10x10^-6^ | -0.0004 | 0.0010 | Low |
| BOVINEHD0500031113 | 5 | 107,490,755 | 1.19x10^-5^ | -0.0006 | 0.0039 | Low |
| BOVINEHD1000021008 | 10 | 73,565,810 | 1.72x10^-5^ | 0.0006 | 0.0044 | Low |
| BOVINEHD1100006648 | 11 | 22,044,673 | 2.09x10^-5^ | -0.0006 | 0.0060 | Low |
| BOVINEHD1100007078 | 11 | 23,479,423 | 1.04x10^-5^ | -0.0006 | 0.0048 | Low |
| ARS_BFGL_NGS_68742 | 16 | 10,823,924 | 2.44x10^-5^ | 0.0005 | 0.0039 | Low |
| BOVINEHD1600006703 | 16 | 23,562,164 | 2.64x10^-5^ | -0.0005 | 0.0036 | Low |
| BOVINEHD2100020541 | 21 | 68,625,952 | 2.91x10^-5^ | -0.0006 | 0.0076 | Low |
| BOVINEHD2100020549 | 21 | 68,656,870 | 2.73x10^-5^ | 0.0007 | 0.0078 | Low |
| BOVINEHD2400014207 | 24 | 50,287,687 | 2.18x10^-5^ | -0.0006 | 0.0071 | Low |
| BOVINEHD2400014208 | 24 | 50,288,942 | 2.18x10^-5^ | 0.0006 | 0.0071 | Low |
| BOVINEHD2900002282 | 29 | 8,080,028 | 1.16x10^-5^ | 0.0004 | 0.0011 | Low |
| BOVINEHD0600005354 | 6 | 18,042,182 | 1.78x10^-5^ | -0.0003 | 0.0023 | Medium |
| BTA_121735_NO_RS | 6 | 30,037,311 | 1.45x10^-5^ | -0.0002 | 0.0004 | Medium |
| HAPMAP59443_RS29009843 | 6 | 30,059,483 | 1.75x10^-5^ | -0.0002 | 0.0005 | Medium |
| BOVINEHD0600009530 | 6 | 32,611,538 | 1.36x10^-5^ | 0.0002 | 0.0005 | Medium |
| BOVINEHD0600009626 | 6 | 33,083,911 | 1.54x10^-5^ | 0.0002 | 0.0006 | Medium |
| BOVINEHD0600009627 | 6 | 33,089,632 | 1.43x10^-5^ | -0.0002 | 0.0006 | Medium |
| BOVINEHD0600009817 | 6 | 33,825,529 | 1.19x10^-5^ | -0.0002 | 0.0006 | Medium |
| BOVINEHD0600009821 | 6 | 33,837,535 | 1.52x10^-5^ | -0.0002 | 0.0006 | Medium |
| BOVINEHD0600009851 | 6 | 33,974,052 | 1.75x10^-5^ | 0.0002 | 0.0006 | Medium |
| BOVINEHD0600009854 | 6 | 33,991,326 | 1.89x10^-5^ | 0.0002 | 0.0006 | Medium |
| BOVINEHD0600009855 | 6 | 33,991,862 | 1.95x10^-5^ | 0.0002 | 0.0006 | Medium |
| BOVINEHD0600009858 | 6 | 34,006,110 | 1.85x10^-5^ | 0.0002 | 0.0006 | Medium |
| BOVINEHD0600009859 | 6 | 34,007,025 | 1.92x10^-5^ | 0.0002 | 0.0006 | Medium |
| BOVINEHD0600009860 | 6 | 34,007,974 | 1.92x10^-5^ | -0.0002 | 0.0006 | Medium |
| BOVINEHD0600009861 | 6 | 34,008,851 | 1.92x10^-5^ | -0.0002 | 0.0006 | Medium |
| BOVINEHD0600009862 | 6 | 34,011,629 | 1.92x10^-5^ | -0.0002 | 0.0006 | Medium |
| BOVINEHD0600009863 | 6 | 34,012,470 | 1.95x10^-5^ | 0.0002 | 0.0006 | Medium |
| BOVINEHD0600009864 | 6 | 34,015,505 | 1.95x10^-5^ | 0.0002 | 0.0006 | Medium |
| BOVINEHD0600009865 | 6 | 34,016,254 | 1.92x10^-5^ | -0.0002 | 0.0006 | Medium |
| HAPMAP27887_BTA_156628 | 6 | 34,018,647 | 1.92x10^-5^ | -0.0002 | 0.0006 | Medium |
| BOVINEHD0600009866 | 6 | 34,020,118 | 1.95x10^-5^ | 0.0002 | 0.0006 | Medium |
| BOVINEHD0600009867 | 6 | 34,021,322 | 1.89x10^-5^ | -0.0002 | 0.0006 | Medium |
| BOVINEHD0600009868 | 6 | 34,021,807 | 1.92x10^-5^ | 0.0002 | 0.0006 | Medium |
| BOVINEHD0600009870 | 6 | 34,023,559 | 1.86x10^-5^ | -0.0002 | 0.0006 | Medium |
| BOVINEHD0600009871 | 6 | 34,024,620 | 1.92x10^-5^ | -0.0002 | 0.0006 | Medium |
| BOVINEHD0600009872 | 6 | 34,025,427 | 1.92x10^-5^ | -0.0002 | 0.0006 | Medium |
| BOVINEHD0600009873 | 6 | 34,027,236 | 1.92x10^-5^ | -0.0002 | 0.0006 | Medium |
| BOVINEHD0600009874 | 6 | 34,030,066 | 1.95x10^-5^ | 0.0002 | 0.0006 | Medium |
| BOVINEHD0600009876 | 6 | 34,036,502 | 1.49x10^-5^ | -0.0002 | 0.0006 | Medium |
| BOVINEHD0600009878 | 6 | 34,038,779 | 1.49x10^-5^ | 0.0002 | 0.0006 | Medium |
| BOVINEHD0600009994 | 6 | 34,461,838 | 1.32x10^-5^ | -0.0002 | 0.0011 | Medium |
| BOVINEHD0600010025 | 6 | 34,574,321 | 1.07x10^-5^ | -0.0002 | 0.0006 | Medium |
| BOVINEHD0600010407 | 6 | 36,072,095 | 1.92x10^-5^ | -0.0002 | 0.0013 | Medium |
| BOVINEHD0600010409 | 6 | 36,086,492 | 1.92x10^-5^ | 0.0002 | 0.0013 | Medium |
| BOVINEHD4100004620 | 6 | 37,929,882 | 1.85x10^-5^ | -0.0003 | 0.0044 | Medium |
| BOVINEHD0600012810 | 6 | 45,341,908 | 1.99x10^-5^ | -0.0004 | 0.0070 | Medium |
| BOVINEHD0600013047 | 6 | 46,126,112 | 1.63x10^-5^ | -0.0002 | 0.0005 | Medium |
| BOVINEHD0600013048 | 6 | 46,126,660 | 1.66x10^-5^ | 0.0002 | 0.0005 | Medium |
| BOVINEHD0600013049 | 6 | 46,129,399 | 1.65x10^-5^ | -0.0002 | 0.0005 | Medium |

Low (THI 66) and Medium (THI 74), total additive genetic variance explained (Var %).

**Table S8.** Significant SNPs in the low environmental gradient (EG) associated with dry matter intake (DMI) in Nellore cattle: chromosome (BTA), position (bp), allele frequency (F), proportion of the total additive genetic variance explained (var %), and effect.

| SNP | BTA | Position (bp) | F | p-value | var (%) | effect |
| --- | --- | --- | --- | --- | --- | --- |
| BOVINEHD0200007602 | 2 | 26,215,473 | 0.9230 | 6.10x10^-6^ | 0.0010 | -0.0004 |
| BOVINEHD0400014486 | 4 | 52,213,076 | 0.8314 | 3.27x10^-6^ | 0.0024 | -0.0005 |
| BOVINEHD0500031113 | 5 | 107,490,755 | 0.2003 | 1.19x10^-5^ | 0.0039 | -0.0006 |
| BOVINEHD0600010408 | 6 | 36,076,799 | 0.8795 | 1.82x10^-5^ | 0.0010 | -0.0004 |
| BOVINEHD4100004494 | 6 | 36,108,228 | 0.8848 | 7.68x10^-7^ | 0.0010 | -0.0004 |
| BOVINEHD0600010412 | 6 | 36,112,726 | 0.8809 | 5.97x10^-7^ | 0.0012 | -0.0004 |
| BOVINEHD0600010726 | 6 | 37,301,959 | 0.8425 | 2.83x10^-6^ | 0.0021 | -0.0005 |
| BOVINEHD0600010737 | 6 | 37,331,712 | 0.1570 | 1.88x10^-6^ | 0.0022 | 0.0005 |
| BOVINEHD0600010739 | 6 | 37,340,937 | 0.1570 | 1.88x10^-6^ | 0.0022 | 0.0005 |
| BOVINEHD0600010740 | 6 | 37,342,568 | 0.8431 | 1.94x10^-6^ | 0.0022 | -0.0005 |
| BOVINEHD0600010741 | 6 | 37,345,508 | 0.1570 | 1.91x10^-6^ | 0.0022 | 0.0005 |
| BOVINEHD0600010743 | 6 | 37,349,144 | 0.8431 | 1.94x10^-6^ | 0.0022 | -0.0005 |
| BOVINEHD0600010746 | 6 | 37,369,380 | 0.8431 | 1.94x10^-6^ | 0.0022 | -0.0005 |
| BOVINEHD4100004579 | 6 | 37,407,653 | 0.8428 | 3.71x10^-6^ | 0.0021 | -0.0005 |
| HAPMAP23507_BTC_041133 | 6 | 37,412,062 | 0.8428 | 3.67x10^-6^ | 0.0021 | -0.0005 |
| BOVINEHD0600010771 | 6 | 37,550,562 | 0.1578 | 1.05x10^-6^ | 0.0023 | 0.0005 |
| BOVINEHD0600010840 | 6 | 37,991,751 | 0.8644 | 2.34x10^-7^ | 0.0019 | -0.0005 |
| BOVINEHD0600011120 | 6 | 39,414,796 | 0.8952 | 5.41x10^-6^ | 0.0008 | -0.0003 |
| BOVINEHD1000030754 | 10 | 72,865,996 | 0.8914 | 1.18x10^-5^ | 0.0014 | -0.0004 |
| BOVINEHD1000021008 | 10 | 73,565,810 | 0.7797 | 1.72x10^-5^ | 0.0044 | 0.0006 |
| BOVINEHD1100006648 | 11 | 22,044,673 | 0.6296 | 2.09x10^-5^ | 0.0060 | -0.0006 |
| BOVINEHD1100007078 | 11 | 23,479,423 | 0.2311 | 1.04x10^-5^ | 0.0048 | -0.0006 |
| BOVINEHD1100019768 | 11 | 69,568,275 | 0.1165 | 7.39x10^-6^ | 0.0016 | 0.0005 |
| BOVINEHD1400001642 | 14 | 5,137,561 | 0.7288 | 2.96x10^-6^ | 0.0067 | 0.0007 |
| ARS_BFGL_NGS_68742 | 16 | 10,823,924 | 0.3465 | 2.44x10^-5^ | 0.0039 | 0.0005 |
| BOVINEHD1600006703 | 16 | 23,562,164 | 0.2280 | 2.64x10^-5^ | 0.0036 | -0.0005 |
| BOVINEHD1900015419 | 19 | 54,287,373 | 0.8741 | 2.07x10^-6^ | 0.0023 | 0.0005 |
| BOVINEHD2000006349 | 20 | 21,223,790 | 0.5766 | 9.89x10^-6^ | 0.0083 | -0.0007 |
| BOVINEHD2100020541 | 21 | 68,625,952 | 0.4665 | 2.91x10^-5^ | 0.0076 | -0.0006 |
| BOVINEHD2100020549 | 21 | 68,656,870 | 0.5448 | 2.73x10^-5^ | 0.0078 | 0.0007 |
| BOVINEHD2200005412 | 22 | 18,710,302 | 0.4694 | 2.13x10^-5^ | 0.0075 | 0.0006 |
| BOVINEHD2200013355 | 22 | 45,863,244 | 0.1750 | 1.72x10^-6^ | 0.0038 | 0.0006 |
| BOVINEHD2200013649 | 22 | 46,897,299 | 0.4994 | 2.65x10^-5^ | 0.0081 | -0.0007 |
| BOVINEHD2400014207 | 24 | 50,287,687 | 0.3899 | 2.18x10^-5^ | 0.0071 | -0.0006 |
| BOVINEHD2400014208 | 24 | 50,288,942 | 0.6101 | 2.18x10^-5^ | 0.0071 | 0.0006 |
| BOVINEHD2900002282 | 29 | 8,080,028 | 0.9100 | 1.16x10^-5^ | 0.0011 | 0.0004 |
| ARS_BFGL_NGS_14050 | 29 | 46,844,718 | 0.8202 | 1.88x10^-5^ | 0.0032 | -0.0005 |
| BOVINEHD2900014052 | 29 | 46,847,971 | 0.2248 | 2.52x10^-5^ | 0.0048 | 0.0006 |
| BOVINEHD2900015606 | 29 | 46,852,455 | 0.1895 | 4.05x10^-5^ | 0.0033 | 0.0005 |
| BOVINEHD2900014059 | 29 | 46,865,487 | 0.8154 | 3.57x10^-5^ | 0.0032 | -0.0005 |

Low (THI 66).

**Table S9.** Significant SNPs in the medium environmental gradient (EG) associated with dry matter intake (DMI) in Nellore cattle: chromosome (BTA), position (bp), allele frequency (F), proportion of the total additive genetic variance explained (var %), and effect.

| SNP | BTA | Position (bp) | F | p-value | var (%) | effect |
| --- | --- | --- | --- | --- | --- | --- |
| BOVINEHD0400014486 | 4 | 52,213,076 | 0.8314 | 2.98x10^-6^ | 0.0025 | -0.0003 |
| BOVINEHD0600005354 | 6 | 18,042,182 | 0.1411 | 1.78x10^-5^ | 0.0023 | -0.0003 |
| BOVINEHD0600008722 | 6 | 29,684,500 | 0.9111 | 3.31x10^-6^ | 0.0005 | -0.0002 |
| HAPMAP54442_RS29025673 | 6 | 29,700,842 | 0.0892 | 1.32x10^-6^ | 0.0006 | 0.0002 |
| BTA_121735_NO_RS | 6 | 30,037,311 | 0.9111 | 1.45x10^-5^ | 0.0004 | -0.0002 |
| HAPMAP59443_RS29009843 | 6 | 30,059,483 | 0.9059 | 1.75x10^-5^ | 0.0005 | -0.0002 |
| HAPMAP32447_BTC_033214 | 6 | 32,254,947 | 0.9019 | 5.45x10^-6^ | 0.0006 | -0.0002 |
| BOVINEHD0600009439 | 6 | 32,339,668 | 0.0989 | 9.62x10^-6^ | 0.0006 | 0.0002 |
| BOVINEHD0600009468 | 6 | 32,453,198 | 0.0980 | 3.17x10^-6^ | 0.0006 | 0.0002 |
| BOVINEHD0600009469 | 6 | 32,456964 | 0.0974 | 1.92x10^-6^ | 0.0006 | 0.0002 |
| BOVINEHD0600009470 | 6 | 32,461,586 | 0.9027 | 1.60x10^-6^ | 0.0006 | -0.0002 |
| BOVINEHD0600009471 | 6 | 32,462,575 | 0.0974 | 1.92x10^-6^ | 0.0006 | 0.0002 |
| BOVINEHD0600009483 | 6 | 32,499,982 | 0.0974 | 1.61x10^-6^ | 0.0006 | 0.0002 |
| BOVINEHD0600009530 | 6 | 32,611,538 | 0.0966 | 1.36x10^-5^ | 0.0005 | 0.0002 |
| BOVINEHD0600009555 | 6 | 32,763,336 | 0.9032 | 5.96x10^-6^ | 0.0005 | -0.0002 |
| BOVINEHD0600009626 | 6 | 33,083,911 | 0.1093 | 1.54x10^-5^ | 0.0006 | 0.0002 |
| BOVINEHD0600009627 | 6 | 33,089,632 | 0.8910 | 1.43x10^-5^ | 0.0006 | -0.0002 |
| BOVINEHD0600009802 | 6 | 33,747,292 | 0.8678 | 6.14x10^-6^ | 0.0012 | -0.0002 |
| BOVINEHD0600009803 | 6 | 33,747,860 | 0.1322 | 6.15x10^-6^ | 0.0012 | 0.0002 |
| BOVINEHD0600009804 | 6 | 33,752,201 | 0.8675 | 3.64x10^-6^ | 0.0013 | -0.0002 |
| BOVINEHD0600034663 | 6 | 33,788,813 | 0.8483 | 3.17x10^-6^ | 0.0018 | -0.0003 |
| BOVINEHD0600009817 | 6 | 33,825,529 | 0.8913 | 1.19x10^-5^ | 0.0006 | -0.0002 |
| BOVINEHD0600009821 | 6 | 33,837,535 | 0.8916 | 1.52x10^-5^ | 0.0006 | -0.0002 |
| BOVINEHD0600009851 | 6 | 33,974,052 | 0.1078 | 1.75x10^-5^ | 0.0006 | 0.0002 |
| BOVINEHD0600009854 | 6 | 33,991,326 | 0.1080 | 1.89x10^-5^ | 0.0006 | 0.0002 |
| BOVINEHD0600009855 | 6 | 33,991,862 | 0.1080 | 1.95x10^-5^ | 0.0006 | 0.0002 |
| BOVINEHD0600009858 | 6 | 34,006,110 | 0.1079 | 1.85x10^-5^ | 0.0006 | 0.0002 |
| BOVINEHD0600009859 | 6 | 34,007,025 | 0.1079 | 1.92x10^-5^ | 0.0006 | 0.0002 |
| BOVINEHD0600009860 | 6 | 34,007,974 | 0.8921 | 1.92x10^-5^ | 0.0006 | -0.0002 |
| BOVINEHD0600009861 | 6 | 34,008,851 | 0.8921 | 1.92x10^-5^ | 0.0006 | -0.0002 |
| BOVINEHD0600009862 | 6 | 34,011,629 | 0.8921 | 1.92x10^-5^ | 0.0006 | -0.0002 |
| BOVINEHD0600009863 | 6 | 34,012,470 | 0.1079 | 1.95x10^-5^ | 0.0006 | 0.0002 |
| BOVINEHD0600009864 | 6 | 34,015,505 | 0.1079 | 1.95x10^-5^ | 0.0006 | 0.0002 |
| BOVINEHD0600009865 | 6 | 34,016,254 | 0.8921 | 1.92x10^-5^ | 0.0006 | -0.0002 |
| HAPMAP27887_BTA_156628 | 6 | 34,018,647 | 0.8921 | 1.92x10^-5^ | 0.0006 | -0.0002 |
| BOVINEHD0600009866 | 6 | 34,020,118 | 0.1080 | 1.95x10^-5^ | 0.0006 | 0.0002 |
| BOVINEHD0600009867 | 6 | 34,021,322 | 0.8921 | 1.89x10^-5^ | 0.0006 | -0.0002 |
| BOVINEHD0600009868 | 6 | 34,021,807 | 0.1080 | 1.92x10^-5^ | 0.0006 | 0.0002 |
| BOVINEHD0600009870 | 6 | 34,023,559 | 0.8921 | 1.86x10^-5^ | 0.0006 | -0.0002 |
| BOVINEHD0600009871 | 6 | 34,024,620 | 0.8920 | 1.92x10^-5^ | 0.0006 | -0.0002 |
| BOVINEHD0600009872 | 6 | 34,025,427 | 0.8920 | 1.92x10^-5^ | 0.0006 | -0.0002 |
| BOVINEHD0600009873 | 6 | 34,027,236 | 0.8920 | 1.92x10^-5^ | 0.0006 | -0.0002 |
| BOVINEHD0600009874 | 6 | 34,030,066 | 0.1080 | 1.95x10^-5^ | 0.0006 | 0.0002 |
| BOVINEHD0600009876 | 6 | 34,036,502 | 0.8920 | 1.49x10^-5^ | 0.0006 | -0.0002 |
| BOVINEHD0600009878 | 6 | 34,038,779 | 0.1080 | 1.49x10^-5^ | 0.0006 | 0.0002 |
| BOVINEHD0600009886 | 6 | 34,058,212 | 0.8916 | 7.01x10^-6^ | 0.0006 | -0.0002 |
| BOVINEHD0600009905 | 6 | 34,094,048 | 0.8916 | 6.87x10^-6^ | 0.0006 | -0.0002 |
| BOVINEHD0600009907 | 6 | 34,099,075 | 0.1084 | 7.01x10^-6^ | 0.0006 | 0.0002 |
| BOVINEHD0600009908 | 6 | 34,102,026 | 0.8916 | 6.91x10^-6^ | 0.0006 | -0.0002 |
| BOVINEHD0600009909 | 6 | 34,103,103 | 0.1084 | 7.01x10^-6^ | 0.0006 | 0.0002 |
| BOVINEHD0600009910 | 6 | 34,106,261 | 0.8914 | 6.84x10^-6^ | 0.0006 | -0.0002 |
| BOVINEHD0600009911 | 6 | 34,110,281 | 0.8916 | 7.01x10^-6^ | 0.0006 | -0.0002 |
| BOVINEHD0600009913 | 6 | 34,116,385 | 0.1084 | 6.91x10^-6^ | 0.0006 | 0.0002 |
| BOVINEHD0600009914 | 6 | 34,125,483 | 0.8916 | 6.87x10^-6^ | 0.0006 | -0.0002 |
| HAPMAP50194_BTA_105136 | 6 | 34,139,287 | 0.8917 | 6.87x10^-6^ | 0.0006 | -0.0002 |
| BOVINEHD4100004430 | 6 | 34,142,575 | 0.1084 | 6.91x10^-6^ | 0.0006 | 0.0002 |
| BOVINEHD0600009918 | 6 | 34,145,380 | 0.1084 | 7.01x10^-6^ | 0.0006 | 0.0002 |
| BOVINEHD0600009919 | 6 | 34,147,707 | 0.8916 | 6.91x10^-6^ | 0.0006 | -0.0002 |
| BOVINEHD0600009921 | 6 | 34,153,252 | 0.8916 | 6.91x10^-6^ | 0.0006 | -0.0002 |
| BOVINEHD0600009923 | 6 | 34,162,802 | 0.8916 | 6.91x10^-6^ | 0.0006 | -0.0002 |
| BOVINEHD0600009924 | 6 | 34,167,727 | 0.1084 | 7.01x10^-6^ | 0.0006 | 0.0002 |
| BOVINEHD0600009931 | 6 | 34,200,926 | 0.1083 | 8.96x10^-6^ | 0.0006 | 0.0002 |
| BOVINEHD0600009955 | 6 | 34,312,505 | 0.1086 | 9.54x10^-6^ | 0.0006 | 0.0002 |
| BOVINEHD0600009958 | 6 | 34,317,610 | 0.1086 | 9.54x10^-6^ | 0.0006 | 0.0002 |
| BOVINEHD0600009962 | 6 | 34,327,017 | 0.8915 | 9.41x10^-6^ | 0.0006 | -0.0002 |
| BOVINEHD0600009985 | 6 | 34,421,298 | 0.8914 | 4.49x10^-6^ | 0.0007 | -0.0002 |
| BOVINEHD0600009994 | 6 | 34,461,838 | 0.8672 | 1.32x10^-5^ | 0.0011 | -0.0002 |
| BOVINEHD0600010002 | 6 | 34,489,711 | 0.8914 | 5.64x10^-6^ | 0.0006 | -0.0002 |
| BOVINEHD0600010004 | 6 | 34,494,702 | 0.8914 | 5.53x10^-6^ | 0.0007 | -0.0002 |
| BOVINEHD4100004432 | 6 | 34,504,122 | 0.1086 | 4.17x10^-6^ | 0.0007 | 0.0002 |
| BOVINEHD0600010008 | 6 | 34,519,212 | 0.8913 | 9.54x10^-6^ | 0.0006 | -0.0002 |
| BOVINEHD0600010009 | 6 | 34,523,354 | 0.8914 | 9.54x10^-6^ | 0.0006 | -0.0002 |
| BOVINEHD0600010025 | 6 | 34,574,321 | 0.8914 | 1.07x10^-5^ | 0.0006 | -0.0002 |
| BOVINEHD0600010032 | 6 | 34,612,574 | 0.8916 | 6.98x10^-6^ | 0.0006 | -0.0002 |
| BOVINEHD0600010407 | 6 | 36,072,095 | 0.8623 | 1.92x10^-5^ | 0.0013 | -0.0002 |
| HAPMAP26263_BTC_036885 | 6 | 36,074,911 | 0.1204 | 3.12x10^-6^ | 0.0012 | 0.0002 |
| BOVINEHD0600010408 | 6 | 36,076,799 | 0.8795 | 2.37x10^-6^ | 0.0012 | -0.0002 |
| BOVINEHD0600010409 | 6 | 36,086,492 | 0.1377 | 1.92x10^-5^ | 0.0013 | 0.0002 |
| BOVINEHD4100004494 | 6 | 36,108,228 | 0.8848 | 3.27x10^-8^ | 0.0013 | -0.0003 |
| BOVINEHD0600010412 | 6 | 36,112,726 | 0.8809 | 4.43x10^-8^ | 0.0014 | -0.0003 |
| BOVINEHD0600010726 | 6 | 37,301,959 | 0.8425 | 3.08x10^-7^ | 0.0025 | -0.0003 |
| BOVINEHD0600010737 | 6 | 37,331,712 | 0.1570 | 1.97x10^-7^ | 0.0026 | 0.0003 |
| BOVINEHD0600010739 | 6 | 37,340,937 | 0.1570 | 1.97x10^-7^ | 0.0026 | 0.0003 |
| BOVINEHD0600010740 | 6 | 37,342,568 | 0.8431 | 2.05x10^-7^ | 0.0026 | -0.0003 |
| BOVINEHD0600010741 | 6 | 37,345,508 | 0.1570 | 1.99x10^-7^ | 0.0026 | 0.0003 |
| BOVINEHD0600010743 | 6 | 37,349,144 | 0.8431 | 2.05x10^-7^ | 0.0026 | -0.0003 |
| BOVINEHD0600010746 | 6 | 37,369,380 | 0.8431 | 2.05x10^-7^ | 0.0026 | -0.0003 |
| BOVINEHD4100004579 | 6 | 37,407,653 | 0.8428 | 3.63x10^-7^ | 0.0025 | -0.0003 |
| HAPMAP23507_BTC_041133 | 6 | 37,412,062 | 0.8428 | 3.59x10^-7^ | 0.0025 | -0.0003 |
| BOVINEHD0600010771 | 6 | 37,550,562 | 0.1578 | 1.45x10^-7^ | 0.0026 | 0.0003 |
| BOVINEHD4100004597 | 6 | 37,782,521 | 0.7911 | 1.23x10^-5^ | 0.0030 | -0.0003 |
| BOVINEHD0600010800 | 6 | 37,786,966 | 0.2082 | 9.86x10^-6^ | 0.0031 | 0.0003 |
| BOVINEHD0600010801 | 6 | 37,787,672 | 0.7913 | 1.31x10^-5^ | 0.0030 | -0.0003 |
| BOVINEHD4100004601 | 6 | 37,802,917 | 0.7913 | 1.31x10^-5^ | 0.0030 | -0.0003 |
| BOVINEHD4100004620 | 6 | 37,929,882 | 0.7312 | 1.85x10^-5^ | 0.0044 | -0.0003 |
| BOVINEHD0600010840 | 6 | 37,991,751 | 0.8644 | 3.42x10^-8^ | 0.0021 | -0.0003 |
| BOVINEHD0600011120 | 6 | 39,414,796 | 0.8952 | 1.98x10^-7^ | 0.0011 | -0.0002 |
| BOVINEHD0600012569 | 6 | 44,629,924 | 0.2991 | 6.13x10^-6^ | 0.0054 | 0.0004 |
| BOVINEHD0600012810 | 6 | 45,341,908 | 0.4757 | 1.99x10^-5^ | 0.0070 | -0.0004 |
| BOVINEHD0600013007 | 6 | 46,011,640 | 0.9126 | 8.41x10^-6^ | 0.0005 | -0.0002 |
| BOVINEHD0600013008 | 6 | 46,016,114 | 0.0874 | 8.48x10^-6^ | 0.0005 | 0.0002 |
| BOVINEHD0600013011 | 6 | 46,029,330 | 0.9126 | 8.48x10^-6^ | 0.0005 | -0.0002 |
| BOVINEHD0600013013 | 6 | 46,035,432 | 0.0874 | 8.41x10^-6^ | 0.0005 | 0.0002 |
| BTA_22339_NO_RS | 6 | 46,114,033 | 0.9127 | 4.09x10^-6^ | 0.0005 | -0.0002 |
| BOVINEHD0600013047 | 6 | 46,126,112 | 0.9108 | 1.63x10^-5^ | 0.0005 | -0.0002 |
| BOVINEHD0600013048 | 6 | 46,126,660 | 0.0892 | 1.66x10^-5^ | 0.0005 | 0.0002 |
| BOVINEHD0600013049 | 6 | 46,129,399 | 0.9108 | 1.65x10^-5^ | 0.0005 | -0.0002 |
| BOVINEHD0600013053 | 6 | 46,148,590 | 0.9108 | 6.53x10^-6^ | 0.0005 | -0.0002 |
| BTA_76122_NO_RS | 6 | 46,167,897 | 0.9110 | 5.43x10^-6^ | 0.0005 | -0.0002 |
| BOVINEHD0600013061 | 6 | 46,181,398 | 0.9110 | 5.48x10^-6^ | 0.0005 | -0.0002 |
| BOVINEHD0600013081 | 6 | 46,243,650 | 0.0874 | 3.90x10^-6^ | 0.0005 | 0.0002 |
| BOVINEHD1000030754 | 10 | 72,865,996 | 0.8914 | 1.11x10^-5^ | 0.0014 | -0.0003 |
| BOVINEHD1100019768 | 11 | 69,568,275 | 0.1165 | 8.30x10^-6^ | 0.0015 | 0.0003 |
| BOVINEHD1400001642 | 14 | 5,137,561 | 0.7288 | 2.23x10^-6^ | 0.0067 | 0.0004 |
| BOVINEHD1400007161 | 14 | 22,999,212 | 0.7006 | 2.20x10^-5^ | 0.0016 | -0.0002 |
| BOVINEHD1900015419 | 19 | 54,287,373 | 0.8741 | 4.57x10^-6^ | 0.0020 | 0.0003 |
| BOVINEHD2000006349 | 20 | 21,223,790 | 0.5766 | 6.56x10^-6^ | 0.0085 | -0.0004 |
| BOVINEHD2200005412 | 22 | 18,710,302 | 0.4694 | 8.41x10^-6^ | 0.0083 | 0.0004 |
| BOVINEHD2200013355 | 22 | 45,863,244 | 0.1750 | 2.66x10^-6^ | 0.0034 | 0.0003 |
| BOVINEHD2200013649 | 22 | 46,897,299 | 0.4994 | 8.07x10^-6^ | 0.0090 | -0.0004 |
| ARS_BFGL_NGS_14050 | 29 | 46,844,718 | 0.8202 | 1.91x10^-5^ | 0.0031 | -0.0003 |
| BOVINEHD2900014052 | 29 | 46,847,971 | 0.2248 | 2.83x10^-5^ | 0.0045 | 0.0004 |
| BOVINEHD2900015606 | 29 | 46,852,455 | 0.1895 | 3.54x10^-5^ | 0.0032 | 0.0003 |
| BOVINEHD2900014059 | 29 | 46,865,487 | 0.8154 | 2.98x10^-5^ | 0.0031 | -0.0003 |

Medium (THI 74).

**Table S10.** Significant SNPs in the high environmental gradient (EG) associated with dry matter intake (DMI) in Nellore cattle: chromosome (BTA), position (bp), allele frequency (F), proportion of the total additive genetic variance explained (var %), and effect.

| SNP | BTA | Position (bp) | F | p-value | var (%) | effect |
| --- | --- | --- | --- | --- | --- | --- |
| BOVINEHD0400014486 | 4 | 52,213,076 | 0.8314 | 2.86x10^-06^ | 0.0025 | -0.0003 |
| BOVINEHD0600008722 | 6 | 29,684,500 | 0.9111 | 5.59x10^-06^ | 0.0005 | -0.0002 |
| HAPMAP54442_RS29025673 | 6 | 29,700,842 | 0.0892 | 2.21x10^-06^ | 0.0005 | 0.0002 |
| HAPMAP32447_BTC_033214 | 6 | 32,254,947 | 0.9019 | 1.05x10^-05^ | 0.0005 | -0.0002 |
| BOVINEHD0600009439 | 6 | 32,339,668 | 0.0989 | 1.88x10^-05^ | 0.0005 | 0.0002 |
| BOVINEHD0600009468 | 6 | 32,453,198 | 0.0980 | 5.92x10^-06^ | 0.0005 | 0.0002 |
| BOVINEHD0600009469 | 6 | 32,456,964 | 0.0974 | 3.57x10^-06^ | 0.0006 | 0.0002 |
| BOVINEHD0600009470 | 6 | 32,461,586 | 0.9027 | 2.96x10^-06^ | 0.0006 | -0.0002 |
| BOVINEHD0600009471 | 6 | 32,462,575 | 0.0974 | 3.57x10^-06^ | 0.0006 | 0.0002 |
| BOVINEHD0600009483 | 6 | 32,499,982 | 0.0974 | 2.99x10^-06^ | 0.0006 | 0.0002 |
| BOVINEHD0600009555 | 6 | 32,763,336 | 0.9032 | 1.14x10^-05^ | 0.0005 | -0.0002 |
| BOVINEHD0600009802 | 6 | 33,747,292 | 0.8678 | 9.91x10^-06^ | 0.0012 | -0.0002 |
| BOVINEHD0600009803 | 6 | 33,747,860 | 0.1322 | 9.92x10^-06^ | 0.0012 | 0.0002 |
| BOVINEHD0600009804 | 6 | 33,752,201 | 0.8675 | 5.82x10^-06^ | 0.0012 | -0.0002 |
| BOVINEHD0600034663 | 6 | 33,788,813 | 0.8483 | 4.51x10^-06^ | 0.0017 | -0.0003 |
| BOVINEHD0600009886 | 6 | 34,058,212 | 0.8916 | 1.42x10^-05^ | 0.0006 | -0.0002 |
| BOVINEHD0600009905 | 6 | 34,094,048 | 0.8916 | 1.39x10^-05^ | 0.0006 | -0.0002 |
| BOVINEHD0600009907 | 6 | 34,099,075 | 0.1084 | 1.42x10^-05^ | 0.0006 | 0.0002 |
| BOVINEHD0600009908 | 6 | 34,102,026 | 0.8916 | 1.40x10^-05^ | 0.0006 | -0.0002 |
| BOVINEHD0600009909 | 6 | 34,103,103 | 0.1084 | 1.42x10^-05^ | 0.0006 | 0.0002 |
| BOVINEHD0600009910 | 6 | 34,106,261 | 0.8914 | 1.39x10^-05^ | 0.0006 | -0.0002 |
| BOVINEHD0600009911 | 6 | 34,110,281 | 0.8916 | 1.42x10^-05^ | 0.0006 | -0.0002 |
| BOVINEHD0600009913 | 6 | 34,116,385 | 0.1084 | 1.40x10^-05^ | 0.0006 | 0.0002 |
| BOVINEHD0600009914 | 6 | 34,125,483 | 0.8916 | 1.39x10^-05^ | 0.0006 | -0.0002 |
| HAPMAP50194_BTA_105136 | 6 | 34,139,287 | 0.8917 | 1.39x10^-05^ | 0.0006 | -0.0002 |
| BOVINEHD4100004430 | 6 | 34,142,575 | 0.1084 | 1.40x10^-05^ | 0.0006 | 0.0002 |
| BOVINEHD0600009918 | 6 | 34,145,380 | 0.1084 | 1.42x10^-05^ | 0.0006 | 0.0002 |
| BOVINEHD0600009919 | 6 | 34,147,707 | 0.8916 | 1.40x10^-05^ | 0.0006 | -0.0002 |
| BOVINEHD0600009921 | 6 | 34,153,252 | 0.8916 | 1.40x10^-05^ | 0.0006 | -0.0002 |
| BOVINEHD0600009923 | 6 | 34,162,802 | 0.8916 | 1.40x10^-05^ | 0.0006 | -0.0002 |
| BOVINEHD0600009924 | 6 | 34,167,727 | 0.1084 | 1.42x10^-05^ | 0.0006 | 0.0002 |
| BOVINEHD0600009931 | 6 | 34,200,926 | 0.1083 | 1.83x10^-05^ | 0.0006 | 0.0002 |
| BOVINEHD0600009955 | 6 | 34,312,505 | 0.1086 | 1.94x10^-05^ | 0.0006 | 0.0002 |
| BOVINEHD0600009958 | 6 | 34,317,610 | 0.1086 | 1.94x10^-05^ | 0.0006 | 0.0002 |
| BOVINEHD0600009962 | 6 | 34,327,017 | 0.8915 | 1.91x10^-05^ | 0.0006 | -0.0002 |
| BOVINEHD0600009985 | 6 | 34,421,298 | 0.8914 | 9.09x10^-06^ | 0.0006 | -0.0002 |
| BOVINEHD0600010002 | 6 | 34,489,711 | 0.8914 | 1.14x10^-05^ | 0.0006 | -0.0002 |
| BOVINEHD0600010004 | 6 | 34,494,702 | 0.8914 | 1.12x10^-05^ | 0.0006 | -0.0002 |
| BOVINEHD4100004432 | 6 | 34,504,122 | 0.1086 | 8.35x10^-06^ | 0.0006 | 0.0002 |
| BOVINEHD0600010008 | 6 | 34,519,212 | 0.8913 | 1.96x10^-05^ | 0.0006 | -0.0002 |
| BOVINEHD0600010009 | 6 | 34,523,354 | 0.8914 | 1.96x10^-05^ | 0.0006 | -0.0002 |
| BOVINEHD0600010032 | 6 | 34,612,574 | 0.8916 | 1.42x10^-05^ | 0.0006 | -0.0002 |
| HAPMAP26263_BTC_036885 | 6 | 36,074,911 | 0.1204 | 4.53x10^-06^ | 0.0011 | 0.0003 |
| BOVINEHD0600010408 | 6 | 36,076,799 | 0.8795 | 3.42x10^-06^ | 0.0012 | -0.0003 |
| BOVINEHD4100004494 | 6 | 36,108,228 | 0.8848 | 5.56x10^-08^ | 0.0013 | -0.0003 |
| BOVINEHD0600010412 | 6 | 36,112,726 | 0.8809 | 6.90x10^-08^ | 0.0014 | -0.0003 |
| BOVINEHD0600010726 | 6 | 37,301,959 | 0.8425 | 4.46x10^-07^ | 0.0024 | -0.0003 |
| BOVINEHD0600010737 | 6 | 37,331,712 | 0.1570 | 2.87x10^-07^ | 0.0025 | 0.0003 |
| BOVINEHD0600010739 | 6 | 37,340,937 | 0.1570 | 2.87x10^-07^ | 0.0025 | 0.0003 |
| BOVINEHD0600010740 | 6 | 37,342,568 | 0.8431 | 2.98x10^-07^ | 0.0025 | -0.0003 |
| BOVINEHD0600010741 | 6 | 37,345,508 | 0.1570 | 2.90x10^-07^ | 0.0025 | 0.0003 |
| BOVINEHD0600010743 | 6 | 37,349,144 | 0.8431 | 2.98x10^-07^ | 0.0025 | -0.0003 |
| BOVINEHD0600010746 | 6 | 37,369,380 | 0.8431 | 2.98x10^-07^ | 0.0025 | -0.0003 |
| BOVINEHD4100004579 | 6 | 37,407,653 | 0.8428 | 5.35x10^-07^ | 0.0024 | -0.0003 |
| HAPMAP23507_BTC_041133 | 6 | 37,412,062 | 0.8428 | 5.28x10^-07^ | 0.0024 | -0.0003 |
| BOVINEHD0600010771 | 6 | 37,550,562 | 0.1578 | 2.04x10^-07^ | 0.0026 | 0.0003 |
| BOVINEHD4100004597 | 6 | 37,782,521 | 0.7911 | 1.60x10^-05^ | 0.0030 | -0.0003 |
| BOVINEHD0600010800 | 6 | 37,786,966 | 0.2082 | 1.25x10^-05^ | 0.0030 | 0.0003 |
| BOVINEHD0600010801 | 6 | 37,787,672 | 0.7913 | 1.69x10^-05^ | 0.0029 | -0.0003 |
| BOVINEHD4100004601 | 6 | 37,802,917 | 0.7913 | 1.69x10^-05^ | 0.0029 | -0.0003 |
| BOVINEHD0600010840 | 6 | 37,991,751 | 0.8644 | 4.86x10^-08^ | 0.0021 | -0.0003 |
| BOVINEHD0600011120 | 6 | 39,414,796 | 0.8952 | 3.53x10^-07^ | 0.0010 | -0.0003 |
| BOVINEHD0600012569 | 6 | 44,629,924 | 0.2991 | 7.79x10^-06^ | 0.0053 | 0.0004 |
| BOVINEHD0600013007 | 6 | 46,011,640 | 0.9126 | 1.93x10^-05^ | 0.0004 | -0.0002 |
| BOVINEHD0600013008 | 6 | 46,016,114 | 0.0874 | 1.94x10^-05^ | 0.0004 | 0.0002 |
| BOVINEHD0600013011 | 6 | 46,029,330 | 0.9126 | 1.94x10^-05^ | 0.0004 | -0.0002 |
| BOVINEHD0600013013 | 6 | 46,035,432 | 0.0874 | 1.93x10^-05^ | 0.0004 | 0.0002 |
| BTA_22339_NO_RS | 6 | 46,114,033 | 0.9127 | 9.28x10^-06^ | 0.0005 | -0.0002 |
| BOVINEHD0600013053 | 6 | 46,148,590 | 0.9108 | 1.47x10^-05^ | 0.0005 | -0.0002 |
| BTA_76122_NO_RS | 6 | 46,167,897 | 0.9110 | 1.24x10^-05^ | 0.0005 | -0.0002 |
| BOVINEHD0600013061 | 6 | 46,181,398 | 0.9110 | 1.25x10^-05^ | 0.0005 | -0.0002 |
| BOVINEHD0600013081 | 6 | 46,243,650 | 0.0874 | 8.88x10^-06^ | 0.0005 | 0.0002 |
| BOVINEHD1000030754 | 10 | 72,865,996 | 0.8914 | 1.07x10^-05^ | 0.0014 | -0.0003 |
| BOVINEHD1100019768 | 11 | 69,568,275 | 0.1165 | 7.78x10^-06^ | 0.0016 | 0.0003 |
| BOVINEHD1400001642 | 14 | 5,137,561 | 0.7288 | 2.32x10^-06^ | 0.0067 | 0.0004 |
| BOVINEHD1400007161 | 14 | 22,999,212 | 0.7006 | 2.61x10^-05^ | 0.0016 | -0.0002 |
| BOVINEHD1900015419 | 19 | 54,287,373 | 0.8741 | 3.79x10^-06^ | 0.0021 | 0.0003 |
| BOVINEHD2000006349 | 20 | 21,223,790 | 0.5766 | 6.88x10^-06^ | 0.0085 | -0.0005 |
| BOVINEHD2200005412 | 22 | 18,710,302 | 0.4694 | 9.42x10^-06^ | 0.0082 | 0.0004 |
| BOVINEHD2200013355 | 22 | 45,863,244 | 0.1750 | 2.41x10^-06^ | 0.0035 | 0.0004 |
| BOVINEHD2200013649 | 22 | 46,897,299 | 0.4994 | 9.18x10^-06^ | 0.0089 | -0.0005 |
| ARS_BFGL_NGS_14050 | 29 | 46,844,718 | 0.8202 | 1.82x10^-05^ | 0.0032 | -0.0004 |
| BOVINEHD2900014052 | 29 | 46,847,971 | 0.2248 | 2.67x10^-05^ | 0.0045 | 0.0004 |
| BOVINEHD2900015606 | 29 | 46,852,455 | 0.1895 | 3.40x10^-05^ | 0.0033 | 0.0004 |
| BOVINEHD2900014059 | 29 | 46,865,487 | 0.8154 | 2.91x10^-05^ | 0.0032 | -0.0004 |

High (THI 81).

**Table S11.** Candidate genes associated with significant SNPs for residual feed intake (RFI) in Nellore cattle at the low environmental gradient (THI 66).

| BTA | SNP | Gene | Start gene position (bp) | End gene position (bp) | Gene biotype |
| --- | --- | --- | --- | --- | --- |
| 3 | BOVINEHD0300035625 | *NFIA* | 84,203,465 | 84,816,448 | protein_coding |
| 9 | BOVINEHD0900024567 | *UST* | 86,010,521 | 86,326,703 | protein_coding |
| 9 | BOVINEHD0900005087 | *PHIP* | 18,522,256 | 18,646,824 | protein_coding |
| 9 | BOVINEHD0900005087 | *IRAK1BP1* | 18,468,160 | 18,481,224 | protein_coding |
| 11 | BOVINEHD1100006650 | *TMEM178A* | 21,962,641 | 22,020,613 | protein_coding |
| 11 | BOVINEHD1100006650 | *THUMPD2* | 22,040,726 | 22,080,776 | protein_coding |
| 12 | BOVINEHD1200004514 | *NUFIP1* | 15,118,007 | 15,157,140 | protein_coding |
| 12 | BOVINEHD1200004514 | *GPALPP1* | 15,157,012 | 15,191,157 | protein_coding |
| 12 | BOVINEHD1200004607 | *SLC25A30* | 15,489,400 | 15,573,616 | protein_coding |
| 12 | BOVINEHD1200004543 | *GTF2F2* | 15,282,784 | 15,418,923 | protein_coding |
| 12 | BOVINEHD1200004564 | *KCTD4* | 15,339,146 | 15,350,049 | protein_coding |
| 12 | BOVINEHD1200004601 | *SNORA31* | 15,472,536 | 15,472,667 | snoRNA |
| 13 | ARS_BFGL_NGS_109467 | *PHYH* | 27,975,608 | 27,996,366 | protein_coding |
| 13 | ARS_BFGL_NGS_109467 | *SEPHS1* | 28,008,899 | 28,031,413 | protein_coding |
| 13 | ARS_BFGL_NGS_109467 | *BEND7* | 28,141,625 | 28,228,506 | protein_coding |
| 19 | BOVINEHD1900005359 | *RAB11FIP4* | 18,500,131 | 18,602,788 | protein_coding |
| 19 | BOVINEHD1900005359 | *SNORA70* | 18,413,980 | 18,414,114 | snoRNA |
| 19 | BOVINEHD1900005359 | *U6* | 18,359,654 | 18,359,760 | snRNA |
| 19 | BOVINEHD1900005359 | *U7* | 18,339,606 | 18,339,667 | snRNA |
| 19 | BOVINEHD1900005359 | *U2* | 18,357,916 | 18,358,065 | snRNA |
| 19 | BOVINEHD1900005359 | *bta-mir-2333* | 18,463,796 | 18,463,858 | miRNA |
| 19 | BOVINEHD1900005359 | *bta-mir-365-2* | 18,461,502 | 18,461,612 | miRNA |
| 19 | BOVINEHD1900005359 | *bta-mir-193a* | 18,474,461 | 18,474,541 | miRNA |
| 19 | BOVINEHD4100014031 | *PIPOX* | 20,406,351 | 20,419,985 | protein_coding |
| 19 | BOVINEHD4100014031 | *MYO18A* | 20,435,389 | 20,536,478 | protein_coding |
| 19 | BOVINEHD4100014031 | *SEZ6* | 20,320,602 | 20,368,753 | protein_coding |
| 19 | BOVINEHD1900006041 | *SNORA72* | 20,543,611 | 20,543,742 | snoRNA |
| 19 | BOVINEHD1900012010 | *KRT31* | 41,615,468 | 41,619,464 | protein_coding |
| 19 | BOVINEHD1900012010 | *KRT37* | 41,627,472 | 41,634,832 | protein_coding |
| 19 | BOVINEHD1900012010 | *KRT36* | 41,678,479 | 41,695,018 | protein_coding |
| 19 | BOVINEHD1900012010 | *KRTAP17-1* | 41,527,379 | 41,527,714 | protein_coding |
| 19 | BOVINEHD1900012010 | *KRT33A* | 41,552,389 | 41,597,743 | protein_coding |
| 19 | BOVINEHD1900012010 | *KRT32* | 41,666,475 | 41,674,684 | protein_coding |
| 28 | BOVINEHD2800001982 | *KCNK1* | 06,451,567 | 06,519,065 | protein_coding |
| 28 | BOVINEHD2800002037 | *SLC35F3* | 06,720,641 | 07,155,373 | protein_coding |

**Table S12.** Candidate genes associated with significant SNPs for residual feed intake (RFI) in Nellore cattle at the medium environmental gradient (THI 74).

| BTA | SNP | Gene | Start gene position (bp) | End gene position (bp) | Gene biotype |
| --- | --- | --- | --- | --- | --- |
| 3 | BOVINEHD0300035625 | *NFIA* | 84,203,465 | 84,816,448 | protein_coding |
| 12 | BOVINEHD1200004514 | *NUFIP1* | 15,118,007 | 15,157,140 | protein_coding |
| 12 | BOVINEHD1200004514 | *GPALPP1* | 15,157,012 | 15,191,157 | protein_coding |
| 12 | BOVINEHD1200004607 | *SLC25A30* | 15,489,400 | 15,573,616 | protein_coding |
| 12 | BOVINEHD1200004538 | *GTF2F2* | 15,282,784 | 15,418,923 | protein_coding |
| 12 | BOVINEHD1200004573 | *KCTD4* | 15,339,146 | 15,350,049 | protein_coding |
| 12 | BOVINEHD1200004601 | *SNORA31* | 15,472,536 | 15,472,667 | snoRNA |
| 19 | BOVINEHD1900005359 | *RAB11FIP4* | 18,500,131 | 18,602,788 | protein_coding |
| 19 | BOVINEHD1900004927 | *ASIC2* | 16,932,407 | 17,228,957 | protein_coding |
| 19 | BOVINEHD1900004927 | *U6* | 17,158,687 | 17,158,793 | snRNA |
| 19 | BOVINEHD1900005359 | *SNORA70* | 18,413,980 | 18,414,114 | snoRNA |
| 19 | BOVINEHD1900005359 | *U7* | 18,339,606 | 18,339,667 | snRNA |
| 19 | BOVINEHD1900005359 | *U2* | 18,357,916 | 18,358,065 | snRNA |
| 19 | BOVINEHD1900005359 | *bta-mir-2333* | 18,463,796 | 18,463,858 | miRNA |
| 19 | BOVINEHD1900005359 | *bta-mir-365-2* | 18,461,502 | 18,461,612 | miRNA |
| 19 | BOVINEHD1900005359 | *bta-mir-193a* | 18,474,461 | 18,474,541 | miRNA |
| 19 | BOVINEHD4100014031 | *PIPOX* | 20,406,351 | 20,419,985 | protein_coding |
| 19 | BOVINEHD4100014031 | *MYO18A* | 20,435,389 | 20,536,478 | protein_coding |
| 19 | BOVINEHD4100014031 | *SEZ6* | 20,320,602 | 20,368,753 | protein_coding |
| 19 | BOVINEHD1900006041 | *SNORA72* | 20,543,611 | 20,543,742 | snoRNA |
| 19 | BOVINEHD1900011755 | *TOP2A* | 40,626,894 | 40,653,419 | protein_coding |
| 19 | BOVINEHD1900011755 | *IGFBP4* | 40,672,669 | 40,684,245 | protein_coding |
| 19 | BOVINEHD1900011755 | *TNS4* | 40,696,965 | 40,720,512 | protein_coding |
| 19 | BOVINEHD1900011755 | *CCR7* | 40,775,267 | 40,786,420 | protein_coding |
| 19 | BOVINEHD1900011755 | *RARA* | 40,560,016 | 40,595,283 | protein_coding |
| 19 | BOVINEHD1900011755 | *GJD3* | 40,605,340 | 40,606,828 | protein_coding |
| 19 | BOVINEHD1900011755 | *bta-mir-2285cb* | 40,752,649 | 40,752,706 | miRNA |
| 24 | BOVINEHD2400006188 | *DTNA* | 22,130,555 | 22,449,866 | protein_coding |

**Table S13.** Candidate genes associated with significant SNPs for residual feed intake (RFI) in Nellore cattle at the high environmental gradient (THI 81).

| BTA | SNP | Gene | Start gene position (bp) | End gene position (bp) | Gene biotype |
| --- | --- | --- | --- | --- | --- |
| 3 | BOVINEHD0300035625 | *NFIA* | 84,203,465 | 84,816,448 | protein_coding |
| 12 | BOVINEHD1200004514 | *NUFIP1* | 15,118,007 | 15,157,140 | protein_coding |
| 12 | BOVINEHD1200004514 | *GPALPP1* | 15,157,012 | 15,191,157 | protein_coding |
| 12 | BOVINEHD1200004607 | *SLC25A30* | 15,489,400 | 15,573,616 | protein_coding |
| 12 | BOVINEHD1200004538 | *GTF2F2* | 15,282,784 | 15,418,923 | protein_coding |
| 12 | BOVINEHD1200004573 | *KCTD4* | 15,339,146 | 15,350,049 | protein_coding |
| 12 | BOVINEHD1200004601 | *SNORA31* | 15,472,536 | 15,472,667 | snoRNA |
| 19 | BOVINEHD1900005359 | *RAB11FIP4* | 18,500,131 | 18,602,788 | protein_coding |
| 19 | BOVINEHD1900004927 | *ASIC2* | 16,932,407 | 17,228,957 | protein_coding |
| 19 | BOVINEHD1900004927 | *U6* | 17,158,687 | 17,158,793 | snRNA |
| 19 | BOVINEHD1900005359 | *SNORA70* | 18,413,980 | 18,414,114 | snoRNA |
| 19 | BOVINEHD1900005359 | *U7* | 18,339,606 | 18,339,667 | snRNA |
| 19 | BOVINEHD1900005359 | *U2* | 18,357,916 | 18,358,065 | snRNA |
| 19 | BOVINEHD1900005359 | *bta-mir-2333* | 18,463,796 | 18,463,858 | miRNA |
| 19 | BOVINEHD1900005359 | *bta-mir-365-2* | 18,461,502 | 18,461,612 | miRNA |
| 19 | BOVINEHD1900005359 | *bta-mir-193a* | 18,474,461 | 18,474,541 | miRNA |
| 19 | BOVINEHD4100014031 | *PIPOX* | 20,406,351 | 20,419,985 | protein_coding |
| 19 | BOVINEHD4100014031 | *MYO18A* | 20,435,389 | 20,536,478 | protein_coding |
| 19 | BOVINEHD4100014031 | *SEZ6* | 20,320,602 | 20,368,753 | protein_coding |
| 19 | BOVINEHD1900006041 | *SNORA72* | 20,543,611 | 20,543,742 | snoRNA |
| 19 | BOVINEHD1900011747 | *TOP2A* | 40,626,894 | 40,653,419 | protein_coding |
| 19 | BOVINEHD1900011747 | *IGFBP4* | 40,672,669 | 40,684,245 | protein_coding |
| 19 | BOVINEHD1900011747 | *TNS4* | 40,696,965 | 40,720,512 | protein_coding |
| 19 | BOVINEHD1900011755 | *CCR7* | 40,775,267 | 40,786,420 | protein_coding |
| 19 | BOVINEHD1900011747 | *RARA* | 40,560,016 | 40,595,283 | protein_coding |
| 19 | BOVINEHD1900011747 | *GJD3* | 40,605,340 | 40,606,828 | protein_coding |
| 19 | BOVINEHD1900011755 | *bta-mir-2285cb* | 40,752,649 | 40,752,706 | miRNA |
| 24 | BOVINEHD2400006188 | *DTNA* | 22,130,555 | 22,449,866 | protein_coding |

**Table S14.** Candidate genes associated with significant SNPs for dry matter intake (DMI) in Nellore cattle at the low environmental gradient (THI 66).

| BTA | SNP | Gene | Start gene position (bp) | End gene position (bp) | Gene biotype |
| --- | --- | --- | --- | --- | --- |
| 2 | BOVINEHD0200007602 | *MYO3B* | 25,780,219 | 26,299,702 | protein_coding |
| 2 | BOVINEHD0200007602 | *UBR3* | 26,313,298 | 26,517,567 | protein_coding |
| 2 | BOVINEHD0200007602 | *U6* | 26,286,131 | 26,286,238 | snRNA |
| 4 | BOVINEHD0400014486 | *TES* | 52,169,927 | 52,224,938 | protein_coding |
| 4 | BOVINEHD0400014486 | *TFEC* | 52,282,331 | 52,499,410 | protein_coding |
| 5 | BOVINEHD0500031113 | *B4GALNT3* | 107,337,704 | 107,434,016 | protein_coding |
| 5 | BOVINEHD0500031113 | *NINJ2* | 107,436,495 | 107,440,389 | protein_coding |
| 5 | BOVINEHD0500031113 | *WNK1* | 107,577,010 | 107,706,757 | protein_coding |
| 5 | BOVINEHD0500031113 | *bta-mir-2437* | 107,556,526 | 107,556,586 | miRNA |
| 6 | BOVINEHD0600010408 | *FAM13A* | 35,695,188 | 36,031,040 | protein_coding |
| 6 | BOVINEHD0600010408 | *HERC3* | 36,052,150 | 36,198,478 | protein_coding |
| 6 | BOVINEHD0600010726 | *FAM184B* | 37,180,074 | 37,321,575 | protein_coding |
| 6 | BOVINEHD0600010726 | *DCAF16* | 37,309,247 | 37,332,605 | protein_coding |
| 6 | BOVINEHD0600010726 | *NCAPG* | 37,331,973 | 37,378,124 | protein_coding |
| 6 | BOVINEHD0600010726 | *LCORL* | 37,379,295 | 37,557,159 | protein_coding |
| 6 | BOVINEHD0600010408 | *NAP1L5* | 36,082,066 | 36,084,996 | protein_coding |
| 10 | BOVINEHD1000030754 | *SIX1* | 72,816,874 | 72,820,969 | protein_coding |
| 10 | BOVINEHD1000021008 | *PRKCH* | 73,436,691 | 73,705,998 | protein_coding |
| 10 | BOVINEHD1000030754 | *SIX4* | 72,876,280 | 72,886,021 | protein_coding |
| 10 | BOVINEHD1000030754 | *MNAT1* | 72,895,089 | 73,105,091 | protein_coding |
| 11 | BOVINEHD1100019768 | *LBH* | 69,617,501 | 69,655,066 | protein_coding |
| 11 | BOVINEHD1100006648 | *TMEM178A* | 21,962,641 | 22,020,613 | protein_coding |
| 11 | BOVINEHD1100006648 | *THUMPD2* | 22,040,726 | 22,080,776 | protein_coding |
| 16 | BOVINEHD1600006703 | *EPRS1* | 23,472,913 | 23,537,555 | protein_coding |
| 16 | BOVINEHD1600006703 | *BPNT1* | 23,557,430 | 23,600,661 | protein_coding |
| 16 | BOVINEHD1600006703 | *IARS2* | 23,586,935 | 23,631,281 | protein_coding |
| 16 | BOVINEHD1600006703 | *RAB3GAP2* | 23,622,910 | 23,735,636 | protein_coding |
| 16 | ARS_BFGL_NGS_68742 | *U2* | 10,725,782 | 10,725,938 | snRNA |
| 16 | BOVINEHD1600006703 | *bta-mir-194-1* | 23,607,933 | 23,608,029 | miRNA |
| 16 | BOVINEHD1600006703 | *bta-mir-215* | 23,607,646 | 23,607,724 | miRNA |
| 21 | BOVINEHD2100020541 | *TDRD9* | 68,471,518 | 68,582,287 | protein_coding |
| 21 | BOVINEHD2100020541 | *ASPG* | 68,618,021 | 68,641,438 | protein_coding |
| 21 | BOVINEHD2100020541 | *KIF26A* | 68,662,803 | 68,702,101 | protein_coding |
| 21 | BOVINEHD2100020541 | *MIR203B* | 68,645,094 | 68,645,177 | miRNA |
| 22 | BOVINEHD2200005412 | *GRM7* | 18,627,109 | 19,567,204 | protein_coding |
| 22 | BOVINEHD2200013355 | *CACNA2D3* | 45,924,535 | 46,818,582 | protein_coding |
| 24 | BOVINEHD2400014207 | *MAPK4* | 50,218,641 | 50,279,575 | protein_coding |
| 24 | BOVINEHD2400014207 | *MRO* | 50,318,709 | 50,343,196 | protein_coding |
| 29 | ARS_BFGL_NGS_14050 | *CCND1* | 46,888,664 | 46,898,541 | protein_coding |
| 29 | ARS_BFGL_NGS_14050 | *LTO1* | 46,897,059 | 46,917,393 | protein_coding |
| 29 | ARS_BFGL_NGS_14050 | *FGF19* | 46,937,865 | 46,942,875 | protein_coding |

**Table S15.** Candidate genes associated with significant SNPs for dry matter intake (DMI) in Nellore cattle at the medium environmental gradient (THI 74).

| BTA | SNP | Gene | Start gene position (bp) | End gene position (bp) | Gene biotype |
| --- | --- | --- | --- | --- | --- |
| 4 | BOVINEHD0400014486 | *TES* | 52,169,927 | 52,224,938 | protein_coding |
| 4 | BOVINEHD0400014486 | *TFEC* | 52,282,331 | 52,499,410 | protein_coding |
| 6 | BOVINEHD0600013007 | *CCKAR* | 45,920,952 | 45,932,166 | protein_coding |
| 6 | BOVINEHD0600013007 | *TBC1D19* | 46,022,662 | 46,173,614 | protein_coding |
| 6 | BOVINEHD0600012569 | *LGI2* | 44,589,569 | 44,618,174 | protein_coding |
| 6 | BOVINEHD0600012810 | *SMIM20* | 45,403,332 | 45,416,234 | protein_coding |
| 6 | BOVINEHD0600013061 | *STIM2* | 46,270,322 | 46,455,939 | protein_coding |
| 6 | BOVINEHD0600012569 | *SEPSECS* | 44,701,029 | 44,747,845 | protein_coding |
| 6 | BOVINEHD0600012810 | *SEL1L3* | 45,244,640 | 45,353,080 | protein_coding |
| 6 | BOVINEHD0600009626 | *CCSER1* | 33,015,656 | 34,501,208 | protein_coding |
| 6 | BOVINEHD0600010407 | *FAM13A* | 35,695,188 | 36,031,040 | protein_coding |
| 6 | BOVINEHD0600010407 | *HERC3* | 36,052,150 | 36,198,478 | protein_coding |
| 6 | BOVINEHD0600010726 | *FAM184B* | 37,180,074 | 37,321,575 | protein_coding |
| 6 | BOVINEHD0600010726 | *DCAF16* | 37,309,247 | 37,332,605 | protein_coding |
| 6 | BOVINEHD0600010726 | *NCAPG* | 37,331,973 | 37,378,124 | protein_coding |
| 6 | BOVINEHD0600010726 | *LCORL* | 37,379,295 | 37,557,159 | protein_coding |
| 6 | BOVINEHD0600010025 | *MMRN1* | 34,655,101 | 34,767,268 | protein_coding |
| 6 | BOVINEHD0600010407 | *NAP1L5* | 36,082,066 | 36,084,996 | protein_coding |
| 6 | BOVINEHD0600009439 | *U6* | 32,388,051 | 32,388,157 | snRNA |
| 6 | BOVINEHD0600008722 | *BMPR1B* | 29,373,117 | 29,605,410 | protein_coding |
| 6 | BTA_121735_NO_RS | *PDLIM5* | 29,910,305 | 30,142,223 | protein_coding |
| 6 | HAPMAP32447_BTC_033214 | *GRID2* | 30,780,009 | 32,378,893 | protein_coding |
| 6 | BTA_121735_NO_RS | *Y_RNA* | 29,990,590 | 29,990,701 | Y_RNA |
| 6 | BOVINEHD0600005354 | *DKK2* | 18,125,623 | 18,254,810 | protein_coding |
| 10 | BOVINEHD1000030754 | *SIX1* | 72,816,874 | 72,820,969 | protein_coding |
| 10 | BOVINEHD1000030754 | *SIX4* | 72,876,280 | 72,886,021 | protein_coding |
| 10 | BOVINEHD1000030754 | *MNAT1* | 72,895,089 | 73,105,091 | protein_coding |
| 11 | BOVINEHD1100019768 | *LBH* | 69,617,501 | 69,655,066 | protein_coding |
| 14 | BOVINEHD1400007161 | *XKR4* | 22,640,470 | 22,953,771 | protein_coding |
| 14 | BOVINEHD1400007161 | *TMEM68* | 23,027,993 | 23,070,071 | protein_coding |
| 14 | BOVINEHD1400007161 | *TGS1* | 23,070,145 | 23,095,949 | protein_coding |
| 22 | BOVINEHD2200005412 | *GRM7* | 18,627,109 | 19,567,204 | protein_coding |
| 22 | BOVINEHD2200013355 | *CACNA2D3* | 45,924,535 | 46,818,582 | protein_coding |
| 29 | ARS_BFGL_NGS_14050 | *CCND1* | 46,888,664 | 46,898,541 | protein_coding |
| 29 | ARS_BFGL_NGS_14050 | *LTO1* | 46,897,059 | 46,917,393 | protein_coding |
| 29 | ARS_BFGL_NGS_14050 | *FGF19* | 46,937,865 | 46,942,875 | protein_coding |

**Table S16.** Candidate genes associated with significant SNPs for dry matter intake (DMI) in Nellore cattle at the high environmental gradient (THI 81).

| BTA | SNP | Gene | Start gene position (bp) | End gene position (bp) | Gene biotype |
| --- | --- | --- | --- | --- | --- |
| 4 | BOVINEHD0400014486 | *TES* | 52,169,927 | 52,224,938 | protein_coding |
| 4 | BOVINEHD0400014486 | *TFEC* | 52,282,331 | 52,499,410 | protein_coding |
| 6 | BOVINEHD0600013007 | *CCKAR* | 45,920,952 | 45,932,166 | protein_coding |
| 6 | BOVINEHD0600013007 | *TBC1D19* | 46,022,662 | 46,173,614 | protein_coding |
| 6 | BOVINEHD0600012569 | *LGI2* | 44,589,569 | 44,618,174 | protein_coding |
| 6 | BOVINEHD0600013061 | *STIM2* | 46,270,322 | 46,455,939 | protein_coding |
| 6 | BOVINEHD0600012569 | *SEPSECS* | 44,701,029 | 44,747,845 | protein_coding |
| 6 | BOVINEHD0600009802 | *CCSER1* | 33,015,656 | 34,501,208 | protein_coding |
| 6 | HAPMAP26263_BTC_036885 | *FAM13A* | 35,695,188 | 36,031,040 | protein_coding |
| 6 | HAPMAP26263_BTC_036885 | *HERC3* | 36,052,150 | 36,198,478 | protein_coding |
| 6 | BOVINEHD0600010726 | *FAM184B* | 37,180,074 | 37,321,575 | protein_coding |
| 6 | BOVINEHD0600010726 | *DCAF16* | 37,309,247 | 37,332,605 | protein_coding |
| 6 | BOVINEHD0600010726 | *NCAPG* | 37,331,973 | 37,378,124 | protein_coding |
| 6 | BOVINEHD0600010726 | *LCORL* | 37,379,295 | 37,557,159 | protein_coding |
| 6 | BOVINEHD0600010032 | *MMRN1* | 34,655,101 | 34,767,268 | protein_coding |
| 6 | HAPMAP26263_BTC_036885 | *NAP1L5* | 36,082,066 | 36,084,996 | protein_coding |
| 6 | BOVINEHD0600009439 | *U6* | 32,388,051 | 32,388,157 | snRNA |
| 6 | BOVINEHD0600008722 | *BMPR1B* | 29,373,117 | 29,605,410 | protein_coding |
| 6 | HAPMAP32447_BTC_033214 | *GRID2* | 30,780,009 | 32,378,893 | protein_coding |
| 10 | BOVINEHD1000030754 | *SIX1* | 72,816,874 | 72,820,969 | protein_coding |
| 10 | BOVINEHD1000030754 | *SIX4* | 72,876,280 | 72,886,021 | protein_coding |
| 10 | BOVINEHD1000030754 | *MNAT1* | 72,895,089 | 73,105,091 | protein_coding |
| 11 | BOVINEHD1100019768 | *LBH* | 69,617,501 | 69,655,066 | protein_coding |
| 14 | BOVINEHD1400007161 | *XKR4* | 22,640,470 | 22,953,771 | protein_coding |
| 14 | BOVINEHD1400007161 | *TMEM68* | 23,027,993 | 23,070,071 | protein_coding |
| 14 | BOVINEHD1400007161 | *TGS1* | 23,070,145 | 23,095,949 | protein_coding |
| 22 | BOVINEHD2200005412 | *GRM7* | 18,627,109 | 19,567,204 | protein_coding |
| 22 | BOVINEHD2200013355 | *CACNA2D3* | 45,924,535 | 46,818,582 | protein_coding |
| 29 | ARS_BFGL_NGS_14050 | *CCND1* | 46,888,664 | 46,898,541 | protein_coding |
| 29 | ARS_BFGL_NGS_14050 | *LTO1* | 46,897,059 | 46,917,393 | protein_coding |
| 29 | ARS_BFGL_NGS_14050 | *FGF19* | 46,937,865 | 46,942,875 | protein_coding |
